# Designer indicators for two-photon recording of subthreshold voltage dynamics

**DOI:** 10.1038/s41592-026-03043-8

**Published:** 2026-04-15

**Authors:** Michelle A. Land, Mario Galdamez, Vincent Villette, Jun Zhu, Xiaoyu Lu, Mate Marosi, Shuyuan Yang, Gregory Foran, Alex J. McDonald, Xiaoyu Dong, Elsayed Zaabout, Haixin Liu, Zhuohe Liu, Kevin L. Colbert, Shujuan Lai, Matthew Shorey, Anthony S. G. Lourdiane, Annick Ayon, Jonathan Bradley, Caroline Mailhes-Hamon, Ryan G. Natan, Jian Zhong, Ryan Kroeger, Robert G. Law, Noura Hakam, Cameron L. Smith, Ming Hu, Shanii Tabb, Brice Bathellier, Barna Dudok, Na Ji, Laurent Bourdieu, Jacob Reimer, François St-Pierre

**Affiliations:** 1 Department of Neuroscience, Baylor College of Medicine, Houston, TX USA; 2 Institut de Biologie de l’Ecole Normale Supérieure (IBENS), Ecole Normale Supérieure, CNRS, INSERM, Université PSL, Paris, France; 3 Department of Neuroscience, University of California, Berkeley, Berkeley, CA USA; 4 Systems, Synthetic and Physical Biology program, Rice University, Houston, TX USA; 5 Department of Neurology, Baylor College of Medicine, Houston, TX USA; 6 Department of Chemical and Biomolecular Engineering, Rice University, Houston, TX USA; 7 Université Paris Cité, Institut Pasteur, AP-HP, INSERM, CNRS, Fondation Pour l’Audition, Institut de l’Audition, IHU reConnect, Paris, France; 8 Department of Physics, University of California, Berkeley, Berkeley, CA USA; 9 Helen Wills Neuroscience Institute, University of California, Berkeley, Berkeley, CA USA; 10 Molecular Biophysics and Integrated Bioimaging Division, Lawrence Berkeley National Laboratory, Berkeley, CA USA; 11 Department of Electrical and Computer Engineering, Rice University, Houston, TX USA; 12 Center for Neuroscience and Artificial Intelligence, Baylor College of Medicine, Houston, TX USA; 13 Department of Biochemistry and Molecular Pharmacology, Baylor College of Medicine, Houston, TX USA

**Keywords:** Fluorescent proteins, Fluorescence imaging, Multiphoton microscopy, High-throughput screening

## Abstract

Subthreshold voltage dynamics are essential for neuronal information integration, yet they remain technically challenging to measure in vivo. While genetically encoded voltage indicators are emerging as powerful tools for voltage recording, they lack the sensitivity to detect millivolt-scale subthreshold fluctuations with two-photon microscopy—the method of choice for deep-tissue recording. To overcome this limitation, we engineered two genetically encoded voltage indicators, JEDI3sub and JEDI3hyp, with enhanced subthreshold voltage detection under two-photon excitation. In the mouse brain, JEDI3sub enabled simultaneous tracking of subthreshold optical tuning from over 100 cells simultaneously, while JEDI3hyp captured subthreshold dynamics associated with sharp-wave ripples in hippocampal PV^+^ interneurons. Moreover, JEDI3hyp supported prolonged imaging of brain-state-dependent, millivolt-scale subthreshold voltage changes across deep-layer somas, fine dendritic structures and diverse cell types. By enabling sensitive reporting of subthreshold voltage dynamics, JEDI3 indicators open previously unexplored avenues for dissecting neural information processing in health and disease.

## Abstract (web summary)

The genetically encoded voltage indicators JEDI3sub and JEDI3hyp enhance monitoring of subthreshold neuronal activity in the awake mouse brain using a variety of two-photon microscopy techniques.

## Main

Neurons process and transmit information by regulating membrane voltage. At synapses, neurotransmitters produce subthreshold voltage changes that propagate through dendrites, where they are filtered, attenuated or amplified. These signals converge at the axon initial segment near the cell body, where spiking neurons convert them into all-or-none voltage events known as action potentials. At the circuit level, functional brain states can shape network dynamics by generating synchronized subthreshold fluctuations and oscillations, priming neurons for coordinated spiking^1^. Monitoring subthreshold dynamics with subcellular resolution and across neuronal ensembles is thus essential for uncovering the principles of neuronal computations^2–4^.

Although spiking activity can be readily monitored in vivo, capturing subthreshold voltage dynamics in behaving animals remains technically challenging. For instance, although Neuropixels^5^ electrophysiological probes are highly effective for recording spiking activity, they do not provide reliable access to subthreshold membrane potential dynamics at the single-neuron level. Genetically encoded calcium indicators enable cell-type-specific imaging of activity across large brain volumes. However, they primarily reflect spiking-related calcium influx and are inherently ill-suited for reporting the full range of subthreshold voltage changes—although they may detect large depolarizations in some preparations^6^. These limitations have motivated the use of voltage-sensitive dyes^7–9^, which can report subthreshold activity with single-cell and population-level resolution. However, unless delivered via intracellular injection to individual cells, these dyes indiscriminately stain all membranes, resulting in high background fluorescence from the neuropil that compromises high-fidelity optical voltage recording. Moreover, they typically cannot be restricted to specific cell types, complicating efforts to tease apart the contributions of genetically defined neuronal populations to network computations.

Genetically encoded voltage indicators (GEVIs) are protein-based biosensors whose fluorescence is modulated by membrane potential. GEVIs offer a powerful alternative for monitoring voltage dynamics, including subthreshold fluctuations. They can be targeted to genetically defined neuronal populations using cell-type-specific promoters, and background neuropil fluorescence can be minimized through sparse labeling strategies or soma-localization tags. GEVIs have enabled optical recordings of population-level voltage dynamics^10^ and subthreshold fluctuations in somas and dendrites in dissociated cultures^11,12^, brain slices^13^ and in vivo^14–17^.

Multiple GEVI studies have leveraged one-photon (1P) microscopy techniques, which provide high signal-to-noise ratios (SNRs) but are limited in imaging depth. To overcome this challenge, two-photon (2P) microscopy—the most widely used noninvasive technique for deep-tissue imaging in neuroscience—has been combined with GEVIs to visualize diverse subthreshold signals in the mouse cortex, including up/down states in somas^18^, isolated dendritic depolarizations^19^ and trial-averaged voltage changes in spines^20^. However, under 2P excitation, the SNR for subthreshold responses remains limited, restricting the reliable detection of these signals to a narrow set of experimental conditions. To expand the range and robustness of in vivo experiments tracking subthreshold voltage dynamics with 2P microscopy, a new generation of GEVIs is urgently needed.

Here we present advancements in our 2P high-throughput indicator screening platform, enabling the evolution of GEVIs optimized for detecting subthreshold voltage dynamics. This system yielded two GEVIs, JEDI3sub and JEDI3hyp, each with enhanced responses to subthreshold voltages and distinct ranges of maximal membrane potential sensitivity. The JEDI3 sensors reliably captured subthreshold changes during spontaneous activity, stimulus-driven responses and hippocampal short-wave ripples. Notably, JEDI3hyp tracked millivolt-scale, brain-state-dependent somatic and dendritic subthreshold voltage fluctuations in layer 5 pyramidal neurons. Together, the JEDI3 indicators enabled robust monitoring of subthreshold voltage dynamics across various brain regions, cell types and imaging modalities.

## Results

### Multiparametric high-throughput optimization of GEVIs for 2P recording of subthreshold voltage dynamics

Owing to its demonstrated ability to perform sustained deep-tissue 2P spike recordings in vivo, we selected JEDI-2P as the base sensor to create an indicator with greater sensitivity to subthreshold voltage changes^18^. Our aim was to enhance JEDI-2P’s responsiveness to subthreshold voltages while preserving or improving other critical properties. We used a 2P multiparametric high-throughput screening platform that measures the sensors’ responses to brief (~10 ms) cellular voltage signals, brightness and photostability^18^. We expressed sensor variants in HEK293-Kir2.1 cells, maintained at a resting membrane potential of ~−83 mV using an extracellular solution containing 5 mM potassium. This potential was chosen because mammalian neurons rarely hyperpolarize below −85 mV^21^. Cells were depolarized using electrical field stimulations of varying strengths, hypothesizing that selecting variants with pronounced responses to weak stimulations (Supplementary Fig. 1a) would yield GEVIs optimized for detecting subthreshold voltage changes.

We screened nearly 100 libraries arrayed in 96-well plates, including 79 targeting single sites by saturation mutagenesis and 17 combining promising mutations from the single-site libraries. Of the 79 mutated residues, 48 resided in the voltage-sensing domain, and 31 in the GFP moiety (Supplementary Fig. 1b). The two best-performing indicators demonstrated larger responses to both partial and maximal field stimulations compared with JEDI-2P, while retaining comparable brightness and photostability (Supplementary Fig. 1c,d). We refer to these two sensors as Jellyfish-derived Electricity-reporting Designer Indicator, 3rd-generation (JEDI3), with JEDI3sub optimized for detecting subthreshold fluctuations and JEDI3hyp exhibiting a hyperpolarized high-sensitivity range relative to JEDI3sub.

**Fig. 1:**
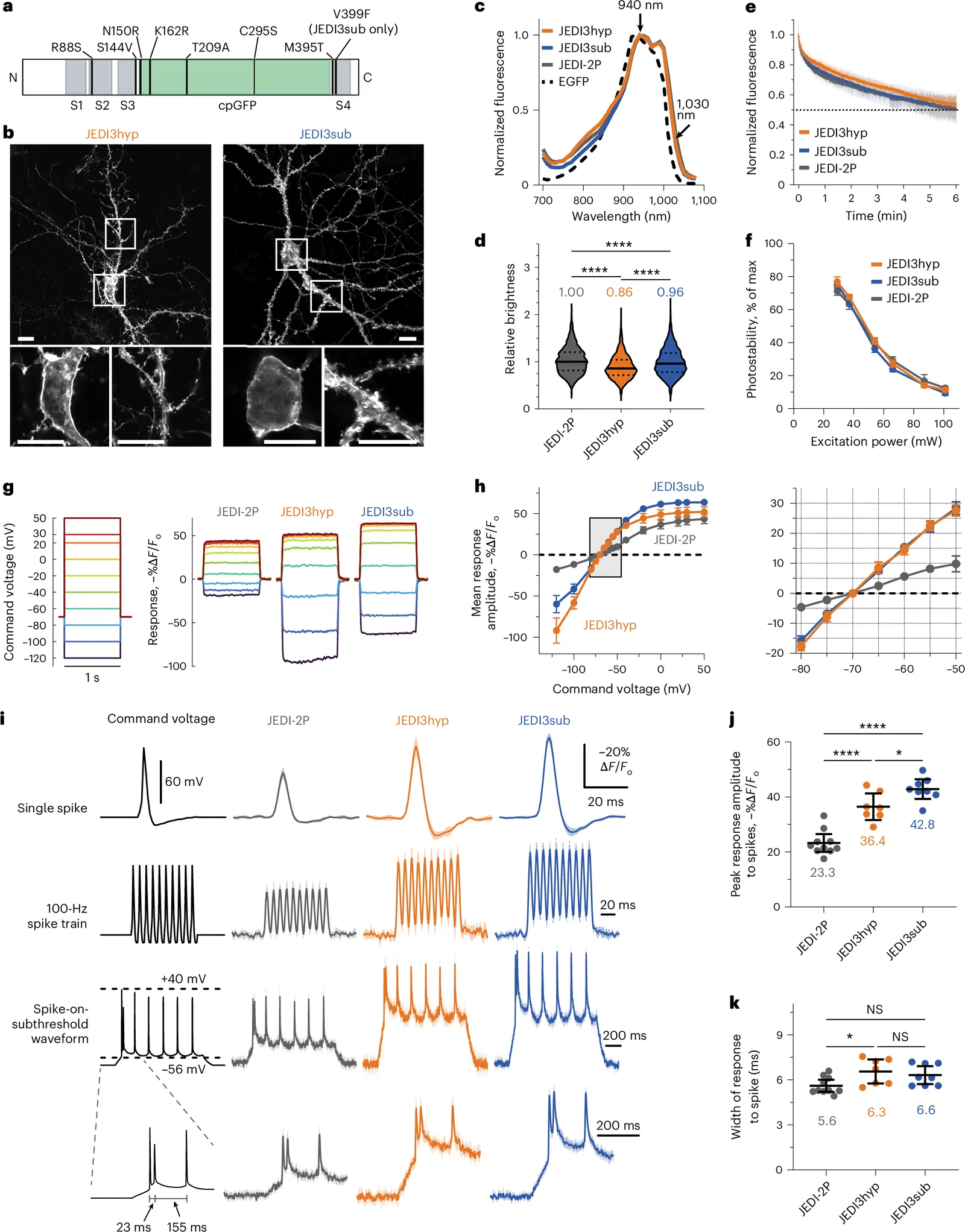
JEDI3 indicators exhibit enhanced sensitivity to subthreshold voltages while maintaining comparable brightness and photostability as JEDI-2P in vitro*.* **a**, JEDI3hyp and JEDI3sub mutations relative to the parental JEDI-2P sequence. Gray, transmembrane segments of the voltage-sensing domain. Green, the circularly permuted GFP domain. **b**, Representative example of cultured rat E18 hippocampal neurons expressing JEDI3 indicators. Soma zoom-in images were taken from single confocal *z*-slices, while other images are confocal *z*-stack maximal projections. Scale bars, 10 μm. **c**, 2P excitation spectra in HEK293-Kir2.1 cells. Spectra were normalized to their respective peaks. Laser pulses were not pre-compensated for dispersion in the microscope optical path. Data are the mean of *n* = 14 FOVs per construct; each FOV is an independent transfection containing >100 cells. **d**, Violin plots of GEVI brightness at ~−70 mV. Sensor fluorescence was normalized to the brightness of the covalently linked mBeRFP and displayed relative to JEDI-2P’s median brightness. Black lines denote the median, with exact values displayed above the plots. Dashed lines indicate quartile splits. Brightness from *n* = 5,165 (JEDI-2P), *n* = 4,803 (JEDI3hyp), *n* = 4,699 (JEDI3sub) cells; data are from eight independent transfections per sensor into HEK293-Kir2.1 cells. ****P < 0.0001, Kruskal–Wallis test with Dunn’s multiple-comparisons test. **e**, Representative 6-min photobleaching trace using 37-mW excitation light at the sample plane. *n* = 3 (JEDI-2P) or 4 (other GEVIs) independent transfections. **f**, Photobleaching at various excitation powers. Each data point represents the mean integrated fluorescence signal over a 6-min time course at the corresponding power level, displayed as a percentage of the total signal that would be produced by an ideal, non-bleaching sensor. *n* = 4 independent transfections per indicator in HEK293-Kir2.1 cells, except for *n* = 3 for 29 and 37 mW for JEDI-2P. The area under the curve of the fluorescence traces at different powers was significantly different between JEDI3sub and JEDI-2P (*P* < 0.05, Kruskal–Wallis test, followed by a Dunn’s multiple-comparisons test). **g**–**k**, Fluorescence responses to voltage. *n* = 10 (JEDI-2P), 7 (JEDI3hyp) and 8 (JEDI3sub) transfected HEK293A cells. **g**, Fluorescence responses to voltage steps. Individual traces were averaged and the resulting mean trace was smoothed by a 47.6-ms moving average. **h**, Mean response amplitudes to voltage steps. Right: magnified view of subthreshold values. **i**, Mean responses to spike waveforms. To mimic the properties of layer 2/3 cortical neurons at room temperature, single spikes and action potentials in trains had a 2-ms FWHM^46^. The bottom-row waveform was adapted from a recording of mouse L5 pyramidal neurons and displays slightly wider spikes (3–4 ms). **j**, Peak fluorescence response amplitude to single-spike waveforms. *P < 0.05; ****P < 0.0001. One-way analysis of variance (ANOVA) followed by Tukey’s multiple-comparisons test. **k**, Width of responses to single-spike waveforms, quantified as the FWHM. *P < 0.05; non-parametric Kruskal–Wallis test followed by Dunn’s multiple comparison. In all panels, unless indicated otherwise, data were collected at 23 °C using 940 nm light, the center (darker) traces represent the mean, and error bars or shaded areas denote the 95% CI. In **e**,**f** and **g**–**k**, imaging was acquired by resonant scanning at 440 Hz. Supplementary Table 2 provides additional statistical information. Data were acquired in HEK cells, except for **b**. NS, not significant. Source data

JEDI3hyp and JEDI3sub differ from JEDI-2P by seven and eight mutations, respectively (Fig. 1a and Supplementary Fig. 1b). Three mutations are in the voltage-sensing domain: Ser144Val and Met395Thr, located in extracellular residues near the cpGFP insertion site, and Arg88Ser, positioned N-terminally to the S2 segment. Four mutations are in the GFP domain and were screened for their prevalence in other GEVIs or for their functional importance. Of these, Asn150Arg (GFP Asn146) lies near both the chromophore and a GFP-VSD junction and was also identified during development of the GEVI ASAP3 (ref. ^22^). Lys162Arg emerged from screens of mutations from the glutamate indicator iGluSnFr^23^. Thr209Ala (GFP Thr205) may influence excited-state proton transfer^24^. Cys295Ser (GFP Cys48) modifies a β-barrel cysteine, potentially reducing aberrant folding in the endoplasmic reticulum^25^. Lastly, the Val399Phe mutation was identified in libraries aimed at shifting JEDI3hyp’s sensitivity to more polarized potentials, thereby aligning JEDI3hyp more closely with the physiological range of neuronal voltages^26^. Residue 399 was tested because it lies at the N terminus of S4, a transmembrane segment critical for voltage sensing^27^. Confocal microscopy qualitatively indicated that these mutations did not prevent JEDI3 indicators’ expression at the plasma membrane of dissociated rat hippocampal neurons (Fig. 1b and Supplementary Fig. 2), supporting further characterization.

### In vitro 2P characterization of JEDI3hyp and JEDI3sub in mammalian cells

We first determined the 2P excitation spectra of JEDI3hyp and JEDI3sub (Fig. 1c). Both indicators exhibited excitation profiles similar to those of JEDI-2P, with a peak at 940 nm and a broad spectrum that maintained over 85% of the peak excitation between 920 nm and 1,000 nm. All JEDI indicators demonstrated substantial excitation (25–32%) at 1,030 nm, enabling compatibility with fiber lasers used in emerging 2P techniques such as scanless^28^, SLAP^29^ and free-space angular-chirp-enhanced delay (FACED)^30,31^ microscopy. We performed the remaining in vitro characterization at the indicators’ peak excitation wavelength of 940 nm.

We previously demonstrated that JEDI-2P exhibits high brightness and photostability, supporting continuous in vivo recordings for over 30 min across various 2P microscopy techniques^18^. JEDI3sub maintained brightness comparable to JEDI-2P at ~−70 mV, while JEDI3hyp retained 86% of JEDI-2P’s brightness (Fig. 1d). All JEDI variants displayed bi-exponential photobleaching kinetics, characterized by an initial rapid decay followed by a slower phase (Fig. 1e), and exhibited similar photostability across a range of excitation power levels (Fig. 1f).

We then assessed whether JEDI3 indicators displayed enhanced responses to subthreshold voltage changes, as predicted by our screens. Using 2P voltage imaging combined with whole-cell voltage clamp from a resting membrane potential of −70 mV, JEDI3hyp and JEDI3sub produced 2.6-fold and 2.7-fold larger responses to 10-mV depolarizations, respectively, compared with JEDI-2P; they also exhibited 3.6-fold and 3.4-fold larger responses to 10-mV hyperpolarizations (Fig. 1g,h and Supplementary Fig. 3). Responses to 100-mV voltage steps from −70 mV were also significantly larger: JEDI3hyp and JEDI3sub produced responses of 51.5% and 63.5%, respectively, compared with 41.8% for JEDI-2P (Supplementary Fig. 3 and Supplementary Table 2).

We quantified indicator kinetics to assess the effectiveness of GEVI fluorescence in tracking voltage dynamics. Fast depolarization kinetics are particularly critical for producing large responses to spikes. For voltage steps corresponding to suprathreshold events (−70 mV or −45 mV to +30 mV), JEDI3hyp and JEDI3sub maintained the rapid response time constants of JEDI-2P (Table 1). For subthreshold depolarizations (−70 mV to −45 mV) and hyperpolarization (−70 mV to −100 mV) steps, JEDI3hyp and JEDI3sub exhibited slightly slower kinetics (1–2 ms more) than JEDI-2P. Notably, the kinetic rates of indicators derived from identical or orthologous voltage-sensing domains exhibit an increase with rising temperature^18,32^. Thus, indicators are expected to respond faster at physiological temperatures in mice than at 33 °C—the temperature used here due to challenges in maintaining robust patch seals at higher temperatures. Note that, unlike all other assays in this section, kinetic measurements were performed under widefield 1P illumination, which remains the current standard in the field due to technical constraints previously described^18^.

**Table 1:** Indicator kinetics at 33 °C

	JEDI-2P	JEDI3hyp	JEDI3sub
**−70** **mV to** **+30** **mV**			
Tau fast (ms)	0.55 ± 0.06	0.48 ± 0.05	0.63 ± 0.05
Tau slow (ms)	7.57 ± 2.66	7.97 ± 2.17	5.31 ± 1.39
Fast component (%)	85.1 ± 3.5	90.3 ± 1.9	90.8 ± 1.4
**+30** **mV to −70** **mV**			
Tau (ms)	1.20 ± 0.11	2.11 ± 0.21	2.23 ± 0.19
**−45** **mV to** **+30** **mV**			
Tau (ms)	1.36 ± 0.15	1.69 ± 0.45	1.18 ± 0.15
**+30** **mV to −45** **mV**			
Tau (ms)	1.68 ± 0.12	2.90 ± 0.52	2.92 ± 0.22
**−70** **mV to −45** **mV**			
Tau (ms)	1.04 ± 0.06	2.59 ± 0.39	2.25 ± 0.17
**−45** **mV to −70** **mV**			
Tau (ms)	0.96 ± 0.18	3.00 ± 0.35	2.45 ± 0.14
**−100** **mV to −70** **mV**			
Tau (ms)	1.46 ± 0.23	3.14 ± 0.35	2.53 ± 0.17
**−70** **mV to −100** **mV**			
Tau (ms)	1.04 ± 0.24	3.43 ± 0.26	2.27 ± 0.10

Values are the time constants of the optical response to voltage steps, expressed as means ± 95% confidence interval (CI). For voltage steps from −70 mV to +30 mV, and +30 mV to −70 mV: *n* = 7 (JEDI-2P), 15 (JEDI3hyp) and 10 (JEDI3sub) cells. For other voltage steps: *n* = 9 (JEDI-2P), 8 (JEDI3hyp) and 10 (JEDI3sub) cells. HEK293A cells were used.

Source data

Next, we assessed how JEDI3 kinetics and steady-state responses influenced spike and subthreshold detectability. JEDI3hyp and JEDI3sub produced peak responses to 2-ms full-width at half-maximum (FWHM) action potential waveforms that were 56% and 84% larger, respectively, than JEDI-2P (Fig. 1i,j). JEDI3hyp produced slightly longer responses to 2-ms FWHM action potential waveforms when compared with JEDI3sub and JEDI-2P (Fig. 1k). All JEDIs reliably tracked 100-Hz spike train waveforms (Fig. 1i), consistent with their overall rapid kinetics. When tested with spike-on-subthreshold waveforms originally recorded from mouse layer 5 pyramidal neurons, JEDI3 sensors exhibited markedly enhanced responses to subthreshold components compared with JEDI-2P (Fig. 1i).

**Extended Data Fig. 1:**
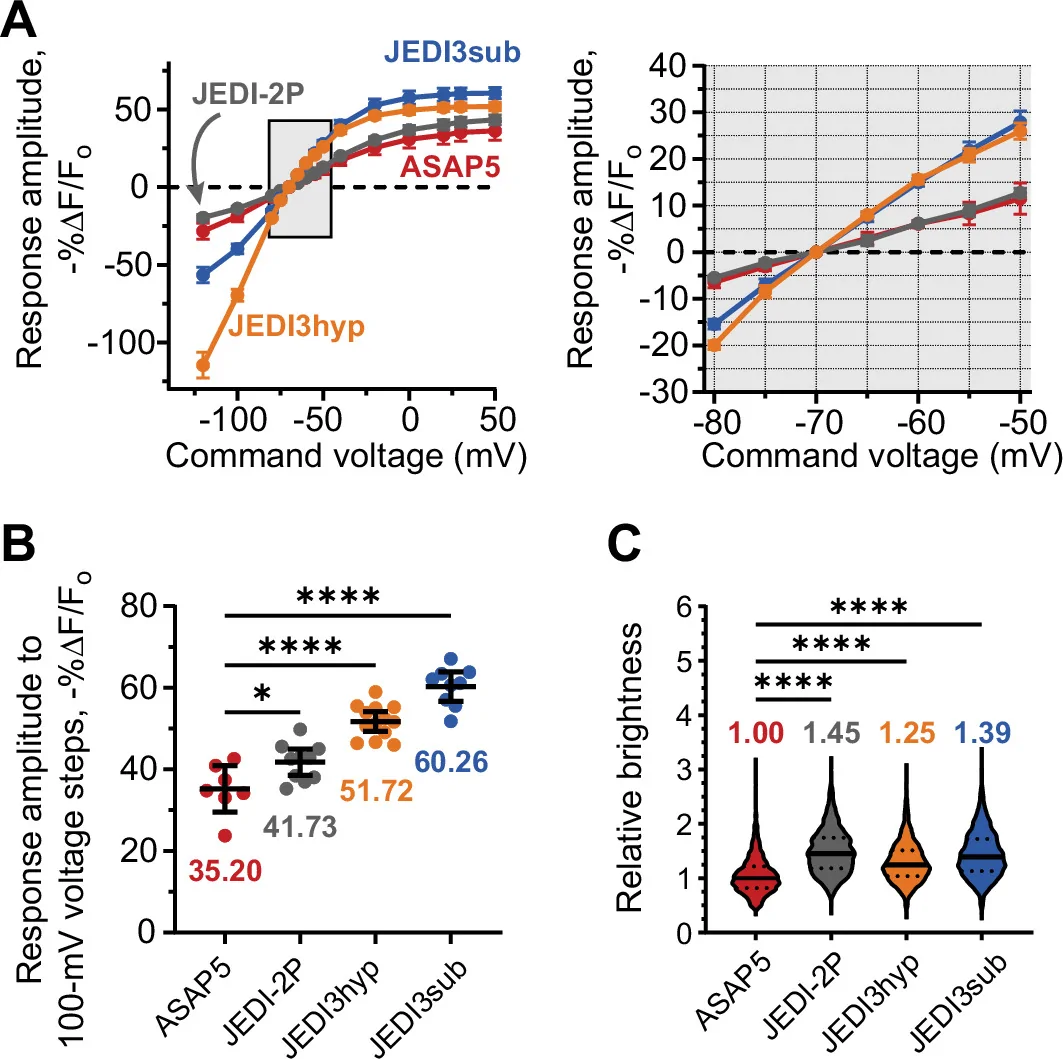
JEDI3 indicators exhibit greater voltage sensitivity and brightness than ASAP5 under two-photon illumination in HEK293 cells. (**A**) Mean fluorescence responses to voltage steps at 33⁰C. Same 33⁰C data in Supplementary Fig. 3A-C compared with 33⁰C ASAP5 data (n = 7 cells). *Right panel*, additional quantification of smaller voltage steps around subthreshold levels. Imaging was conducted using two-photon resonant scanning at 940 nm and a framerate of 440 Hz. Voltage clamp was performed in HEK293A cells. (**B**) Mean responses to 100-mV voltage steps from a resting potential of −70 mV compared with JEDI sensors. One-way ANOVA followed by Dunnett multiple comparisons test was performed. *p < 0.05, ****p < 0.0001. (**C**) Brightness of GEVIs at ~-70 mV in HEK293-Kir2.1 cells. Sensor fluorescence was normalized to the brightness of the covalently linked mBeRFP and then normalized to the median of ASAP5. Black lines denote the normalized median values, and dotted lines are the quartile splits. n ≥ 3,500 cells per indicator, analyzed from 8 independent wells/transfections per sensor. ****p < 0.0001, Kruskal-Wallis test with Dunn’s multiple comparisons test. For all figures, error bars denote 95% CI. Source data

Finally, we compared the 2P performance of JEDI indicators with ASAP5, a recently reported indicator^11^. ASAP5 exhibited voltage sensitivity comparable to JEDI-2P; however, its response was reduced relative to the JEDI3 indicators, particularly in the subthreshold regime. In response to 10-mV depolarizations and hyperpolarizations from −70 mV, ASAP5 produced signals ~0.4-fold those of the JEDI3 sensors (Extended Data Fig. 1a,b). When excited at 940 nm and expressed in cells with resting membrane potentials of −70 mV (±1 mV s.d.; *n* = 4), ASAP5 also showed reduced brightness relative to all JEDI variants (Extended Data Fig. 1c).

### JEDI3 indicators exhibit greater responses to subthreshold voltage fluctuations than JEDI-2P and ASAP5 in awake, behaving mice

**Fig. 2:**
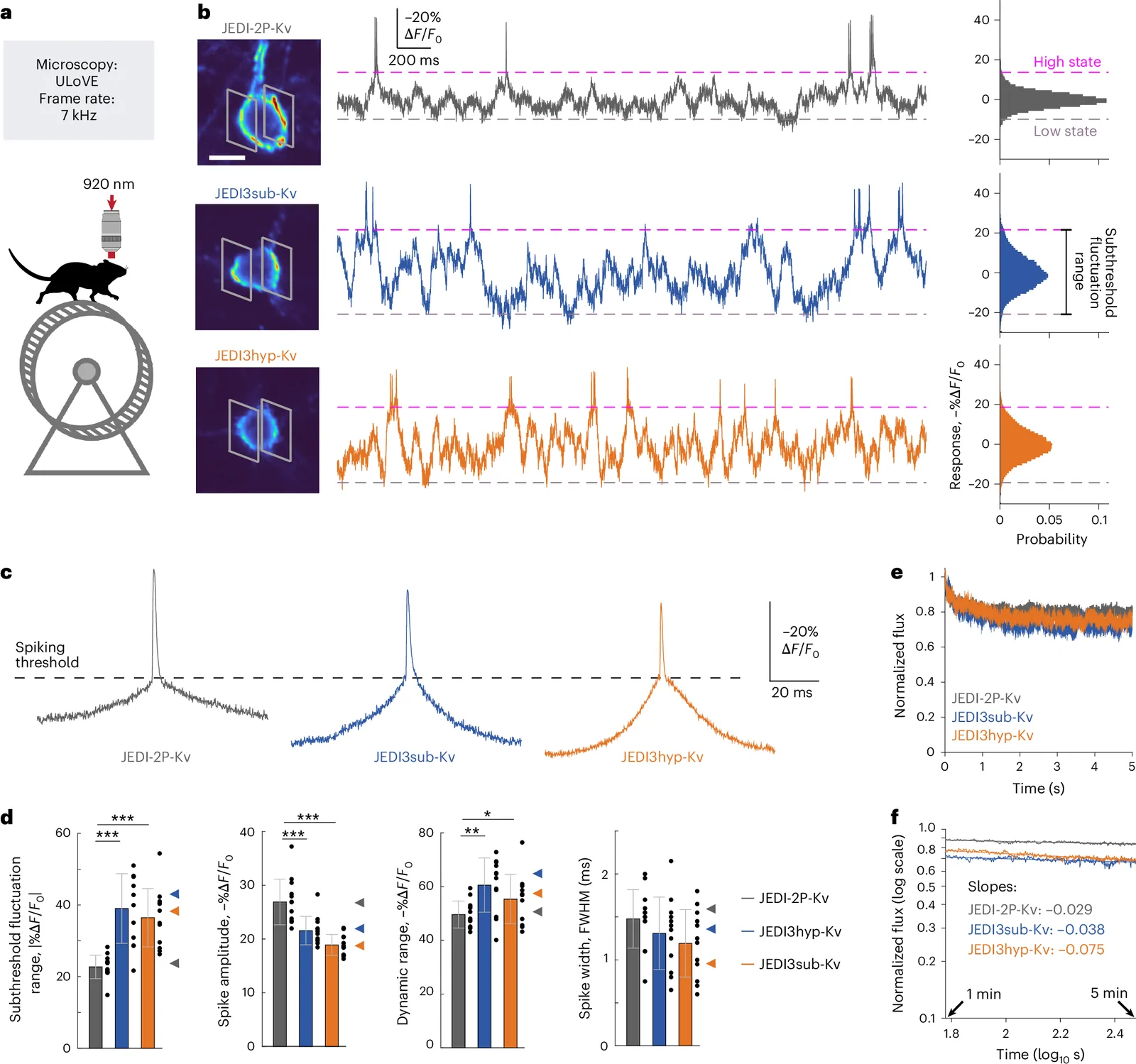
JEDI3 indicators report subthreshold dynamics with greater amplitude than JEDI-2P in cortical neurons of awake, behaving mice. **a**, Experimental design schematics. Mice were head-fixed and allowed to run on a freely moving wheel while 2P-induced fluorescence was recorded using ULoVE microscopy. Created in BioRender; St-Pierre, F. https://biorender.com/hm5yzl3 (2025). **b**, Representative responses from layer 2/3 neurons transduced using AAVs with Cre-dependent GEVI expression. Soma-restricted expression was achieved using the Kv motif. The two gray polygons in each image (left) represent the approximate boundaries of the ULoVE excitation patterns. Scale bar, 10 µm. Magenta and gray dashed lines represent cutoffs for high and low states, respectively, of subthreshold fluctuations. Histograms (right) represent probability distributions of the low-pass-filtered signal traces. **c**, Mean optical spike waveforms computed by averaging aligned traces containing isolated spikes from the recordings shown in **b**. *n* = 91 (JEDI-2P-Kv), 115 (JEDI3sub-Kv) and 403 (JEDI3hyp-Kv) spikes. **d**, Quantitative GEVI evaluation. *n* = 13 (JEDI-2P-Kv), 12 (JEDI3sub-Kv) and 12 (JEDI3hyp-Kv). *P < 0.05, **P < 0.01, ***P < 0.001; two-sample Wilcoxon rank-sum test (Supplementary Table 2). Bars represent the mean, and error bars represent the s.d. Arrowheads indicate which cell was used for representation in **b** and **c**. The subthreshold fluctuation range values exceed the amplitudes observed in the mean optical waveforms (**c**) because the local average waveform cannot display the entire subthreshold dynamic as spikes generally arise from a depolarized baseline. **e**,**f**, Photostability of JEDI3 indicators during the initial fast bleaching phase (**e**) and the remaining fluorescence trace (**f**). Sample sizes as in **d**. Source data

**Extended Data Fig. 2:**
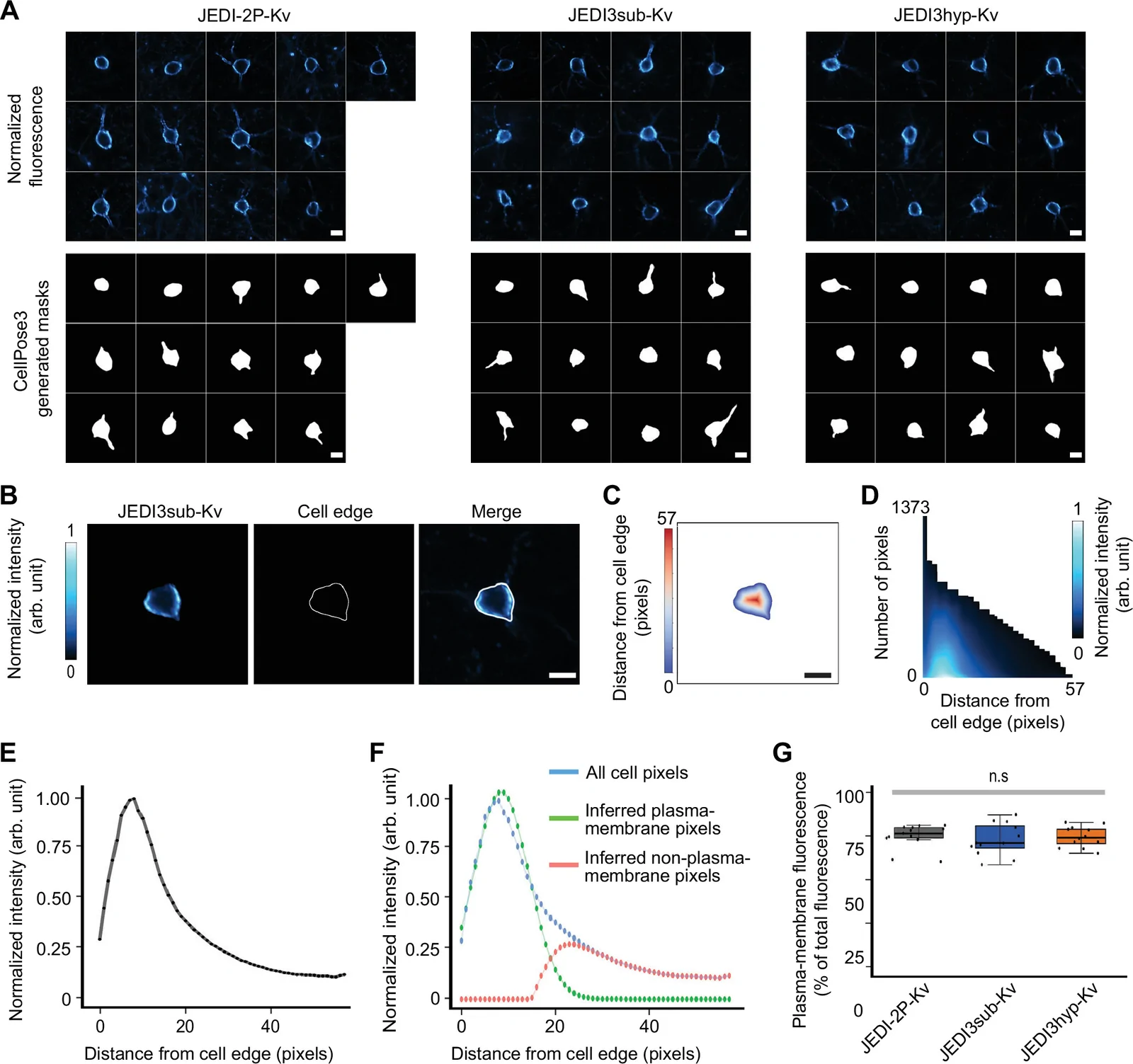
JEDI3hyp-Kv, JEDI3sub-Kv, and JEDI-2P-Kv showed comparable plasma membrane localization in mice. (**A**) *Top*, fluorescence images of the cortical neurons recorded in awake behaving mice for Fig. 2. Intensity values for each cell were normalized to that cell’s brightest pixel. *Bottom*, Cellpose3-generated whole-cell masks. (**B**) Representative Laplacian-filtered Cellpose3 mask of a JEDI3sub-Kv-expressing cell (left) and the corresponding computed cell edge (middle). (**C**) Euclidean distance transformation heat map of the cell in panel **B**. (**D**) Pixel intensity remapped as a function of distance from the cell edge. (**E**) Intensity profile of a single cell derived from panel **D**. (**F**) Overlay of raw trace from panel **E** (blue) with a Gaussian fit to the inferred plasma membrane component (green; R² = 0.981). The residual (red) represents the inferred non-membrane signal contribution. (**G**) Box plots showing mean plasma membrane-associated fluorescence percentages of 75 ± 7%, 75 ± 9%, and 73 ± 6%, respectively (mean ± s.d.), with no significant differences between variants (ANOVA: F(2, 22) = 0.187, p = 0.831). For all relevant figures, scale bars represent 10 µm. Source data

GEVI performance was evaluated in vivo using ultrafast local volume excitation (ULoVE)^22^, a 2P microscopy technique that utilizes acousto-optic deflectors (AODs) to acquire GEVI signals from single cells with high temporal resolution (>7.1 kHz) and sensitivity. We recorded layer 2/3 cortical neurons sparsely expressing GEVIs at depths from 69 µm to 304 µm (mean ± s.d.: 177 µm ± 66 µm; *n* = 37 cells from eight mice) in head-fixed, behaving animals (Fig. 2a,b). To enrich GEVIs at the soma and reduce background fluorescence from the neuropil, indicators were fused at their C terminus with the proximal restriction and clustering sequence from the Kv2.1 potassium channel^33^, denoted by ‘-Kv’ in their names. Efficient membrane targeting is essential, as intracellularly retained sensors may fluoresce but typically fail to report membrane voltage changes, thereby reducing response amplitude. Quantitative image analysis revealed that JEDI3sub-Kv, JEDI3hyp-Kv and JEDI-2P-Kv exhibited comparable levels of plasma-membrane fluorescence (Extended Data Fig. 2), indicating that the JEDI3 mutations did not alter subcellular localization relative to JEDI-2P-Kv.

Because the neurons under study exhibited ongoing activity (Fig. 2b), we could not quantify subthreshold changes as discrete, event-like changes from a fixed baseline. Instead, we characterized the overall distribution of signals below the spiking threshold. We defined the subthreshold fluctuation range as the difference between the median value of the spiking threshold and the lowest 1% percentile of the response amplitude signal (Fig. 2b). Suprathreshold (spike) response amplitudes (Fig. 2d) were derived from spike-triggered averages (Fig. 2c; signals above the dashed line).

**Extended Data Fig. 3:**
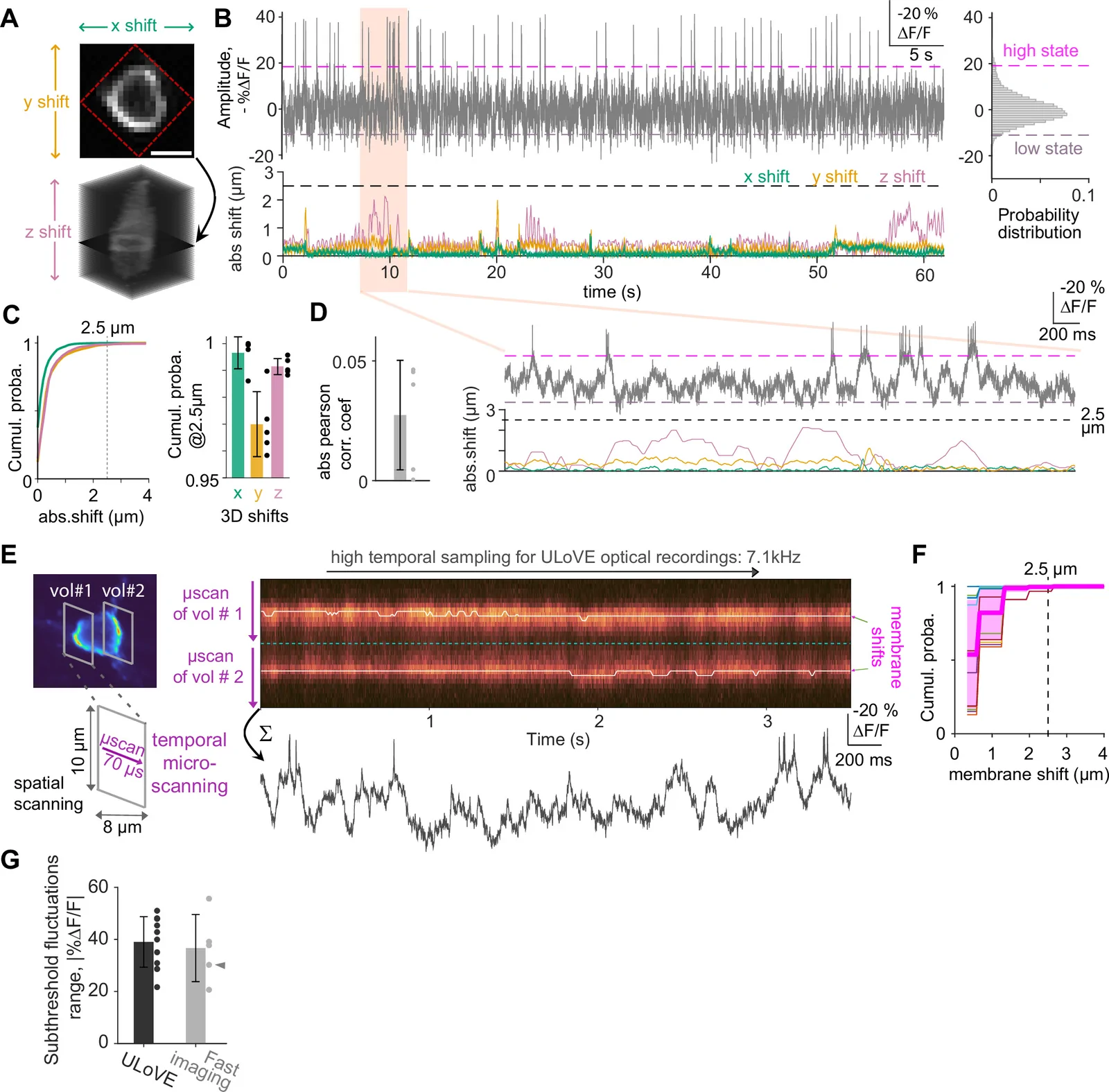
Distinguishing between genuine voltage signals and motion artefacts in JEDI3sub-Kv recordings obtained by fast imaging and ULoVE microscopy. (**A**) *Top*: Representative layer-2/3 cortical neuron expressing JEDI3sub-Kv. The image was acquired by averaging 50 frames acquired by raster scanning with Acousto-Optic Deflectors (AOD) at a rate of 2.66 kHz. The dashed red square in the upper image indicates the area scanned. Scale bar, 6 µm. *Bottom*, z-stack of the recorded neuron, displayed with an oblique angle. Inter-plane distance is 0.5 µm. The arrow shows the location of the top image’s plane. The z-stack was used to estimate motion-induced shifts in three dimensions. (**B**) *Top:* One-minute recording from the layer-2/3 JEDI3sub-Kv-expressing neuron shown in A. Magenta and purple dashed lines represent high and low subthreshold states, respectively. The histogram represents the probability distribution of the low-pass filtered response amplitudes. *Bottom:* x, y, and z shifts during the recording. The dashed line indicates the 2.5 µm threshold as defined in panel **C**. The pale beige rectangle shows the timing of the zoomed-in recording section shown below. (**C**) *Left:* Cumulative probability distributions of 3D shifts (x, green; y, yellow; z, purple) over the full recording of the representative neuron shown in **A**. The dashed line indicates the 2.5-µm threshold. *Right*: Bar graphs of the cumulative probability for shifts below 2.5 µm (mean ± s.d., data points are individual cells (n = 5). (**D**) Bar indicates mean ± s.d. (n = 5 cells) of the Pearson correlation coefficient between the average fluorescence signal and the Euclidean norm of the 3D shifts for shifts below 2.5 µm. (**E**) Measurement of cell shifts. *Top left*: Image of the recorded neuron acquired before the optical recordings shown on the right. Following our standard protocol for ULoVE recordings of individual cells, we rapidly alternated excitation between two distinct cellular regions, each scanned for 70 µs. Volumes #1 and #2 delineate the boundaries of the two regions targeted by ULoVE during acquisition. *Bottom left*: Spatial and temporal characteristics of the ULoVE patterns. *Top right*: Fluorescence acquired during the ULoVE scans of volumes #1 (top) and #2 (bottom). Each column represents fluorescence intensity acquired during a 70-µs ULoVE scan. Each column represents the fluorescence acquired at 14 locations during the scan, with the top and bottom pixels representing fluorescence intensity at the beginning and end of the scan, respectively. The calculated position of the GEVI-labeled membrane during the scan is indicated by the white trace. *Bottom right*: classical ULoVE response amplitude trace, generated at each timepoint by summing the fluorescence intensity across the pixels of the corresponding column (scan) across both excitation volumes. (**F**) Cumulative distribution of the membrane shift. Colored lines are the values for individual cells (n = 12). Mean and s.d. are represented by the magenta thick line and shaded area, respectively. The dashed line indicates the 2.5-µm threshold as defined in panel **C**. (**G**) Comparison of subthreshold fluctuations in layer-2/3 neurons of the visual cortex expressing JEDI3sub-Kv acquired with ULoVE (black, n = 12 cells) or fast AOD-based fast raster imaging (gray, n = 5 cells), p = 0.56 and 0.64, respectively, for Kolmogorov Smirnov and two-sample Wilcoxon rank sum test (Supplementary Table 2). Arrowhead indicates the neuron shown in panel **B**. Source data

**Extended Data Fig. 4:**
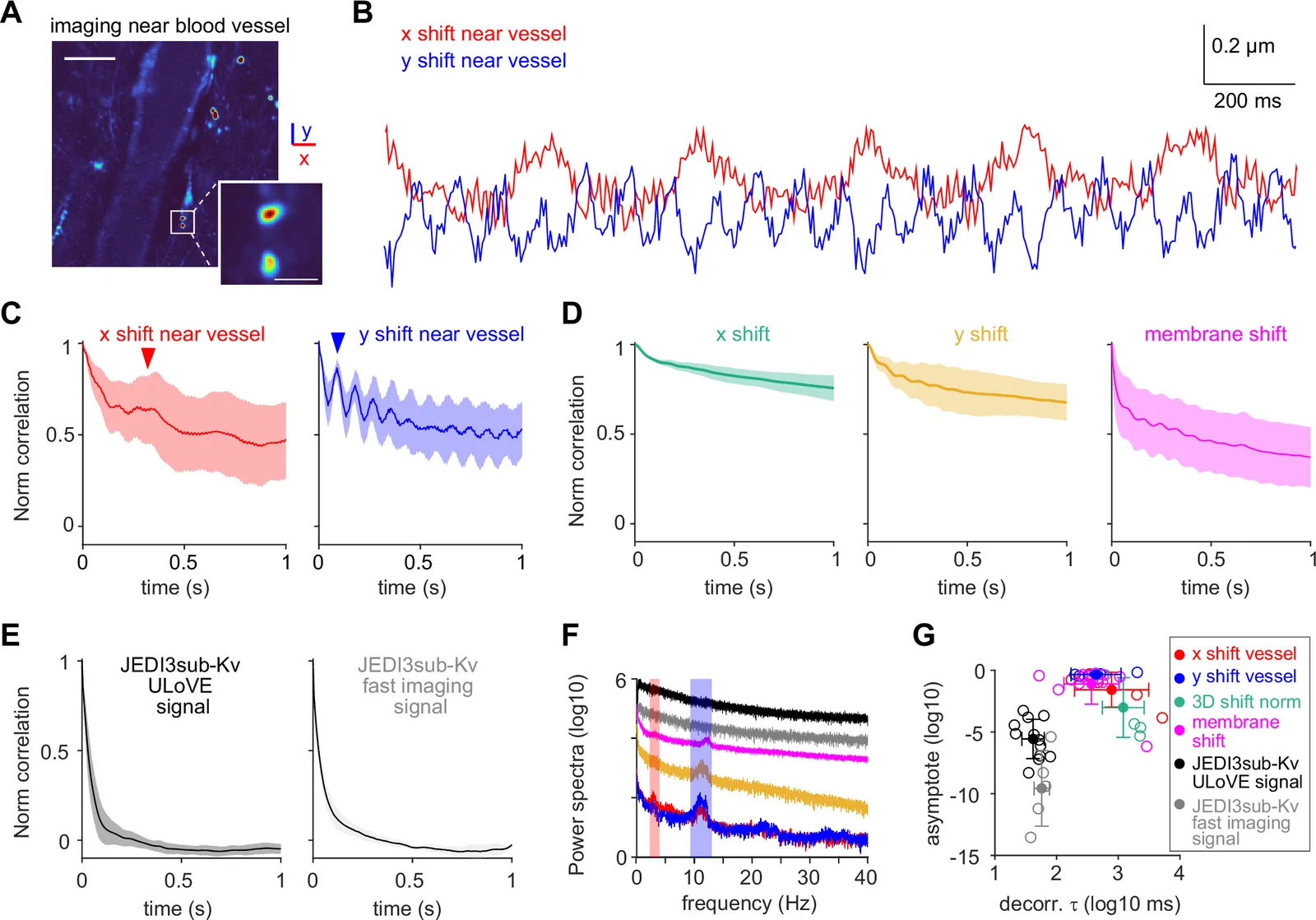
Distinguishing between genuine voltage signals and cardiovascular-related motion artefacts in JEDI3sub-Kv recordings obtained by fast imaging and ULoVE microscopy. (**A**) Image of a cortical blood vessel and bright, auto-fluorescent objects (zoomed-in). Scale bars are 20 and 2 µm (zoomed-in). (**B**) X (red) and y (blue) shifts of the auto-fluorescent objects rarely exceed 1 µm. Data was acquired at 200 Hz using AOD-based imaging. (**C**) Autocorrelations of the x and y shifts from panel **B**. Thick traces are the mean, and shaded areas are the s.d. n = 5 cells. Breathing and heartbeat frequencies are clearly visible in the x and y autocorrelations, respectively (2.58 Hz, red arrowhead; 10.64 Hz, blue arrowhead). (**D**) Autocorrelations of x and y shifts obtained from fast imaging (x, green and y, yellow, n = 5, data from Extended Data Fig. 3) and ULoVE recordings (magenta, n = 12, data from Fig. 2) of JEDI3sub-Kv-expressing neurons. Subtle heartbeat-related oscillations are visible in these autocorrelograms. (**E**) Autocorrelograms of JEDI3sub-Kv signals acquired by ULoVE (left, n = 12) and fast imaging (right, n = 5), display fast decorrelations with no discernable oscillatory components. (**F**) Power spectra between 0 and 40 Hz for signals from JEDI3sub-Kv ULoVE recording (black), JEDI3sub-Kv fast imaging (grey), membrane shifts obtained from ULoVE recording (magenta), y-shift obtained from fast imaging (yellow), and x (red) and y (blue) shifts from imaging autofluorescent objects near a blood vessel. Red and blue shaded columns represent the frequencies related to breathing and heartbeat, respectively, as identified in panel **C**. (**G**) Scatter plot of asymptotic levels as a function of the decorrelation time constants from the autocorrelogram. *Red and blue*: x and y shifts obtained from fast imaging of an object near a vessel, respectively (n = 5). *Green*: the Euclidean norm of the 3D shifts extracted from fast imaging of a JEDI3sub-Kv-expressing cell (n = 5). *Magenta*: the membrane shift obtained from ULoVE recordings of a JEDI3sub-Kv-expressing cell (n = 12). *Black*: JEDI3sub-Kv signals from ULoVE recording (n = 12). *Grey:* JEDI3sub-Kv signals from fast imaging (n = 5). Note that the axes are plotted on a log₁₀ scale to enhance visualization of the tight distribution of JEDI3sub-Kv signal data compared to the broader spread observed in the displacement traces. Open circles correspond to individual datapoints. Filled circles are the mean. Error bars are the s.d. Source data

Among the indicators, JEDI3sub-Kv demonstrated the highest overall improved performance in vivo, with a 1.7-fold greater subthreshold fluctuation range when compared with JEDI-2P-Kv (Fig. 2d), consistent with its larger response amplitudes to subthreshold changes in vitro (Fig. 1). We confirmed that the large fluorescence fluctuations we recorded reflected genuine GEVI subthreshold signals rather than motion artifacts (Supplementary Note 1, Extended Data Figs. 3 and 4 and Supplementary Videos 1 and 2). When compared with JEDI-2P-Kv, JEDI3sub-Kv also exhibited a 1.2-fold greater dynamic range—defined as the sum of the spike amplitude and the subthreshold fluctuation range (Fig. 2d). Spike amplitudes reported by JEDI3sub-Kv were 0.8-fold the response of JEDI-2P-Kv (Fig. 2d). This reduction in spike response amplitude was not observed in vitro, possibly due to multiple experimental parameter differences, including temperature, spike morphology and acquisition method. The dynamic range, subthreshold and spike responses of JEDI3hyp-Kv were not significantly different from those of JEDI3sub-Kv (Supplementary Table 2). Spike width was similar across all indicators (Fig. 2d).

Because photobleaching rates are highly dependent on illumination parameters^18,34^, we quantified photostability for each optical recording paradigm presented in this study. During ULoVE recordings, GEVIs exhibited a characteristic photobleaching profile, consisting of a rapid initial decay over a few seconds (Fig. 2e), followed by a prolonged, slower bleaching phase (Fig. 2f). The photobleaching rates were similar across the three indicators, although JEDI3 sensors showed slightly more pronounced bleaching during the initial seconds of illumination. We did not assess brightness differences in vivo to avoid confounding effects from variability in expression levels, which can arise due to differences in viral production methods, adeno-associated virus (AAV) injection quality, cranial window integrity and biological variability across cells and animals.

**Extended Data Fig. 5:**
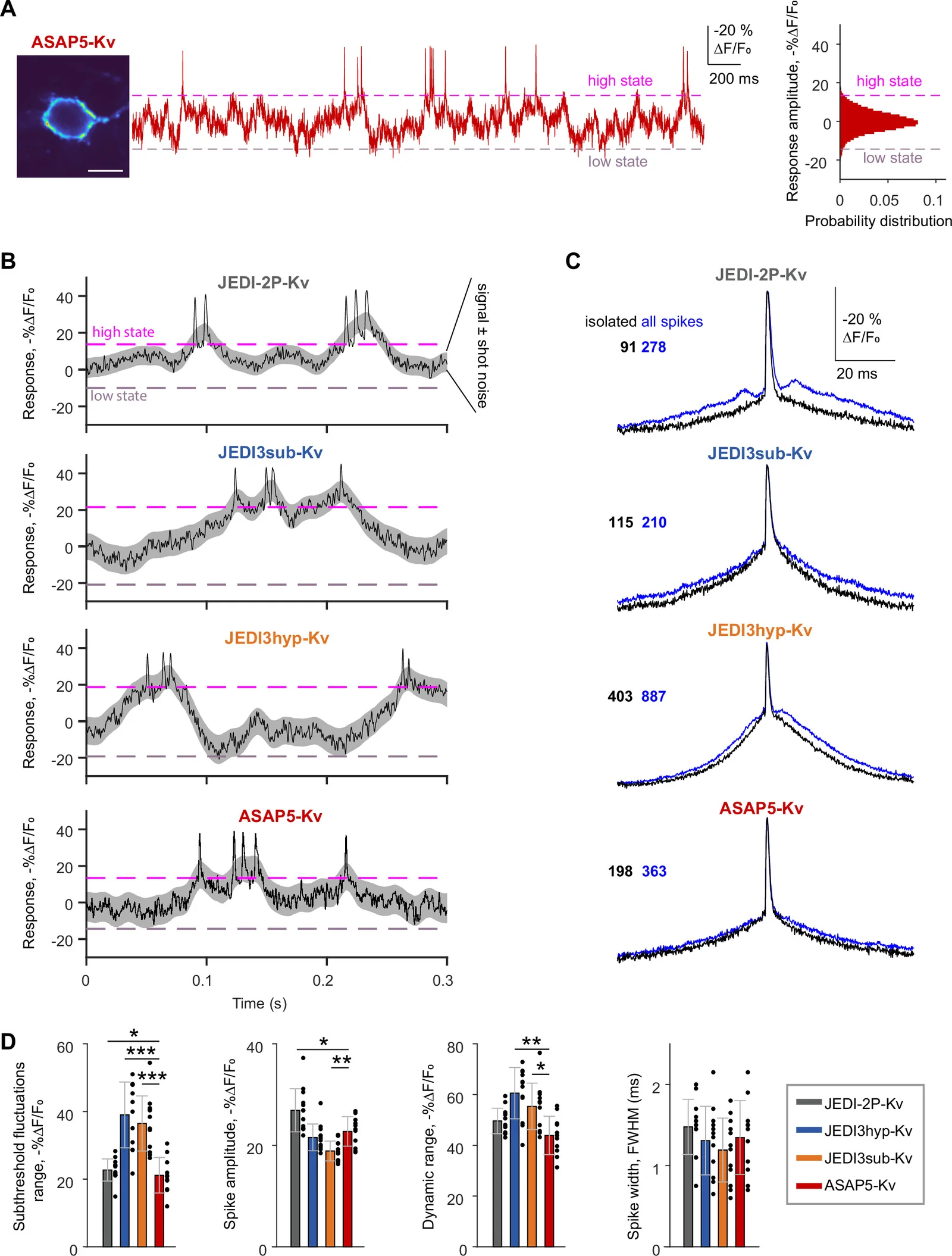
ASAP5 exhibits reduced responses to subthreshold fluctuations compared with JEDI3hyp and JEDI3sub. (**A**) Representative responses from a layer-2/3 neuron transduced using an AAV with Cre-dependent ASAP5-Kv expression. Scale bar, 10 µm. Magenta and pink dashed lines represent cut-offs for high and low states, respectively, of subthreshold fluctuations. Histogram (right) represents probability distributions of the low pass filtered signal traces. (**B**) Zoom of traces from Fig. 2b: grey shaded area indicates signal ± shot noise calculated as the local square root of the photon count. (**C**) Mean optical spikes obtained from isolated (black) or all spikes (blue). Values for isolated spikes for JEDI sensors are the same as in Fig. 2c. (**D**) Quantitative comparison between JEDI sensors and ASAP5-Kv. n = 13 (JEDI-2P-Kv), 12 (JEDI3sub-Kv), 12 (JEDI3hyp-Kv) and 11 (ASAP5-Kv). *, p < 0.05; **, p < 0.01; and ***, p < 0.001, two-sample Wilcoxon rank sum test (Supplementary Table 2). Error bars represent the s.d. ASAP5-Kv data are from Hao et al.^11^. Source data

Finally, we compared the performance of JEDI indicators to ASAP5 using equivalent experimental conditions, including microscopy method (ULoVE), acquisition parameters, brain structure and behavioral task (Extended Data Fig. 5). Consistent with in vitro results (Extended Data Fig. 1), both JEDI3sub-Kv and JEDI3hyp-Kv produced larger responses to subthreshold fluctuations than ASAP5-Kv, achieving 1.8-fold and 1.7-fold improvements, respectively. JEDI3sub-Kv displayed statistically identical spike responses as ASAP5-Kv, while JEDI3hyp-Kv produced 0.83-fold smaller responses. Spike widths for all indicators were statistically indistinguishable (Supplementary Table 2).

### Multi-cell subthreshold voltage imaging of JEDI3sub reveals correlated neural activity

**Fig. 3:**
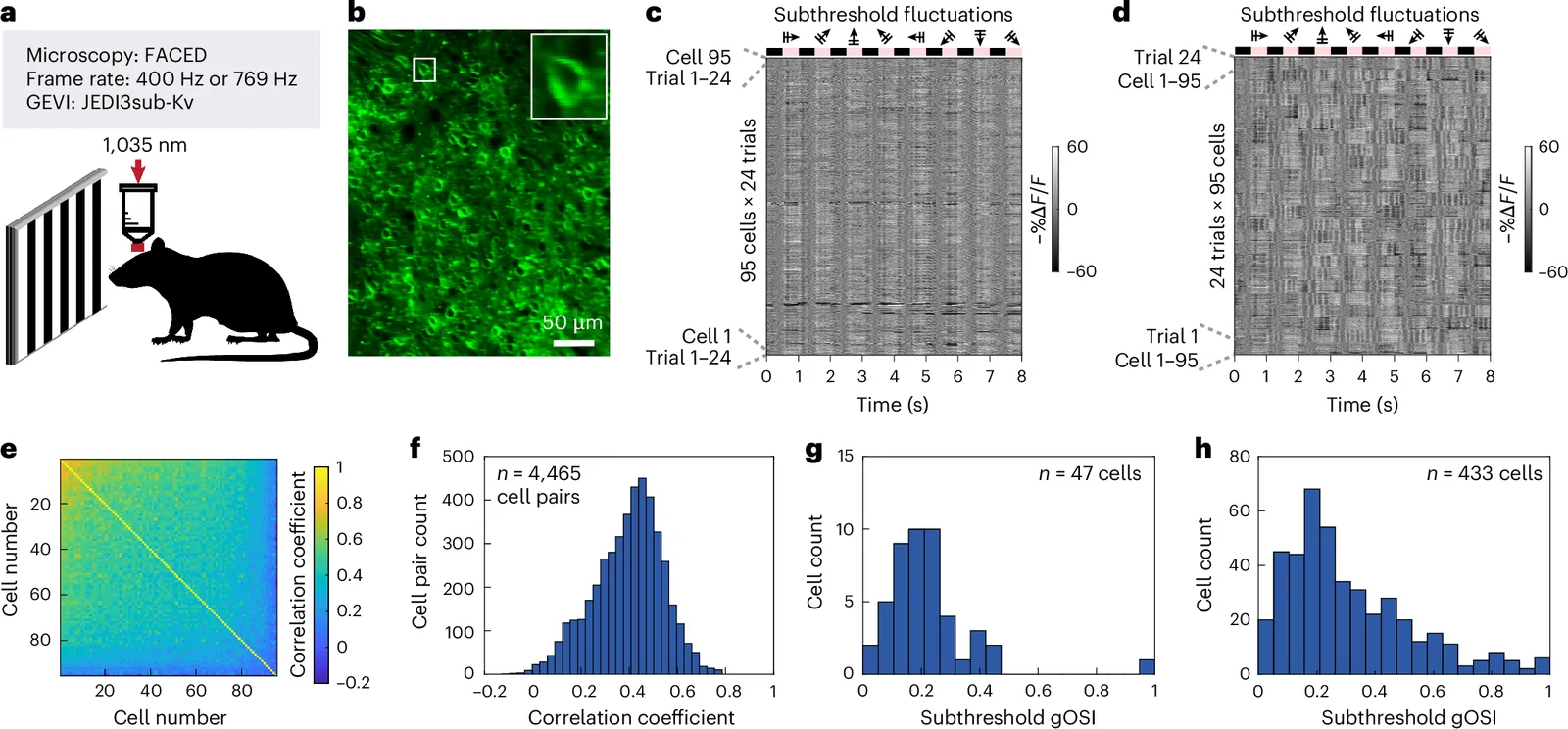
JEDI3sub simultaneously tracks subthreshold optical tuning in ~100 neurons in awake, behaving mice. **a**, Experimental design schematics. AAVs expressing the soma-restricted indicator JEDIsub-Kv from the pan-neuronal promoter hSyn were injected into the visual cortex. FACED microscopy was used to record 2P fluorescence responses to drifting gratings presented to the right eye of head-fixed, awake, behaving mice. The post-objective excitation power was set to 156 mW. Created in BioRender; St-Pierre, F. https://biorender.com/qvh2g4i (2025). **b**, 320 µm × 400 µm voltage imaging FOV showing JEDI3sub-Kv-expressing neurons. Representative of the five additional datasets shown in Supplementary Fig. 4. **c**,**d**, Single-trial subthreshold traces from 95 cells and 24 trials, grouped by cell (**c**) and trial (**d**). **e**, Correlation analysis of subthreshold responses. The plot displays the Pearson’s correlation coefficient matrix for subthreshold responses between 4,465 cell pairs, with cells sorted by their mean correlation coefficient. **f**, Histogram showing the distribution of correlation coefficients values. **g**,**h**, Neuron orientation-selectivity analysis. Histograms of subthreshold global orientation-selectivity index (gOSI) values for *n* = 47 orientation-selective neurons from one mouse (example shown in **b**) (**g**) and 433 orientation-selective neurons across 13 FOVs from two animals (**h**). Source data

**Extended Data Fig. 6:**
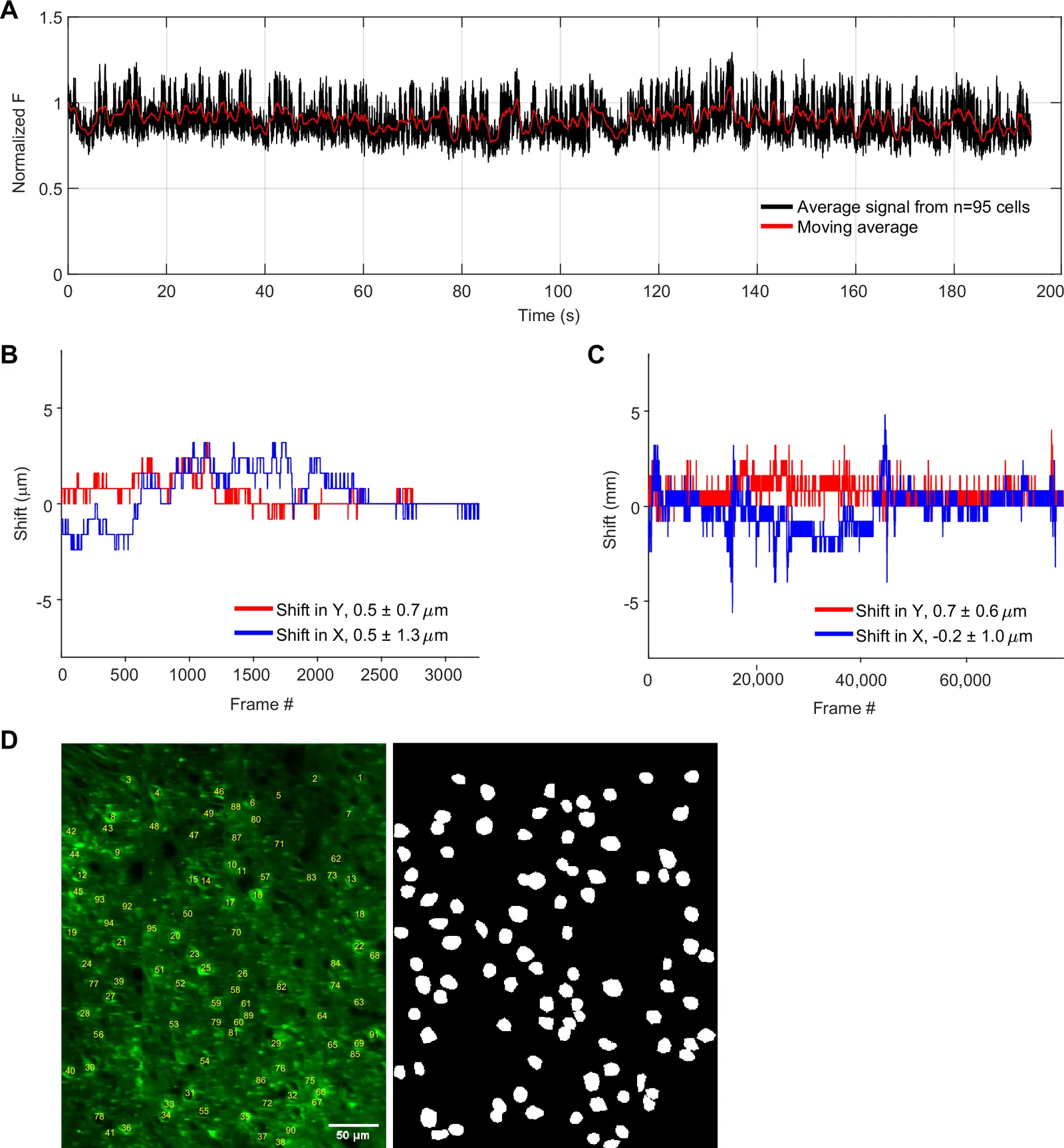
Photobleaching, lateral motion, and cell mask selection for the FOV in Fig. 3b. (**A**) Photobleaching of JEDI3sub over the ~200-s imaging session, as demonstrated by the mean fluorescence signal of 95 cells (black) and its moving average (red). Before averaging, each trace was normalized to its initial fluorescence value. (**B, C**) Image shifts caused by brain motion in one example trial (**B**) or across all trials (**C**). Boxed values are the mean ± s.d. (**D**) *Left:* 2P fluorescence image of L2/3 neurons with regions-of-interest (ROI) indices. *Right*: cell masks. Source data

We next sought to evaluate JEDI3 indicators to record subthreshold voltage dynamics across groups of cells. Soma-targeted JEDI3sub-Kv indicator was expressed in the visual cortex of awake mice (Fig. 3a), producing densely labeled populations of neurons with excellent indicator expression at the plasma membrane (Fig. 3b). We leveraged FACED 2P microscopy, which uses megahertz line scanning to enable the capture of voltage activity across large populations of neurons within the same focal plane^30,31^. Mice were presented with drifting grating visual stimuli in trials consisting of eight 0.5-s presentations, each featuring a distinct direction of motion. Each stimulus was separated by a 0.5-s dark period. Optical imaging was performed simultaneously at 400 Hz over fields of view measuring 320 × 400 µm. We did not apply photobleaching correction to the fluorescence traces because JEDI3sub exhibited minimal photobleaching during the aggregated ~200-s imaging sessions (Extended Data Fig. 6a; ratio of the final versus initial brightness: 0.92 ± 0.05, mean ± s.d., for 13 fields of view (FOVs) from two animals). Lateral motion artifacts, averaging <1 µm (Extended Data Fig. 6b,c), were effectively corrected through image registration (Supplementary Video 3).

Of 95 GEVI-expressing cells in a representative FOV (Fig. 3b and Extended Data Fig. 6d), over 88 cells (92%) displayed visually evoked subthreshold activity. This proportion is substantially higher than the 49% of layer 2/3 neurons showing drifting grating-evoked activity measured by calcium imaging^35^, which primarily reports suprathreshold rather than subthreshold activity. Visually evoked subthreshold activity can be observed from voltage traces of the same cells over 24 trials (Fig. 3c). Grouping traces of all 95 cells by trial, we observed biological variability, with each trial displaying distinct patterns of synchronized population activity—likely reflective of ongoing brain-state fluctuations (Fig. 3d; for all 24 trials, Supplementary Fig. 4a). We observed minimal out-of-plane sample motion (Supplementary Video 3), supporting the interpretation that these signals represent real GEVI responses reflecting membrane voltage dynamics.

Pairwise correlation analysis across all 4,465 unique cell pairs (95 × 94/2) yielded a mean correlation coefficient of 0.40 (Fig. 3e,f), highlighting substantial shared subthreshold fluctuations. Among the 88 neurons with visually evoked subthreshold responses, 47 (53.4%) demonstrated orientation selectivity. We assessed the degree of subthreshold orientation selectivity across this group of neurons using the global orientation-selectivity index (Fig. 3g).

We repeated imaging across five additional FOVs (122 ± 19 (mean ± s.d.) neurons per FOV, including the representative FOV above) in two mice, under identical experimental conditions. Like the initial FOV, these regions displayed trial-dependent, synchronized population activity (Supplementary Fig. 4b; for example, FOV 1–3). To assess the sensor performance at higher temporal resolution, we also acquired seven FOVs at an increased frame rate of 769 Hz over a smaller area (160 × 400 µm), capturing 71 ± 6 (mean ± s.d.) neurons per FOV (*n* = 2 mice with example FOVs shown in Supplementary Fig. 4b; FOV 4–5). Across all 13 FOVs imaged at either 400 Hz or 769 Hz, we recorded from 1,230 neurons, of which 1,023 (83.2%) displayed visually evoked subthreshold activity. Among these, 433 (42.3%) were classified as orientation selective. We plotted the distribution of global orientation-selectivity index values for orientation-selective neurons across all experiments to characterize population-level tuning heterogeneity (Fig. 3h).

These results demonstrate that JEDI3sub enables the tracking of visually evoked subthreshold activity in large neuronal populations—greater than 100 neurons simultaneously—providing a powerful platform for investigating multi-cell coding and the functional organization of the mouse visual cortex.

### JEDI3hyp tracks subthreshold dynamics associated with SPW-Rs in hippocampal interneurons

We investigated whether JEDI3hyp can capture behaviorally relevant subthreshold fluctuations using conventional 2P resonant-scanning microscopes. While these instruments offer limited FOVs at the high speeds required for voltage imaging, they remain widely used in neuroscience research. Validating JEDI3 indicators under these conditions is essential to assess their practicality and ensure compatibility with the imaging systems available in many neuroimaging labs.

First, we recorded sharp-wave ripples (SPW-Rs)—brief synchronous population events involved in cell sequence replay, which produce a stereotypical local field potential (LFP) waveform in the CA1 hippocampal region^36,37^. In awake mammals, SPW-Rs are accompanied by a marked increase in the firing rates of parvalbumin-expressing (PV^+^) interneurons that is phase-locked to the ripple oscillation^38^. PV^+^ interneuron spiking activity during SPW-Rs has been extensively studied using juxtacellular recordings and calcium imaging^39–41^. However, these methods do not report subthreshold voltage changes. In vivo patch clamping has been used to characterize subthreshold activity in pyramidal cells^42^, but it is difficult to perform on less abundant cell types such as interneurons. As a result, PV^+^ interneuron subthreshold activity related to SPW-Rs remains largely unexplored. Monitoring PV^+^ subthreshold potential changes may illuminate the activation of inhibitory inputs, which play an important role in SPW-R generation and termination^43,44^.

**Fig. 4:**
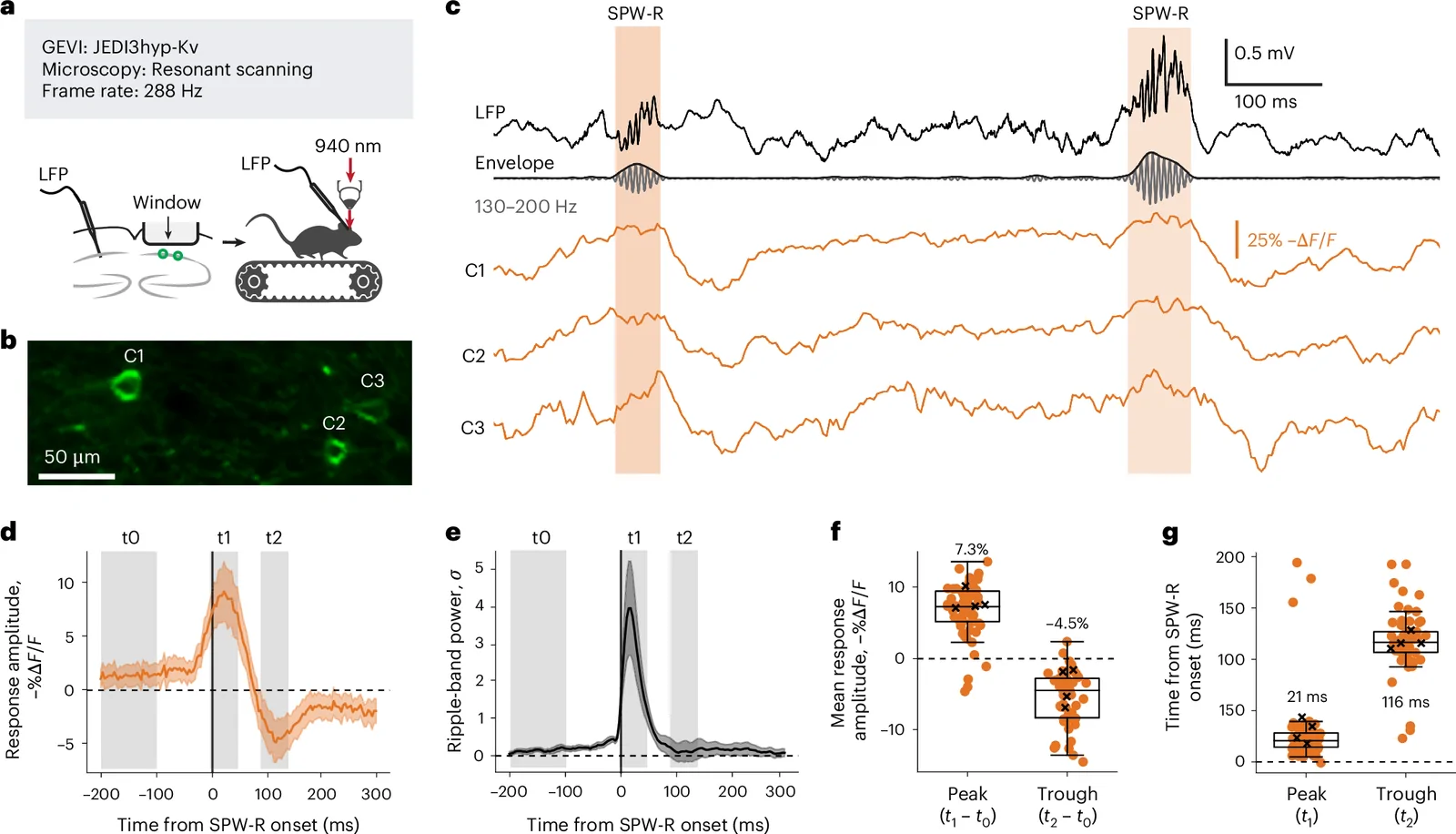
JEDI3hyp reveals subthreshold potential changes associated with spontaneous network oscillations in vivo*.* **a**, Experimental design schematics. PV-Cre mice (*n* = 4 females, postnatal day 53–67) were injected with AAVs expressing Cre-dependent soma-targeted JEDI3hyp-Kv in PV^+^ interneurons (green circles). Mice were head-fixed to a self-propelled treadmill, allowing the mice to run or rest freely. 2P resonant-scanning microscopy of the hippocampal CA1 region was conducted at a frame rate of 288 Hz using a laser set to 940 nm. LFPs were recorded simultaneously with a bipolar electrode. **b**, An example FOV in the CA1 pyramidal layer, representative of 24 regions imaged. **c**, Fluorescence and LFP traces during SPW-Rs. Segments of the fluorescence traces from the three representative cells shown in **b**, overlaid with the simultaneously recorded LFP. SPW-R events (orange bars) were detected as peaks in the envelope (black) of the ripple-band filtered trace (dark gray, 130–200 Hz). Fluorescence responses from the three cells from **b** (orange traces) show transient depolarizations during SPW-Rs and hyperpolarizations afterward. Fluorescence traces were processed for visualization using an exponentially weighted moving average with a 25-ms decay. Note that the 288-Hz frame rate supports a larger FOV but does not resolve individual spikes or ripple oscillations. **d**,**e**, Event-triggered PV^+^ interneuron fluorescence (**d**) and LFP ripple-band power (**e**) during SPW-Rs. Data are the mean ± s.e.m. The baseline (*t*_0_), peak (*t*_1_, 21 ± 25 ms) and trough (*t*_2_, 114 ± 25 ms) are marked with gray bars and used to calculate the responses in **f** and **g**. *n* = 60 cells and 4 mice. **f**,**g**, Mean fluorescence response (**f**) and timing from SPW-R onset (**g**) of the peak and trough in individual neurons (orange circles). Boxes show the median ± interquartile range; whiskers show the non-outlier range. Black crosses show averages by animal. Mean fluorescence increased during peaks (*P* = 0.00075) and decreased during troughs (*P* = 0.029; one-sided *t*-test, *n* = 4 animals). Source data

**Extended Data Fig. 7:**
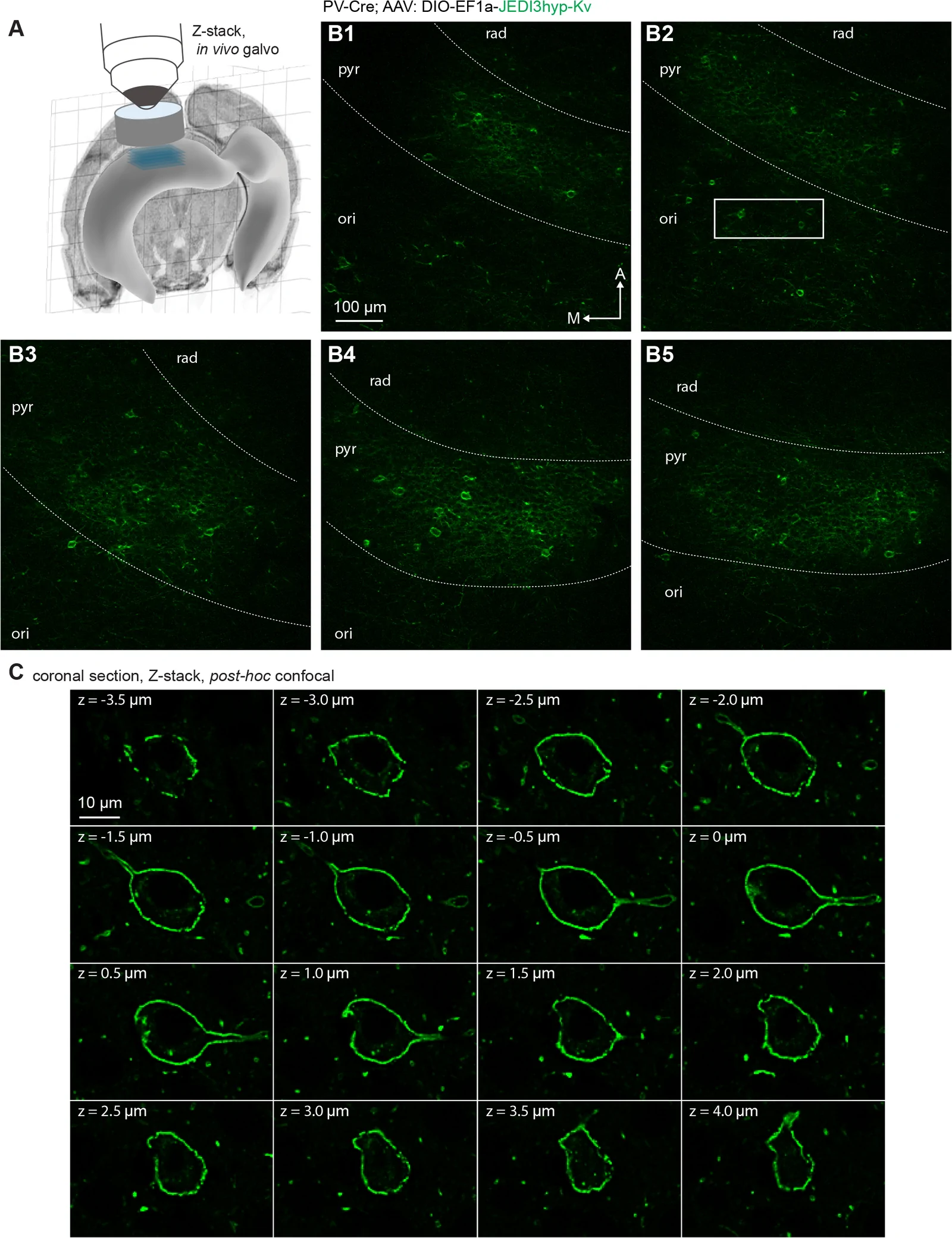
Representative expression pattern of JEDI3hyp in PV^+^ interneurons. (**A**) Schematic representation of the hippocampus and the CA1 region where a z-stack of images (blue) was captured. Image source: Allen Mouse Brain Atlas – Brain Explorer 2. (**B1-5**) Cropped regions from example optical sections are shown. Sections were separated by 30 µm in depth (Z). B2’s focal plane was adjusted by 10 µm to include the cells (c1-c3) shown in Fig. 4b (solid rectangle). Each slice was cropped at different lateral positions to follow the curvature of the hippocampus and the extent of labeling. Dashed lines show estimated layer borders. Arrow: M, medial; A, anterior. Layers: ori, oriens; pyr, pyramidal; rad, radiatum. (**C**) Confocal, deconvolved Z-stack of a representative JEDI3hyp-Kv-expressing cell from the same animal as in **B**. Imaging was conducted from a coronal brain section after perfusion. Optical sections are shown at 0.5 µm intervals. Representative of 4 mice.

While both JEDI3 variants were expected to perform similarly in this context, we chose JEDI3hyp due to its enhanced sensitivity to hyperpolarization (Fig. 1h). JEDI3hyp-Kv was transduced into the CA1 hippocampal region, expressed in PV^+^ interneurons using a PV-Cre mouse line (Fig. 4a). Conditional expression in PV^+^ interneurons resulted in sparse, predominantly membrane-associated labeling (Fig. 4b and Extended Data Fig. 7). JEDI3hyp-Kv was then imaged using a commercially available 2P resonant-scanning microscope in awake, behaving mice.

We set the acquisition frequency to 288 Hz, enabling the capture of graded membrane potential changes across a larger FOV, 250 × 90 µm (256 × 48 pixels). Spikes were not detected, as anticipated given our experimental design. The FOV was intentionally set large enough to capture multiple cells per trial, resulting in a frame duration of 3.5 ms. This time frame is substantially longer than the expected optical spike response from PV^+^ interneurons, which is likely under 1 ms, because PV^+^ cells have narrower spikes than the pyramidal neurons recorded in Fig. 2d (~0.3 ms^45^ versus ~0.7 ms^46–48^). Additionally, JEDI3hyp-Kv-positive pixels from each cell represented 1.3% ± 0.3% (mean ± s.d.) of the total FOV, further limiting the achievable SNR for detecting brief events such as action potentials.

JEDI3hyp-Kv tracked SPW-R-induced depolarizations and the following hyperpolarization of individual ripple-associated events in single neurons (Fig. 4c). We assessed whether the observed pattern of depolarization during SPW-Rs followed by hyperpolarization is a consistent feature across events, cells and animals. Averaging the responses of all recorded PV^+^ interneurons, we observed PV^+^ interneuron depolarization during SPW-Rs (Fig. 4d), consistent with the previously reported increase in PV^+^ interneuron firing rates^38,39^. These depolarizations likely reflect a mixture of subthreshold and suprathreshold activity, as the imaging temporal resolution will cause spike-related signals to be aliased. Peak depolarization occurred 21 ms after SPW-R onset, closely following the peak in ripple-band power at 17 ms (Fig. 4e–g). The average response of PV^+^ interneurons exhibited a post-SPW-R hyperpolarization, reaching a trough 116 ms after SPW-R onset. This post-SPW-R hyperpolarization mirrors a similar phenomenon reported in pyramidal cells, likely attributable to feedforward inhibition^42,49^.

The SPW-R-associated depolarization and subsequent hyperpolarization have also been observed using Voltron, a 1P-compatible voltage indicator combining a fluorescent dye and a voltage-sensitive protein^50^, corroborating our findings. Our results underscore the capability of JEDI3hyp-Kv to report behaviorally relevant voltage dynamics in vivo under 2P microscopy and leveraging a fully genetically encoded indicator.

### JEDI3hyp reports brain-state-dependent somatic and dendritic subthreshold voltage dynamics across cell types and cortical layers

We investigated whether JEDI3hyp could report subtle subthreshold voltage changes in dendrites and deep cortical layers. In quiet awake animals, small but consistent subthreshold voltage fluctuations in cortical neurons correlate with pupil dilations and constrictions^21,51–53^. These dynamics, which are thought to influence state-dependent sensory processing and behavior, remain poorly understood. The current standard measurement technique, whole-cell patch clamping, is technically demanding, has low throughput and is challenging in subcellular compartments and deeper cortical layers. We evaluated whether JEDI3hyp could overcome these limitations by enabling rapid, reproducible optical measurements of voltage dynamics across cortical layers, cell types and subcellular compartments.

**Fig. 5:**
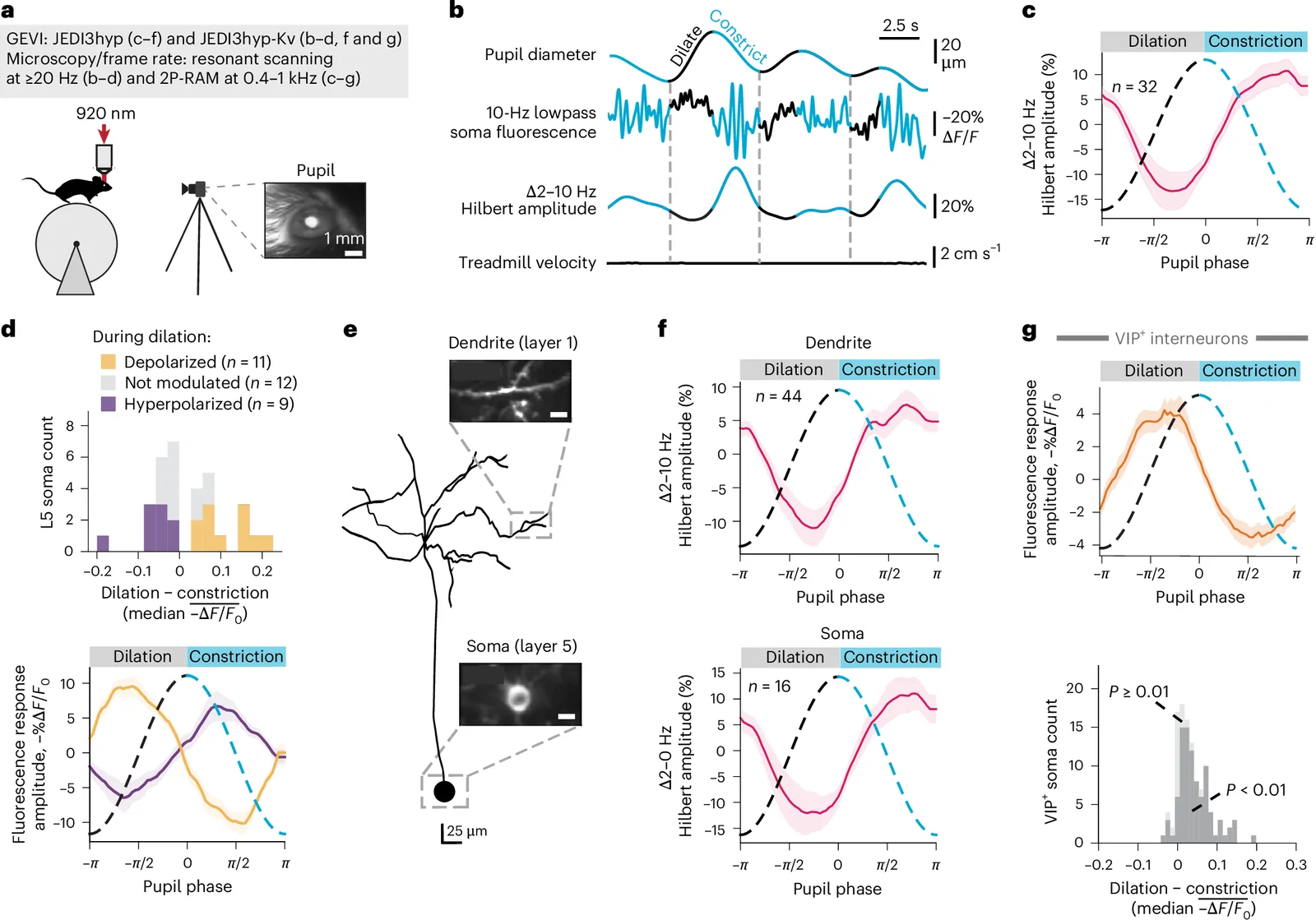
JEDI3hyp reports voltage changes associated with brain state in different cell types, subcellular locations and cortical layers. **a**, Experimental setup schematics. AAVs expressing Cre-dependent JEDI3hyp or JEDI3hyp-Kv were injected into the visual cortex of mice. Mice were head-fixed and freely allowed to move on a cylindrical treadmill. Experiments were conducted using 2P resonant-scanning (**b**–**d**) or random-access (**c**–**g**) microscopy using lasers set to 920 nm. Pupil diameter was simultaneously recorded. **b**, Example L5 IT-PN showing how changes in pupil diameter (top) are associated with changes in 2–10-Hz voltage oscillations, as reported by JEDI3hyp-Kv (2nd and 3rd traces). Dilation and constriction periods are colored black and blue, respectively. Vertical dashed lines separate adjacent dilation/constriction cycles. This example shows a period of quiet wakefulness, without any treadmill movement (bottom, solid black). **c**, Relationship between pupil phase and the relative amplitude of low-frequency activity in L5 IT-PNs. *n* = 32 somatic recordings from four mice. **d**, Top: histogram of L5 IT-PNs soma signals showing the difference in GEVI fluorescence between pupil dilation and constriction. Colored bars are *P* < 0.05. The Kruskal–Wallis test was performed between dilation and constriction periods per recording. Bottom: relationship between pupil phase and fluorescence response amplitude for L5 IT-PNs (mean ± s.e.m.). **e**, Example reconstruction of a JEDI3hyp-expressing L5 IT-PN neuron. An apical dendrite and its corresponding soma are shown. Scale bars, 20 µm. **f**, The relationship between low-frequency membrane potential oscillations and pupil phase was analyzed from dendritic (top, *n* = 44) and somatic (bottom, *n* = 16) recordings. Multi-depth imaging scans were acquired from four mice and achieved using a 2P-RAM mesoscope. **g**, Top: relationship between pupil phase and GEVI response amplitude in VIP^+^ interneurons (mean ± 95% CI, bootstrapped). Bottom: difference in the medians of period-wise mean GEVI responses between pupil dilation and constriction. For both panels, *n* = 111 somatic recordings from five mice. In the bottom panels, the dark-gray bars indicate the 92 (of 111) recordings that showed a significant difference in the per-period mean GEVI response between dilation and constriction periods (*P* < 0.01; Kruskal–Wallis test); of these 92 recordings, 86 showed greater median GEVI responses during dilation than during constriction. Source data

**Extended Data Fig. 8:**
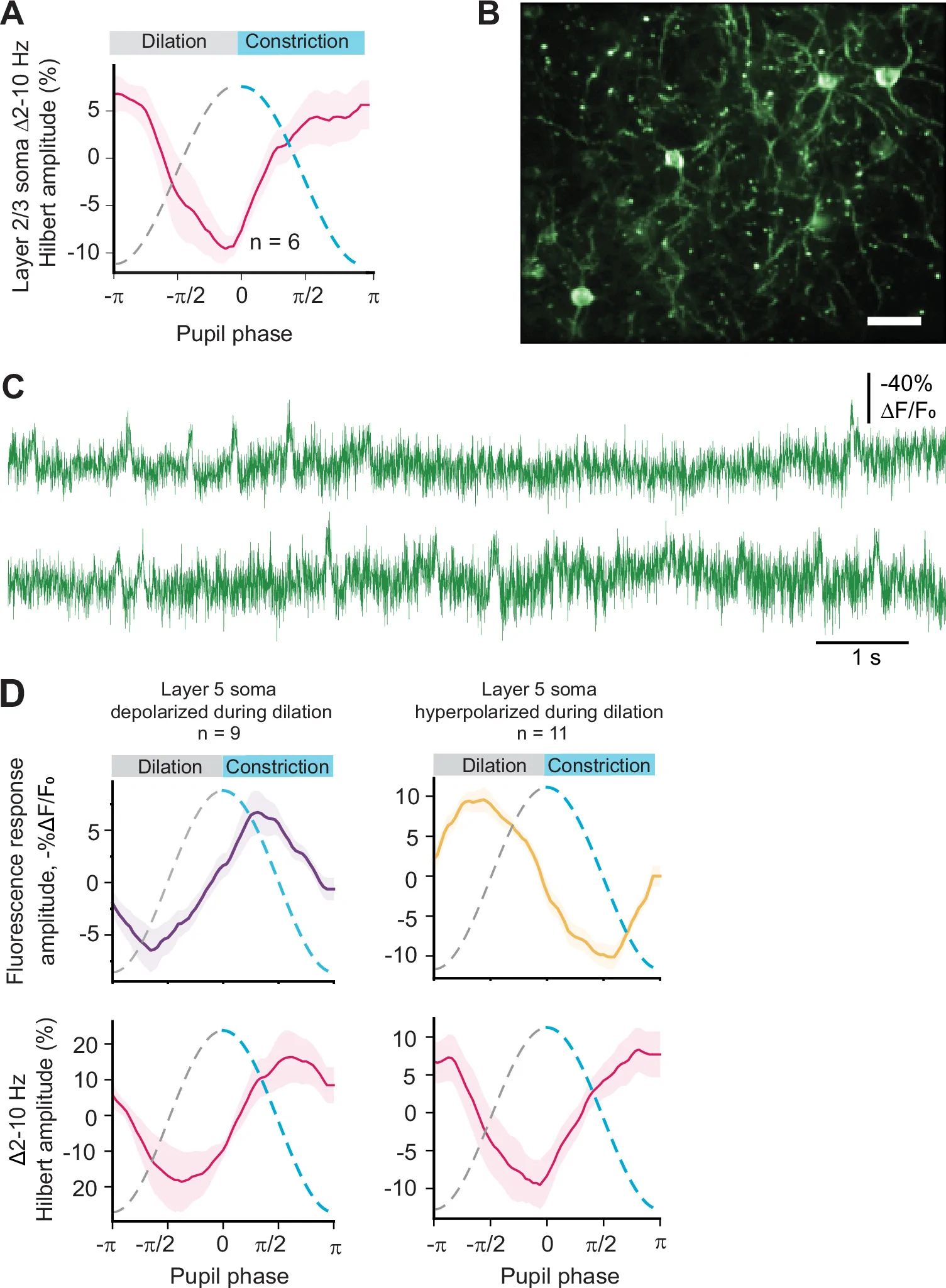
JEDI3hyp can report subthreshold changes across cortical layers. (**A**) Pupil dilation onset is associated with a reduction in low-frequency activity in layer-2/3 pyramidal neurons. n = 6 somatic recordings. (**B**) Representative resonant 2P image of JEDI3hyp expression in L5 IT-PNs, 434 µm below the surface of the brain. JEDI3hyp was not tagged with a soma-localized tag. Scale bar, 25 μm. (**C**) Representative JEDI3hyp responses from two layer-5 IT pyramidal neurons. (**D**) Layer 5 pyramidal neurons can be functionally categorized into two subclasses based on their response to pupil dilation: hyperpolarizing (purple, n = 9 somatic recordings) and depolarizing (yellow, n = 11 somatic recordings). While their overall ΔF/F₀ responses to pupil dilation and constriction differ (top panels), both subclasses exhibit similar changes in low-frequency activity as a function of pupil phase (bottom panels, red). Source data

Patch-clamp studies during quiet wakefulness have shown that pupil dilation reduces 2–10-Hz voltage oscillations in layer 2/3 pyramidal neurons, while constriction enhances them^51^. Using 2P voltage imaging with resonant-scanning microscopy, we observed that JEDI3hyp-Kv could optically report this activity pattern (Fig. 5a and Extended Data Fig. 8a) despite the underlying voltage changes averaging less than 2 mV^51^. Critically, we could detect subthreshold voltage dynamics in layer 5 intratelencephalic pyramidal neurons (IT-PNs), a population challenging to access with patch clamping due to their depth. Similarly to layer 2/3 neurons, layer 5 IT-PNs exhibited 2–10-Hz oscillations with lower Hilbert amplitude during pupil dilation and increased amplitude during pupil constriction (Fig. 5b,c and Extended Data Fig. 8b–d).

Approximately 63% of layer 5 IT-PNs displayed significant baseline fluorescence differences between pupil dilation and constriction. Of these, about half hyperpolarized, while the others depolarized during dilation (Fig. 5d, Extended Data Fig. 8d and Supplementary Table 2), suggesting two functional subclasses of neurons with differing responses to neuromodulators like acetylcholine and norepinephrine, which are strongly modulated during pupil dilation^54^.

In vivo patch clamping of dendrites is technically challenging, laborious, limited to larger-diameter dendrites and typically restricted to a single location per cell. We tested JEDI3hyp’s ability to overcome these challenges and visualize physiologically relevant voltage fluctuations in the apical dendrites of layer 5 neurons. Using a dual-recombinase approach^55^ for sparse JEDI3hyp expression, we matched layer 5 somas to their corresponding layer 1 dendrites. Because this application required recording away from the cell body, we used JEDI3hyp without the Kv soma-restriction motif.

**Extended Data Fig. 9:**
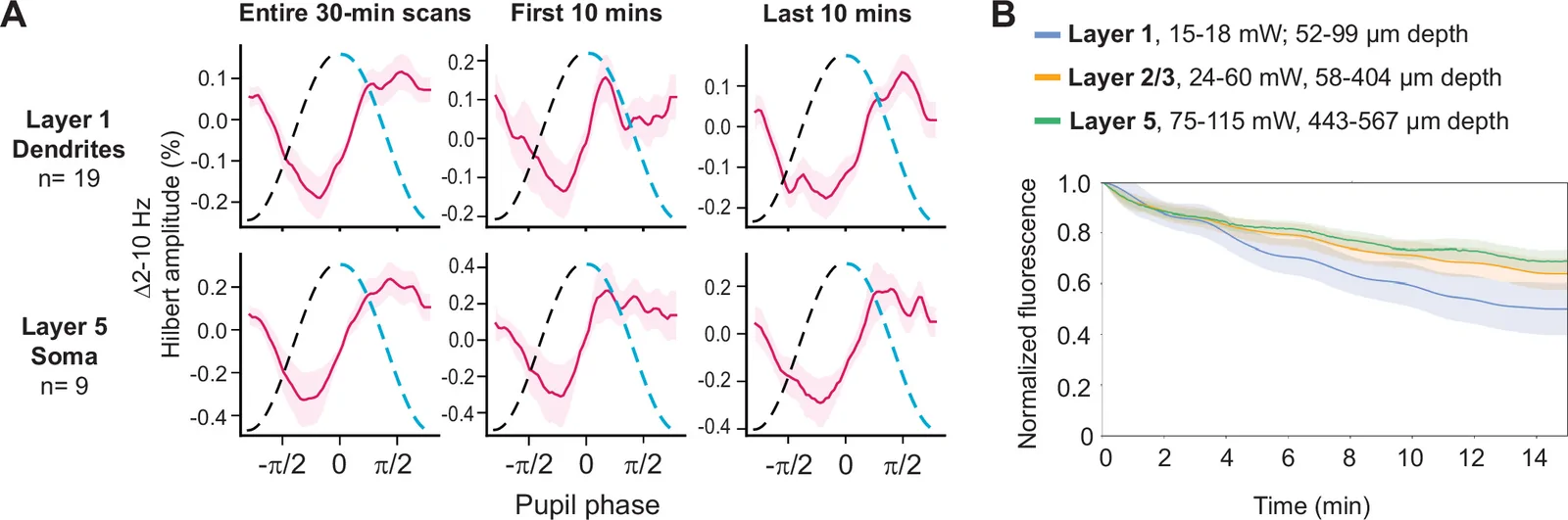
JEDI3hyp can report voltage signals over extended durations. (**A**) Changes in low-frequency activity in layer-5 somas (bottom panels) and corresponding dendrites (top panels) when analyzing the entire 30-minute scans (first column). Fluorescence changes associated with pupil phase remain stable, as shown by similar profiles between the first (center column) and last (third column) 10 minutes of imaging. Images were acquired using a 2P-RAM mesoscope. (**B**) JEDI3hyp demonstrates high photostability over 15 minutes of imaging. The power was measured post-objective. Layer 1: n = 5 somatic recordings from 2 mice; layer 2/3 n = 11 recordings from 2 mice; and L5 n = 8 somatic recordings from 4 mice. For all figures, lines with shaded regions are mean ± s.e.m. of the recorded cells. Data was acquired by 2P resonant scanning imaging of a combination of soma- and non-soma-localized JEDI3hyp. Source data

We conducted simultaneous multi-plane resonant-scanning recordings of layer 5 somas along with one to six of their corresponding apical dendrites. GEVI-expressing cells reported consistent voltage signals for 30 min of continuous illumination (Extended Data Fig. 9a), consistent with their measured photostability across varying imaging depths and laser intensities (Extended Data Fig. 9b). These recordings revealed comparable low-frequency subthreshold voltage dynamics during pupil dilation and constriction in dendrites and soma (Fig. 5e,f). These results demonstrated JEDI3hyp’s utility in visualizing brain-state-dependent dendritic signals over extended periods.

As expected, the large fluorescence fluctuations observed in JEDI3hyp-expressing dendrites were absent in nearby autofluorescent structures (Supplementary Fig. 5a). Furthermore, these control structures showed no correlation between 2–10-Hz fluorescence oscillations and pupil diameter (Supplementary Fig. 5b). Together, these findings support the interpretation that JEDI3hyp reports genuine voltage dynamics, rather than motion-related or noise-induced artifacts.

**Extended Data Fig. 10:**
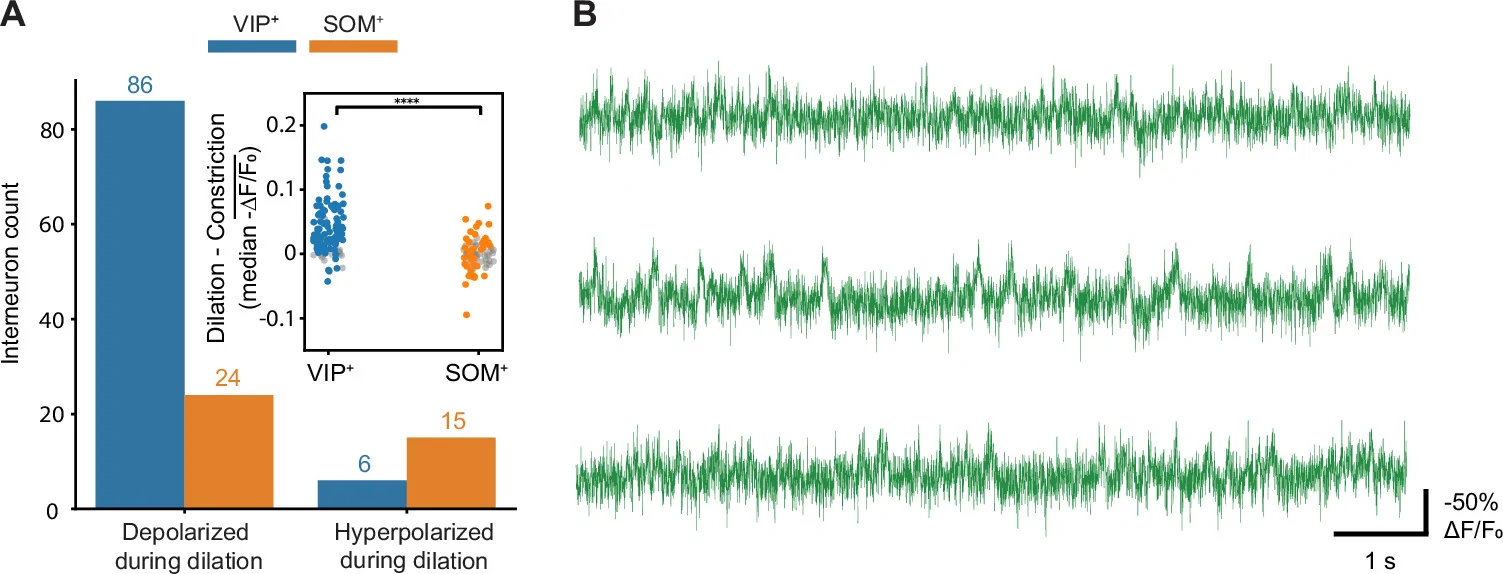
JEDI3hyp-Kv can report small subthreshold changes in interneurons. (**A**) Distribution of VIP^+^ and SOM^+^ cells exhibiting significant depolarization or hyperpolarization during constriction. Blue (VIP^+^) and orange (SOM^+^) bars (main plot) and dots (inset) indicate recordings in which the median ΔF/F₀ responses to dilation versus constriction differed significantly (p < 0.01; Kruskal-Wallis test; Supplementary Table 2). *Inset*: difference in median ΔF/F₀ between dilation and constriction epochs for individual recordings. Gray dots denote recordings in which the median responses during dilation and constriction did not differ significantly. **** p < 10^−11^; Kruskal-Wallis test, all recordings (gray and colored dots) were included: n = 111 VIP^+^ somatic recordings from 5 animals and 83 SOM^+^ somatic recordings from 2 animals. The same VIP^+^ dataset was used here and in Fig. 5g. (**B**) Three representative somatic recordings from JEDI3hyp-Kv-expressing VIP+ interneurons. Data was acquired using 2P random-access scanning with acoustic optic deflectors (Femto3D Atlas, Femtonics). Source data

GEVIs, such as JEDI3hyp, also facilitate cell-type-specific studies. We deployed JEDI3hyp-Kv to examine voltage changes in two GABAergic interneuron subtypes: vasoactive intestinal peptide-expressing (VIP^+^) and somatostatin-expressing (SOM^+^) cells. VIP^+^ interneurons mostly depolarized during pupil dilation and hyperpolarized during constriction, consistent with patch-clamp findings (Fig. 5g and Extended Data Fig. 10)^51^. SOM^+^ cells showed more variable responses (Extended Data Fig. 10a), reflecting their functional heterogeneity reported in electrophysiological studies^51^. These results demonstrate JEDI3hyp-Kv’s sensitivity, given that average pupil-associated voltage changes in VIP^+^ and SOM^+^ cells have been measured at ±1–3 mV^51^. More broadly, these results also establish JEDI3hyp as a robust tool for extended 2P imaging of brain-state-dependent subthreshold voltage changes across deep-layer somas, fine dendritic structures and diverse cell types.

## Discussion

In this study, we introduced JEDI3sub and JEDI3hyp, GEVIs optimized for enhanced subthreshold voltage detection with 2P microscopy. By addressing a critical gap in the 2P imaging toolbox, which has historically emphasized spike detection, these sensors enable a deeper understanding of neuronal dynamics across various spatial and temporal scales.

The GEVIs ASAP3 (ref. ^22^) and ASAP5 (ref. ^11^) were screened under 1P imaging conditions and exhibit reduced sensitivity under 2P excitation, as shown here and in a previous publication^18^. To overcome this limitation, we developed the JEDI3 sensors using a 2P screening platform, enabling the direct selection of variants optimized for 2P performance. Our platform was specifically adapted to enhance GEVI sensitivity to subthreshold voltage changes while preserving robust responses to suprathreshold depolarizations. This methodological innovation lays the groundwork for developing voltage indicators tailored to report distinct physiological voltage ranges.

Indicator mutations often influence multiple performance characteristics simultaneously, revealing critical trade-offs between key metrics. For example, inserting a single tryptophan into JEDI-2P produced the recent variant JEDI-2Psub, which enhanced subthreshold voltage detection but caused a dramatic ~50% reduction in brightness, slowed on-kinetics by 0.24 ms and substantially reduced suprathreshold responses^17^. To avoid these compromises, we considered and balanced multiple performance metrics during GEVI optimization.

The resulting two JEDI3 indicators exhibit comparable overall performance. However, we recommend JEDI3sub over JEDI3hyp for most applications due to its slightly higher brightness and enhanced spike-detection capabilities in certain comparisons. JEDI3hyp demonstrates larger responses to voltage steps below –80 mV (from a holding potential of –70 mV), particularly at elevated temperatures (≥33 °C). This property may make JEDI3hyp particularly useful for investigating voltage dynamics in astrocytes, mitochondria and plants—all of which can exhibit resting membrane potentials below –80 mV.

JEDI3 sensors demonstrated utility in detecting subthreshold voltage dynamics across different brain areas, cortical layers, cell types and subcellular compartments. Their combination with advanced imaging methods enabled the simultaneous monitoring of subthreshold activity in approximately 100 cells, substantially enhancing the scale of optical recordings. Even with conventional resonant-scanning microscopy, JEDI3 sensors outperformed the throughput of whole-cell patch clamping by enabling the rapid and simple recording of dendritic fragments and small groups of neurons. Subthreshold voltage dynamics unfold on slower timescales than action potentials, making their monitoring well suited to the more modest pixel acquisition rates of resonant-scanning systems.

Our experiments revealed previously uncharacterized functional subclasses within layer 5 IT-PNs interneurons, likely driven by differential responses to cortical neuromodulators such as acetylcholine and norepinephrine. These findings align with prior studies linking neuromodulatory transients to pupil dilation and the biphasic responses of layer 5 pyramidal neurons to cholinergic input^54,56–58^. These results demonstrate the value of JEDI3 sensors to uncover neuronal dynamics, opening avenues for understanding the roles of subthreshold activity in information processing. Of particular interest is their application for understanding dendritic activity, including the role of extended (‘plateau’) depolarizations in dendritic integration^6,59^.

Accurate monitoring of subthreshold voltage dynamics requires careful mitigation of mechanical artifacts, such as those caused by respiration, heartbeat and behavioral movements. To ensure that recorded fluorescence changes reflected genuine GEVI subthreshold fluctuations, we conducted extensive analyses of potential motion artifacts. We found negligible correlation between membrane displacement and fluorescence signals—likely because over 98.5% of motion events were smaller than 2.5 μm and could be effectively corrected using image registration or by exciting a volume larger than the average displacement, as implemented in ULoVE.

Temporal undersampling of action potentials can reduce their recorded amplitudes, potentially causing them to resemble subthreshold changes—a form of artifact known as aliasing. Increasing the acquisition rate can mitigate these artifacts by improving temporal resolution, at the cost of fewer recorded cells or a lower SNR. In the visual cortex, cell density is moderate, and membrane overlap between neurons is relatively limited. In contrast, brain regions with higher cell body density, such as hippocampal CA1, pose greater challenges for spatial resolution. In such cases, overlapping membranes may lead to signal loss (if overlapping pixels are excluded) or signal contamination (if retained), although demixing algorithms may help alleviate this issue. Alternatively, strategies to provide sparse GEVI expression can reduce overlap, but this comes at the cost of recording fewer neurons simultaneously.

Dense populations of GEVI-expressing neurons generate substantial neuropil background fluorescence, which can obscure fluorescence decreases during depolarization. Reversing response polarity can improve spike detection by enhancing contrast against baseline fluorescence^60^. However, such ‘positive-going’ indicators often exhibit lower baseline brightness, which can hinder the detection of hyperpolarizations and small depolarizations. These inherent compromises highlight the need for a diverse repertoire of GEVIs tailored to specific experimental demands.

While JEDI3 indicators have enhanced the sensitivity of subthreshold voltage detection, further advancements in response amplitude and brightness would enable robust voltage recordings across greater numbers of cells, smaller structures and finer voltage changes. Faster on-kinetics would improve the detection of narrow action potentials, such as those generated by fast-spiking PV^+^ interneurons.

Overall, JEDI3 sensors substantially expand the 2P imaging toolbox, enabling sensitive detection of subthreshold voltage dynamics. When combined with emerging imaging technologies and optogenetic strategies, these sensors offer powerful opportunities to dissect the complex interplay between subthreshold membrane potential fluctuations, neurotransmitter signaling and circuit-level computations.

## Methods

Supplementary Table 1 lists experimental conditions used to generate the data in the main figures. Supplementary Table 2 provides detailed information on the statistical comparisons performed for the main and supplementary figures.

### High-throughput GEVI screening

#### Predicted GEVI 3D structures and directed evolution lineage tree

Predicted three-dimensional (3D) models of the JEDI3 sensors were generated by introducing targeted in silico mutations into the previously modeled structure of ASAP2s^18^, using the mutagenesis wizard of the PyMOL Molecular Graphics System (v.3.0, Schrödinger). The evolutionary tree of mutations was rendered using Microsoft Office Visio.

#### Media and solutions

**Growth medium 1** comprises high-glucose Dulbecco’s Modified Eagle Medium (Sigma-Aldrich, D1145-500ML) supplemented with 10% fetal bovine serum (Sigma-Aldrich, F2442-500ML), 2 mM glutamine, 100 units per ml penicillin, 100 μg ml^−1^ streptomycin and 750 μg ml^−1^ of the antibiotic G418 sulfate (geneticin).

**Growth medium 2** comprises high-glucose Dulbecco’s Modified Eagle Medium supplemented with 5% fetal bovine serum, 2 mM glutamine, 100 U ml^−1^ penicillin and 100 μg ml^−1^ streptomycin.

**Imaging solution 1** comprises 110 mM NaCl, 26 mM sucrose, 23 mM glucose, 20 mM HEPES, 5 mM KCl, 2.5 mM CaCl_2_·2H_2_O, 1.3 mM MgSO_4_, adjusted to pH 7.4 with NaOH.

**Imaging solution 2** comprises 106.5 mM NaCl, 26 mM sucrose, 23 mM glucose, 20 mM HEPES, 8.5 mM KCl, 2.5 mM CaCl_2_·2H_2_O, 1.3 mM MgSO_4_, adjusted to pH 7.4 with NaOH.

#### Plasmid construction

GEVI libraries were generated by site-directed saturation mutagenesis targeting single residues. To obtain a more uniform distribution of residues for saturation mutagenesis, we combined primers containing the NNT, VAA, ATG or TGG codons (N = any base; V = A, G, or C) at a molar ratio of 16:3:1:1. All primers were synthesized by Millipore Sigma. The 20-μl PCR reaction mix contained 1 μl of 10-μM forward primer mix, 1 μl of 10-μM reverse primer, 5 ng of template plasmid and 10 μl of 2× PCR master premix (PrimeSTAR HS DNA polymerase, Takara). DNA was amplified using the following protocol: an initial denaturation step at 98 °C for 30 s; 30 amplification cycles consisting of 98 °C for 10 s, 57 °C for 10 s and 72 °C for 1 min per kilobase of fragment length; and a final extension step at 72 °C for 5 min. The pcDNA3.1/Puro-CAG^61^ backbone was linearized using the restriction enzymes NheI and HindIII. PCR products and linearized backbones were purified using gel electrophoresis and the GeneJET Gel Extraction Kit (Thermo Fisher Scientific). PCR products, including a C-terminal GSSGSSGSS-linked^62^ mBeRFP^63^ reference protein, were assembled in the vector backbone using the In-Fusion assembly system (In-fusion HD Cloning Plus, Takara) according to the manufacturer’s instructions. The mBeRFP CDS used for amplification had an Glu6Phe mutation compared with the original sequence; this variant of mBeRFP was used for all cloning of mBeRFP. The In-Fusion reaction mix was transformed into commercial chemically competent bacteria (XL10-Gold, Agilent) with a transformation efficiency exceeding 5 × 10^9^ colony forming units per μg of DNA. Liquid cultures were inoculated with manually picked colonies, and purified plasmids were prepared using a 96-well plasmid purification kit (96-well Mini Plus Plasmid Extraction System, Viogene, GF961001) following the manufacturer’s instructions.

Plasmids used for in vitro characterization were assembled using the In-Fusion cloning technique (Takara Bio) into a pcDNA3.1/Puro-CAG vector with or without a C-terminal GSSGSSGSS-linked mBeRFP reference protein. Liquid cultures were inoculated with manually picked colonies, and purified plasmids were prepared using a purification kit (Mini Plus Plasmid Extraction System, Viogene number GF2002) following the manufacturer’s instructions. The plasmid sequence was confirmed using whole-plasmid sequencing based on nanopore technology (Plasmidsaurus) or by Sanger sequencing (Eurofins Genomics). The ASAP5 sequence was amplified from the pAAV-hsyn-ASAP5-WPRE plasmid (Addgene, plasmid number 225709). The pcDNA3.1/Puro-CAG-EGFP plasmid was generated in our lab previously^18^.

#### Cell culture and transfection in 96-well plates

To screen GEVIs for responses within the physiological range, we used HEK293 cells stably expressing the human Kir2.1 channel^64^, which maintain a resting membrane potential of approximately −83 mV under our imaging conditions. HEK293-Kir2.1 cells were cultured at 37 °C with 5% CO_2_ in growth medium number 1. G418 was added to maintain the expression of the Kir2.1 transgene, which was chromosomally integrated with a G418 resistance gene.

For screening GEVIs, glass-bottom 96-well plates (P96-1.5H-N, Cellvis) were first coated with poly-D-lysine (30−70 kDa) to promote cell adherence to the glass. The coating procedure was performed for 1 h at room temperature or 37 °C, and the plates were washed twice with Dulbecco’s Phosphate Buffered Saline (Corning, 21-031-CV). HEK293-Kir2.1 cells were then plated to 60–80% confluency in 100 μl of growth medium number 2.

We randomly selected 88 variants per saturation mutagenesis library. According to statistical modeling^65^, our library generation and sampling strategy produced a ∼99% theoretical probability that any given screened library included the best residue. Each well was transfected with jetPrime (Polyplus) according to the manufacturer’s protocol, using the following specifications: a mixture of 130 ng DNA, 0.4 μl jetPRIME transfection reagent and 10 μl jetPRIME buffer was mixed with 50 μl of growth medium number 2, and then added to the well. Independent transfections were defined as transfections in which DNA was added separately to each well. After 4 h or the next day, 100 μl growth medium number 2 from each well was replaced with fresh growth medium number 2 to minimize potential cytotoxicity from the transfection reagents. Two days after transfection, the cells were washed twice with 150 μl of imaging solution number 1 at room temperature. Wells were filled with 100 μl of the imaging solution number 1 and screened with our 2P high-throughput screening platform (see below). The imaging solution number 1 was adjusted with H_2_O to have a final osmolarity between 290 mOsm and 310 mOsm per kg of water.

#### 2P GEVI screening system

2P library screening was completed using roughly the same hardware as previously described^18^. An inverted microscope with multiphoton capability (A1R-MP, Nikon Instruments) was used for 2P screening and in vitro characterization of GEVIs. The 2P excitation light was generated by a titanium:sapphire femtosecond laser (Chameleon Ultra II, Coherent) with a repetition rate of 80 MHz and a tuning range of 680 nm to 1,080 nm. Laser power was set using an acousto-optic modulator and delivered to the sample plane through a 20× 0.75-NA objective (CFI Plan Apochromat Lambda, Nikon Instruments). The emission light from the sample was split using a 560-nm dichroic mirror (348958, Chroma) and filtered by 525/50-nm (353716, Chroma) and 605/70-nm (Nikon Instruments) filters for green and red channels, respectively. Emitted photons were detected by gallium arsenide phosphide (GaAsP) photomultiplier tubes (PMTs). A motorized travel stage (H139E1, Prior or SCANplus IM 130× 85 - 2 mm, Märzhäuser Wetzlar) was used to control the position of the FOV, hold 96-well plates during screening and hold the perfusion chamber during electrophysiological characterization.

To support automation of the system, data acquisition and output boards (PCI-6229 and PCI-6723, National Instruments) were connected to the microscope computer through a PXI Chassis (PXI-1033, National Instruments). The computer was equipped with an Intel Xeon Gold 6334 Processor, 8-core, 3.60 GHz, 256 GB of DDR4 RAM and two 12 TB 7200 RPM hard drives configured in RAID 0 to facilitate high-speed imaging. JOBS scripts in NIS-Elements HC (v.5.42.07, Nikon Instruments) were used to control the microscope system (for example, stage position), manage the optical configurations (for example, excitations), initiate image acquisition and trigger the stimulator.

A digital isolated high-power stimulator (4100, A-M System) was used to provide electric field stimulation. Electric pulses were passed through a pair of electrodes made from 0.5-mm-wide platinum wires (99.95% pure, AA10286BU, Fisher Scientific). The two L-shaped electrodes, each 2 mm in horizontal length and 3 mm apart, were secured on a 3D-printed polylactic acid holder. The holder was fixed to a motorized linear translation stage (LSM050B-E03T4A-MC10T3, Zaber), which was used to raise and lower the electrodes in and out of individual wells. Two smaller manual linear translation stages (411-05S, Newport) were used to fine-tune the *x*–*y* position of the electrodes in the plane parallel to the screening plate. During electric field stimulation, the electrodes were submerged under the imaging solution number 1, with the centers of the electrode wires 500–600 μm above the bottom of the well.

#### 2P GEVI screening

For each well of the screening plate, four nonoverlapping FOVs of 512 × 32 pixels were imaged at a rate of 440 Hz using a resonant galvanometer scanner. The laser was tuned to 940 nm and set to 75 mW at the sample plane. To collect images for the brightness calculation, 50 frames of the GEVI and the reference protein mBeRFP were collected in parallel on the green and red channels, respectively. To the same FOV, electric field stimulation was then applied during continuous collection of the green channel for 4,000 frames (approximately 9 s).

All stimulation trains were generated in AMS4100-1 software (v.1.1, A-M Systems) and uploaded to the stimulator as a series of monophasic square pulses. The protocol was input as five pulses of 60 V for 1 ms, with an inter-pulse period of 300 ms. These five stimulations were then followed 1.3 s later by a ramp with the sequence: 15 V for 1 ms, 25 V for 1 ms, 30 V for 1.5 ms, 30 V for 2.5 ms, 30 V for 3.5 ms, 30 V for 4.5 ms, and 30 V for 7.5 ms, with an inter-pulse interval of 300 ms. The ramp was followed by a 100-Hz train consisting of ten pulses at 30 V for 2.5 ms. The stimulation protocol concluded 1.05 s later with an additional five pulses at 60 V for 1 ms, with an inter-pulse interval of 300 ms.

#### Analysis of high-throughput screening data

Image analyses were performed by custom routines in MATLAB (v.R2024a, MathWorks) as previously described^18^. Time-lapse images recorded in ND2 format were imported into MATLAB using the Bio-Formats toolbox (v.6.3.1)^66^. For both channels (red and green) of each FOV, saturated pixels from any point during the videos (for example, from overexpressing cells) were removed. Background correction was computed by subtracting the mode intensity of the first frame’s pixel intensity histogram from each non-saturated pixel’s value. An initial foreground mask was computed from the first 20 frames of each channel by applying predefined intensity thresholds for the green and red channels to distinguish pixels containing GEVI and reference protein signal from pixels just containing autofluorescence noise. Relative GEVI brightness ($B=\frac{{F}_{{\rm{GEVI}}}}{{F}_{{\rm{mBeRFP}}}}$, arbitrary units) was calculated using the mean fluorescence intensity of the first 20 frames in the green channel (${F}_{{\rm{GEVI}}}$), divided by the average fluorescence intensity of the first 20 frames in the red channel (${F}_{{\rm{mBeRFP}}}$). Relative GEVI brightness is reported to correct for FOV-to-FOV differences in the number of transfected cells, the number of selected pixels, and overall expression (for example, due to pipetting errors or biological variation).

Quantification of response amplitudes from the 9-s recording could be distorted by the inclusion of non-plasma-membrane-localized GEVIs within a FOV that were bright but nonresponsive. To account for nonresponsive pixels, we applied an additional correlation-based selection step. Specifically, a template was created from the mean temporal trace of all remaining pixels in the recording. The temporal traces of each remaining pixel were correlated with the template using Pearson correlation coefficients. Pixels were ranked according to their correlation coefficients. To determine the optimal pixels, we evaluated the SNR across cumulative groups of pixels and identified the group corresponding to the peak SNR value. This subset was then used as the final output of the correlation filtering process. FOVs with final masks containing fewer than 300 pixels were removed from further analysis as reduced pixel selection often is representative of a low cell confluency, poor transfection or a detrimental mutation. Using the pixels from the final mask, we calculated the total signal at each time point to measure fluorescence over time. We corrected the green (GEVI) fluorescence trace for photobleaching by dividing each trace by the best-fit three-term exponential on the mean fluorescence using data outside stimulations. To quantify photostability, (P, arbitrary units), the area under the curve (AUC) was calculated from the fluorescence-versus-time trace normalized to 1 at t=0. The peak response amplitude (*R*) was quantified from the photobleaching-corrected fluorescence trace ($R=\frac{F}{{F}_{0}}-\,{{\rm{s}}.{\rm{d}}.(F}_{{\rm{pre}}})$, arbitrary units), where F is the peak fluorescence, ${F}_{0}$ is the mean baseline fluorescence for the first 100 ms of the recording, and ${F}_{{\rm{pre}}}$ is the baseline fluorescence for the 100 ms preceding the pulse. The standard deviation of ${F}_{{\rm{pre}}}$ was subtracted from $\frac{F}{{F}_{0}}$ (response) to account for baseline fluctuations. Given that FOVs can vary in the number of pixels selected, the FOV area-weighted mean was taken for each well when determining the average response.

Compound metrics that consider multiple performance metrics were used to simplify the ranking of indicators^18^. We previously developed the detectability index metric (${D}_{{\rm{I}}}=R\sqrt{B}$, arbitrary units), where R is the peak response amplitude and B is the relative brightness. We also sought to consider the impact of photobleaching on voltage recording and avoid variants that are bright but bleach rapidly. Thus, we used the detectability budget (${D}_{{\rm{B}}}={D}_{{\rm{I}}}\sqrt{P}$, arbitrary units), which combines all measured performance characteristics by replacing the initial relative brightness in the *D*_I_ equation with the average relative brightness measured during the screening experiment. None of the above metrics include physical units, as they are relative parameters intended solely for comparative ranking of indicators during the screening process.

### GEVI characterization in vitro

#### Whole-cell voltage clamp setup

HEK293A cells (Thermo Fisher Scientific, R705-07; RRID: CVCL_6910) were plated on 30–70-kDa poly-D-lysine-coated circular cover glass (12 mm, number 0, 633009, Carolina) at 30% confluence in growth medium number 2, 2 days before imaging. Chemical transfection was performed on the same day as plating, using 100 ng of DNA and 0.3 μl of FuGENE HD (Promega Corporation) transfection reagent per well of a 24-well plate (Costar, 3524), following the manufacturer’s instructions. Unless indicated otherwise, pcDNA3.1/Puro-CAG plasmids expressing the GEVI with no reference protein were used. The cells were cultured at 37 °C with 5% CO_2_ before and after transfection. The next day, the transfection medium was replaced with fresh growth medium number 2 to minimize potential cytotoxicity from the transfection reagent.

Glass micropipettes (1B150-F-4, World Precision Instruments) were prepared using a pipette puller (P1000, Sutter), achieving a tip resistance of 2–6 MΩ. Micropipettes were loaded with internal solution composed of 115 mM K-gluconate, 10 mM HEPES, 10 mM EGTA, 10 mM glucose, 8 mM KCl, 5 mM MgCl_2_, 1 mM CaCl_2_, adjusted to pH 7.4 with KOH. The internal solution was adjusted with H_2_O to be 10 mOsm per kg lower than the imaging solution number 1, which typically resulted in an internal solution between 280 mOsm and 300 mOsm per kg. The micropipette was mounted on a patch-clamp headstage (CV-7B, Molecular Devices) and positioned using a micromanipulator (SMX series, Sensapex or a Mini23, Luigs & Neumann). The coverslip seeded with the transfected cells was placed in a custom glass-bottom chamber, designed based on the Chamlide EC (Live Cell Instrument), with the bottom of the chamber made from a 24 × 24-mm number 1 glass coverslip (Thermo Scientific). Cells were continuously perfused with an imaging solution number 1 at approximately 4 ml min^−1^ using a peristaltic pump (505DU, Watson Marlow). Whole-cell voltage clamp was achieved using a MultiClamp 700B amplifier (Molecular Devices). Patch-clamp data were recorded using an Axon Digidata 1550B1 Low Noise system with HumSilencer (Molecular Devices). Command voltage waveforms were compensated for the liquid junction potential of −11 mV. Recordings were considered satisfactory and included in the final analysis only if, both before and after the recording, the patched cell had an access resistance (${R}_{\rm {a}}$) < 20 MΩ and a membrane resistance (${R}_{\rm {m}}$) $> 10\times {R}_{\rm{a}}$.

#### Whole-cell current clamp setup

The setup for the current clamp was identical to that of the voltage clamp (described above), with slight modifications. The resting membrane potential of HEK293-Kir2.1 cells was determined using a whole-cell current clamp mode in imaging solution number 1 (resting membrane potential: −83 mV ± 4 mV, s.d., *n* = 11) or imaging solution number 2 (resting membrane potential: −70.33 mV ± 0.44 mV, s.d., *n* = 4) and compensated by −11 mV to account for the liquid junction potential. HEK293-Kir2.1 cells were plated the day before patching.

#### Voltage clamp under 2P illumination

The same imaging hardware setup was used as described in the ‘2P GEVI screening system’ section. An oil-immersion 40× 1.3-NA objective (CFI Plan Fluor DIC H/N2, Nikon Instruments) was used to focus 940 nm of light set to ~61 mW onto the sample plane. Videos were taken with a resolution of 512 × 32 pixels and a frame rate of 440 Hz. Recordings where noise was indistinguishable from the signal due to the low brightness of the cell were removed from the final analysis. Electrophysiological recordings of action potential waveforms were done at room temperature (∼22 °C), and cells were held at –70 mV. We clamped HEK293 cells to a variety of typical action potential waveforms and monitored the resulting changes in GEVI fluorescence. We used an action potential waveform that was recorded from a representative hippocampal neuron and modified it to have an amplitude of 100 mV and an FWHM of 2 ms, to mimic the shape of layer 2/3 cortical neurons at room temperature^46^. To further improve the probability of detecting a response to the action potential waveform for sensors with slower on-kinetics, we also included action potentials widened to 4-ms FWHM. Because all sensors easily detected the 2-ms FWHM, we did not further characterize the 4-ms FWHM action potential waveforms. We performed experiments at room temperature because spikes are shorter at 37 °C (0.7–0.8 ms)^46–48^, and are thus suboptimally sampled with our acquisition rate (440 Hz under 2P microscopy). Because GEVIs’ response time constants decrease with temperature at about the same rate as the decrease in spike width, GEVI responses are often tested at room temperature in vitro^18,22,67–69^. Cells were stimulated with five action potential waveforms with a FWHM of 2 ms at 2 Hz, five action potential waveforms with a FWHM of 4 ms at 2 Hz and ten action potential waveforms with a FWHM of 2 ms at 100 Hz. In the same protocol, we also recorded GEVI response to a modified burst of action potentials recorded from adult mouse somatosensory cortex layer 5 pyramidal neurons, designed to mimic action potentials on top of subthreshold depolarizations. The spike burst exhibited a subthreshold depolarization of ~24 mV (from a baseline voltage of −70 mV) and action potentials with amplitudes of 60–90 mV (from a subthreshold voltage of −56 mV). The action potentials in the spike burst are 3–4 ms FWHM. The action potential waveform portion of the protocol was followed by a 2-s interval at −70 mV before the start of the voltage step protocol.

To characterize fluorescence changes at or near steady state, cells were submitted to 1-s voltage steps at −120 mV, −100 mV, −80 mV, −60 mV, −40 mV, −20 mV, 0 mV, 20 mV, 30 mV and 50 mV, followed by a 2-s interval at −70 mV and then a second set of voltage steps of −80 mV, −75 mV, −65 mV, −60 mV, −55 mV and −50 mV with a 1.5-s interval between each voltage step. The data from the voltage step protocol were collected at 22–23 °C or 32–33 °C. Fluorescence traces of the voltage steps were smoothed by a 47.6-ms moving average.

To quantify fluorescence response, the videos were analyzed using the same mathematical steps described in the ‘Analysis of high-throughput screening data’ section. We corrected the green (GEVI) fluorescence trace for photobleaching by dividing each trace by the best-fit three-term exponential or by using a sliding window of three frames. Fitting of the fluorescent trace for photobleaching correction was performed outside any voltage step. For each voltage step, the response is calculated as the mean over the 1‑s step. Because we averaged responses over an extended period, we did not subtract the baseline standard deviation, unlike in the section referenced above, where peak responses were measured. For each cell, the five 2-ms action potential waveforms were averaged before calculating the peak response amplitude and spike width (FWHM). For final visualization in Fig. 1I, the fluorescence response to a single-spike, 100-Hz spike train and spike-on-subthreshold waveform was upsampled to 1 kHz using linear interpolation.

#### Voltage clamp under 1P illumination

The same imaging hardware setup described in the ‘2P GEVI screening system’ section was used, with the following modifications. Cells were illuminated with 470/24-nm light (SpectraX, Lumencor) and conditioned using the 477–503-nm band of a multi-band dichroic mirror (89100bs, Chroma). The irradiance at the sample plane was ~4 mW/mm^2^. Green-emitted photons were reflected toward a multi-alkali PMT (PMM02, Thorlabs) installed on one of the side ports of the microscope using the 503–542-nm band of the multipass dichroic (89100bs, Chroma) and then filtered at 509–532 nm using a multipass filter (89101 m, Chroma). Kinetics were evaluated using three 1-s 100-mV depolarization pulses from −70 mV to 30 mV. To characterize kinetics at different voltage steps corresponding to hyperpolarization, subthreshold potentials and action potentials, we used three replicate 1-s steps for the following voltage steps: −70 mV to −100 mV, −70 mV to −45 mV and −45 mV to 30 mV. Between each pulse, cells were held at –70 mV for 1.4 s. Recordings were performed at 32–33 °C using a feedback-controlled inline heater system (inline heater SH-27B, controller TC-324C, cable with thermistor TA-29, Warner instruments) to maintain the temperature in the perfusion chamber. A diaphragm was used to reduce the diameter of the excitation spot so that during imaging, only one cell at the center of the FOV was illuminated. To maximize photon collection, an oil-immersion 40× 1.3-NA objective (CFI Plan Fluor DIC H/N2, Nikon Instruments) was used. A LabVIEW (NXG v.5.0, National Instruments) routine was used to control the PMT bias voltage and record the output voltage using the data acquisition and output boards. Data were collected at 80 kHz.

The output voltage from the PMT was analyzed by a custom routine written in MATLAB to obtain the fluorescence signal for each cell. The raw data were first downsampled to 20 kHz.

We corrected the trace for photobleaching by dividing each trace by the best-fit three-term exponential (when the cell was held at −70 mV). The traces from the three replicate voltage steps were averaged. The corrected averaged signal was cropped from 0.1 s before the estimated depolarization or the repolarization onset to 1 s after the estimated depolarization or repolarization onset, except for the −45-mV to −30-mV repolarization step, which was cropped 0.2 s after the estimated repolarization onset. The resulting kinetics traces were fitted with either a single-exponential model,

$$\begin{array}{l}{F}_{t}=\left\{\begin{array}{rr}k\times {e}^{(t-to)\lambda }+c, & t > {t}_{0}\\ k+c, & t\le {t}_{0}\end{array}\right.\end{array}$$

or a dual-exponential model,

$${F}_{t}=\left\{\begin{array}{rr}k\times {e}^{\left(t-to\right)\lambda }+{k}_{2}\times {e}^{(t-to){\lambda }_{2}}+c, & \,t > {t}_{0}\\ \,(k+{k}_{2})+c, & \,t\le {t}_{0}\end{array}\right.$$

where t is the independent variable, time, and F is the dependent variable, fluorescence response. The coefficient c describes the mean plateau fluorescence, k and ${k}_{2}$ describe the relative ratio of each exponential component, λ and ${\lambda }_{2}$ describe the negative inverse of the time constant(s), and ${t}_{0}$ is an offset indicating the exact event onset timing. In some instances, ${\lambda }_{2}$ describes a slow photobleaching component. For the −100-mV to −70-mV and the −70-mV to −100-mV steps, all three JEDI sensor kinetics were best fit by a dual-exponential function, where λ describes the off-kinetics and ${\lambda }_{2}$ describes a slow photobleaching component not reported in Table 1.

#### 2P excitation spectra

HEK293 Kir2.1 cells were cultured and transfected in 96-well plates with either JEDI3hyp, JEDI3sub, JEDI-2P or GFP as discussed in the “Cell culture and transfection in 96-well plates” section. Cells were washed and imaged in imaging solution number 1. Images were acquired using the same microscope as described in the ‘2P GEVI screening system’ section. Laser pulses were not pre-compensated for dispersion in the microscope optical path. Excitation wavelengths from 700 to 1080 nm were used in 10-nm increments and set to a power of 16–18 mW at the sample plane, as measured by a microscope slide power sensor (S170C Thorlabs). Each 512 × 512 FOV was imaged at each wavelength using two galvanometer optical scanners set to acquire 1.1 s images.

Images were background corrected by subtracting the mode intensity of the first frame’s pixel intensity histogram, excluding saturated pixels. A maximum intensity projection, excluding saturating pixels, across all images was used to determine values for foreground segmentation using a user-defined threshold to optimize cellular masking for all constructs. The mean intensity of the foreground pixels was calculated for each wavelength. Slight deviations in the power between wavelengths were corrected by assuming a quadratic dependence of fluorescence on illumination power at the sample plane.

#### 2P photostability characterization

HEK293 Kir2.1 cells were cultured and transfected in 96-well plates with JEDI3hyp, JEDI3sub, JEDI-2P or GFP as discussed in the ‘Cell culture and transfection in 96-well plates’ section. Cells were imaged using the same microscope and objective as listed in ‘2P GEVI screening system’. Cells were washed and imaged in imaging solution number 1. Photobleaching was conducted at 940 nm with power ranging from 29 mW to 101 mW at the sample plane. Each well had six separate FOVs of 512 × 32 pixels that were continuously imaged for 6.05 min at 440 Hz. The emission light from the cell was split using a 560-nm dichroic mirror (348958, Chroma), filtered by a 525/50-nm bandpass filter (353716, Chroma) and collected by a GaAsP PMT.

To quantify GEVI photostability under 2P, every 10th image from the video was loaded and analyzed. Foreground segmentation was done, followed by background correction using a user-defined threshold to optimize cellular masking for all constructs as described in the ‘Analysis of high-throughput screening data’ section. Saturating pixels were also removed from further analysis. The mean fluorescence from the foreground pixels was normalized to the fluorescence of the first frame of the video. The AUC of the normalized photobleaching traces, over the entire (6.05 min) time courses, was computed using the trapezoid rule (GraphPad Prism v.10.6.1 (892)). The AUC provides a robust, model-independent measure of photostability across illumination power levels. This approach avoids the complications associated with fitting photobleaching traces, which often require multiple exponential components whose number and relative weights can vary with illumination intensity, complicating statistical analysis. To facilitate comparison, AUC values were normalized to the maximal AUC possible for that recording (that is, AUC for a straight line of *y* = 1 for the same duration of the recording), such that each data point represents a GEVI’s integrated fluorescence intensity as a percentage of the total signal that would be produced by an ideal, non-bleaching (infinitely photostable) GEVI. AUCs were calculated from *n* = 3–4 independent transfections per power level. The Kruskal–Wallis test, followed by a Dunn’s multiple-comparisons test, was used to determine if the relationship between ‘integrated fluorescence intensity versus power’ differed between the JEDI3 sensors and JEDI-2P. The fitting of the 37-mW fluorescence traces was performed using a double exponential equation that modeled a two-phase decay.

#### GEVI brightness quantification

HEK293 Kir2.1 cells were cultured and transfected with JEDI3hyp, JEDI3sub, ASAP5 or JEDI-2P all with a C-terminal GSSGSSGSS-linked mBeRFP reference protein. Cells were transfected and grown in 96-well plates, and cells were imaged in imaging solution number 2 as described in ‘Cell culture and transfection in 96-well plates’. Brightness images were acquired at 940 nm with a power of 99 mW at the sample plane. Each well had four separate FOVs of 1,024 × 1,024 pixels that were captured using two galvanometer optical scanners at a rate of 2.4 s per frame. Target (green) and reference (red) emission light was collected using the same imaging system as described in ‘2P GEVI screening system’.

Quantification of GEVI brightness on a single-cell level was performed using in-house-developed Python software (3.12.10), which generates whole-cell masks using Cellpose3 for cell segmentation^70^. The background signal for both target and reference channels was estimated as the median fluorescence intensity in the inverse of the cell mask, excluding non-cell-containing regions. Cells were excluded from analysis if their mean fluorescence did not exceed twice the mean background signal or if more than 5% of pixels within the cell were saturated. To restrict measurements to the plasma membrane, a Laplacian filter was applied, followed by an inward-directed Gaussian blur, which corresponded to a width of 1 μm. Background subtraction was performed using user-defined thresholds to optimize cellular masking for all constructs. For each cell, the total fluorescence intensity was quantified in both the target and reference channels. Cells with outlier fluorescence intensities in either channel were removed from the final analysis using the ROUT test with a *Q* value of 1%. Brightness was calculated as the ratio of the target to the reference signal.

#### Confocal imaging of GEVI in dissociated neurons

Embryonic day 18 mouse hippocampal neurons were purchased from Transnetyx tissue by BrainBits (SKU SDEDHP, Transnetyx) and came in Hibernate EB complete Media (BrainBits HEB, 100 ml) as single-cell suspensions. Before seeding the hippocampal neurons, a 24-well glass-bottom plate (P24-1.5H-N, Cellvis) was coated with Neuron Coating Solution (027-05, Sigma-Aldrich) at 37 °C overnight and then washed three times with PBS. Neurons were seeded at 80,000–100,000 cells per ml in 500 µl glutamate-supplemented NbActiv1 medium (NB1 + GLU, Transnetyx) per well of a 24-well plate, following the manufacturer’s protocol. The day of plating was considered days in vitro (DIV) 0. Every 3–4 days, half of the medium was replaced with pre-equilibrated NbActiv4 medium (SKU NB4, Transnetyx). Transfection was conducted on DIV 12–14 using 1 µl lipofectamine 2000 and 800 ng total DNA, a mix of 150 ng pAAV-hSyn-JEDI3hyp or pAAV-hSyn-JEDI3sub and 650 ng pNCST bacterial expression vector (pNCST backbone is based on Addgene plasmid number 129508) as buffer/filler DNA.

Two days after transfection (DIV 14–16), the attached neurons were washed twice with imaging solution number 1 before adding 500 µl of imaging solution number 1 per well. Laser-scanning confocal images were obtained using a high-speed confocal microscope (LSM880 with Airyscan, Zeiss) controlled by the Zen software (v.2.3 SP1 FP3 black edition, Zeiss). The microscope was equipped with a 40× 1.1-NA water-immersion objective (LD C-Apochromat Korr M27, Zeiss), a 488-nm argon laser (LGK7812, Lasos) set to deliver 10 μW at the sample plane, and a per-pixel dwell time of 1.48–1.51 μs (JEDI3sub) or 1.51–3.36 μs (JEDI3hyp). Emission light was filtered using a multipass beamsplitter (MBS 488/561/633, Zeiss) and acquired with a 32-channel GaAsP detector (Airyscan, Zeiss) with a detector gain of 747 to 800, and a 1.08–1.92 Airy unit pinhole size. Images were acquired at a resolution of 0.04 µm per pixel and with an *x*–*y* dimension of 2,752 × 2,752 pixels or 4,972 × 4,972 pixels. *z*-stacks were stepped at 4–12 µm between images. To increase the SNR, four or eight scans were performed and averaged for each image. Airyscan processing was applied to the images using Zen software (v.2.3, Blue Edition, Zeiss) to increase the resolution. The overall images and dendrite zoom-ins are from the maximum projection of the *z*-stack, whereas the soma image is a single slice. The intensity of pixels varies between maximum projections and single slices; thus, we adjusted the look-up tables for optimal visualization of sensor expression only on the final slice or compiled maximum projection.

#### Cloning and packaging AAVs for in vitro experiments

All cloning of AAV transfer plasmids was performed in-house using In-Fusion cloning techniques discussed in the ‘Plasmid construction’ section. Using the pAAV-EF1a-DIO-JEDI-2P-Kv-WPRE plasmid (Addgene, 179459), we cloned JEDI3hyp and JEDI3sub in place of JEDI-2P and packaged the construct into an AAV serotype 1 (AAV1). Using the pAAV-hSyn-JEDI-2P-Kv-WPRE plasmid (Addgene, 179463), we cloned JEDI3sub in place of JEDI-2P and packaged the construct into an AAV9 serotype. The Kv tag promotes enrichment of the GEVI in the soma^18,33^. Using the pAAV-Ef1a-fDIO-GCaMP6s plasmid (Addgene, 105714), we replaced GCaMP6s with JEDI3hyp and packaged the construct into an AAV1 serotype. Unless otherwise stated, all packaging occurred at the BCM Neuroconnectivity Core.

### Optical recordings in the mouse cortex using ULoVE

All protocols adhered to the guidelines of the French National Ethics Committee for Sciences and Health, as outlined in the report on Ethical Principles for Animal Experimentation, in agreement with the European Community Directive 86/609/EEC, under agreement number 29791.

#### Animal handling, viral injections and surgeries

Twelve male wild-type (WT) C57BL/6J (Charles River Laboratories: https://www.criver.com/) adult mice (greater than postnatal day 40, body weight 20–24 g; *n* = 3 for JEDI-2P, *n* = 2 for JEDI3hyp, *n* = 4 for JEDI3sub and *n* = 3 for ASAP5)^11^ were housed in standard conditions (12-h light–dark cycles, light on at 7:00, with water and food ad libitum). As a preoperative analgesic, buprenorphine, 0.1 mg per kilogram of body weight, was used, and ketamine–xylazine was used as an anesthetic (Centravet). The AAV2/1-EF1a-DIO-JEDI-2P-Kv-WPRE virus was produced^71^ in-house at IBENS from Addgene clone number 179459 using a plasmid ratio (µg) of transgene:capsid:helper: 1:1.6:2. The AAV2/1-EF1a-DIO-JEDI-2P-Kv-WPRE, AAV2/1-EF1a-DIO-JEDI3hyp-Kv-WPRE, AAV2/1-EF1a-DIO-JEDI3sub-Kv-WPRE and AAV2/9-EF1a-DIO-ASAP5-Kv-WPRE^11^ viruses were injected at a titer of 3 × 10^12^ genome copies (GCs) per ml. All in vivo ASAP5-kv data are from Hao et al.^11^. To obtain a sparse density of GEVI-expressing neurons, AAV2/1 hSyn-Cre-WPRE-hGH (Addgene, AV-1-PV2676) was co-injected at a final titer of 2 × 10^9^ GCs per ml.

A GEVI and Cre AAV were combined in a saline solution containing 0.001% of pluronic acid (Thermo Fisher, 24040032), 300 nl of which was injected at a flow rate of 75 nl min^−1^ into the visual cortex (V1 coordinates from bregma: anteroposterior −3/−3.5 mm, mediolateral −2.5/−3 mm, and dorsoventral −0.3 mm from brain surface). A custom-designed aluminum headplate was fixed on the skull with layers of dental cement (Metabond). A 5-mm-diameter number 1 coverslip was placed on top of the visual cortex and secured with dental cement (Tetric Evoflow). Mice were allowed to recover for at least 15 days before recording sessions and were housed in groups of at least two mice per cage. Imaging was done between 15 and 77 days after surgery. Behavioral habituation was adopted, involving progressive handling by the experimenter with gradual increases in head-fixation duration^72^. Mice were handled before recording sessions to minimize restraint-associated stress, and experiments were conducted during the light cycle.

#### ULoVE voltage optical recording and experimental design

Recording sessions lasted 1–3 h and were conducted while mice behaved spontaneously on top of an unconstrained running wheel in the dark^72^. Recordings were performed using a custom-designed AOD-based random-access multiphoton system (Karthala System) based on a previously described design^22^. The excitation was provided by a femtosecond laser (InSight X3, Spectra Physics) mode-locked at 920 nm with a repetition rate of 80 MHz. A water-immersion objective (CFO Apo25XC W1300, 1.1 NA, 2-mm working distance, Nikon) was used for excitation and epifluorescence light collection. This experiment was done at 920 nm because the peak 2P excitation wavelength for the JEDI3 sensors had not yet been determined. The signal was passed through an IR blocking filter (TF1, Thorlabs), split into two channels using a 562-nm dichroic mirror (Semrock), and passed to two H12056P-40 PMTs (Hamamatsu) operating in photon-counting mode. The 510/84 filtered green channel was used for collecting JEDI signals. The laser power was set to deliver 15 mW using a post-objective and pre-sample measurement method, then adjusted for mono-exponential loss through tissue with a length constant of 170 µm. To locate neurons, before making ULoVE optical recordings, the AOD-based microscope was used in sequential point scanning mode to form an image. To produce the images in Fig. 2b and Extended Data Figs. 2a, 3a,e, 4a and 5a, a time series of 50 images (1 µs per pixel, 0.091 µm per pixel) was acquired and post hoc motion registered. Cell depth was measured from the brain surface.

The optimized ULoVE excitation pattern (Lombardini et al., in preparation) consists of a series of nine points vertically aligned (evenly spaced over a distance of 15 µm), multiplexed twice horizontally with a 2-µm spacing. The 18 points were scanned diagonally (8 µm horizontally and 3 µm vertically) during a 50-µs acquisition time, to homogeneously fill an extended excitation volume that continually encompasses the cell plasma membrane. This latest strategy refines the axial profile of excitation, thereby limiting signals outside the desired focal plane and reducing neuropil signal contamination, which improves SNR. Compared with the power used in the imaging mode, laser power was multiplied by 1.5 to account for the greater excitation volume. The applied power never exceeded 200 mW. Using two patterns per cell enabled a temporal resolution of 7,142.9 Hz. Recordings were stopped after 320 s (5.33 min). For recording, we selected neurons that were sufficiently bright to obtain a substantial SNR, yet did not display long-lasting depolarizing plateaus, which we believe indicate overexpression.

#### Signal analysis, spike extraction and waveform analysis

For each cell recording, two ULoVE volumes were simultaneously used as ‘regions/volumes’ of interest (Fig. 2b); thus, the traces represent the sum of photons collected from the two volumes. Photobleaching was assessed by bi-exponential fitting and corrected by division of the raw trace using the best fit bi-exponential function. This analysis enabled the full trace to be corrected for the fast-photobleaching component. To characterize the second phase (from 60 s to the end of the recording), which could be more a consequence of a diffusion process described by a power law, the traces were low-pass filtered at 1 Hz. Traces were downsampled 140 times and plotted on a log_10_–log_10_ scale, then fitted with a line, to determine the slope of the power law. The following steps were used to generate the %Δ*F*/*F*_0_ traces: the raw traces were normalized by the bi-exponential fit function (computed above), and the remaining low-frequency drift was removed using a zero-phase distortion filter (high pass, 0.5 Hz). The traces were then converted to %Δ*F*/*F*_0_, with the mean signal used as *F*_0_.

Spikes were detected using a custom-designed algorithm, which utilizes three metrics to sort aspects of spike shape and threshold, all with *z*-scores above 3. The first metric is to high-pass filter the trace (using a second-order Butterworth filter with a lower limit of 40 Hz). The second metric is a cumulative probability transformation of the signal using the standard erf function for a duration of 1.6 ms. The third metric calculates the cumulative product of the 2nd to the 5th scale of the coif1 discrete wavelet transform and applies a global realignment to retrieve energy in various frequency bands in one peak.

To quantify spike amplitudes (Fig. 2d), an average spike waveform was extracted from isolated spikes. Single spikes that were separated by more than 50 ms were aligned to the spike onset and then averaged to produce the average spike waveform (Fig. 2c and Extended Data Fig. 5c). To assess how the inclusion of burst events affects the average trace, spike-triggered averages computed from all detected spikes are also shown in Extended Data Fig. 5c. Spike amplitude was then calculated from the peak value of the average spike waveform obtained only from isolated spikes, measured from the onset point. FWHM corresponds to the extent in time at half-maximum amplitude, taken from the average spike waveform after 20-kHz linear interpolation.

To quantify subthreshold fluctuations, traces were filtered using a bidirectional Butterworth bandpass filter with a frequency range of 0.1 Hz to 50 Hz. We restricted our analysis to periods when the animal’s speed was below 1 cm s^−1^. The cell’s low state corresponds to the 1st percentile of that filtered trace. The cell’s high state corresponds to the median of the signals at the detected spike onsets. The subthreshold fluctuation is defined as the difference between the cell’s low state and its high state. The dynamic range is the sum of the spike amplitude and the subthreshold fluctuation.

#### Plasma-membrane localization analysis of JEDI sensors

Images were taken before ULoVE recordings, acquired using the same AOD-based microscope in imaging mode. A time series of 50 images (1 µs per pixel, 0.091 µm per pixel) was acquired, and post hoc motion registration was applied to produce the images in Fig. 2b and Extended Data Fig. 2a. To quantify membrane localization, individual cells were first identified using Cellpose3 to generate whole-cell masks^70^. A Laplacian filter was then applied to isolate pixels located at the edge of each cell, allowing for a Euclidean distance transformation to be applied to index each pixel’s minimum distance from the cell boundary. By combining this transformation with the normalized intensity of each cell, we were able to project intensity as a function of distance from the cell edge, providing a holistic view of signal distribution relative to the cell boundary.

To analyze this distribution, we fit a Gaussian function to the initial rising portion of the intensity trace, which corresponds to the transition from the extracellular to the intracellular space. Then, by taking the difference between the indefinite integral of our Gaussian fit and the raw trace, we can determine both the percentage of signal that can be attributed to plasma-membrane and non-plasma-membrane structures.

#### Evaluating the contribution of scanning and motion artifacts to fluorescence signals

##### Fast AOD-based videos and 3D stacks

Neurons expressing JEDI3sub-Kv (*n* = 5 from one C57BL/6 male mouse) were imaged using AOD-based fast linear 2P scanning in awake head-fixed animals (Karthala AODscope). A small square FOV was placed around the cell such that spacings between the FOV borders and the cell membrane were in the order of 3–4 µm, consistent with half the width of the ULoVE pattern (for example, Fig. 2 and Extended Data Fig. 3e). This configuration allowed sampling at a frame rate between 2,000 Hz and 2,666 Hz, and each cell was recorded for a duration of 2 min. We then used the average fluorescence vector (*F*), which was baseline (*F*_0_) subtracted (Δ*F* = *F* − *F*_0_) and normalized to obtain the unit-free metric Δ*F/F*_0_. *F*_0_ was calculated from *F* by computing the 90th percentile of the fluorescence distribution obtained over a 2-s window centered on the corresponding time bin. For each neuron, we also acquired a 3D stack spanning the entire cell body, with a step of 0.5 µm between each plane (Extended Data Fig. 3a).

##### Extracting *x*, *y*, *z* shifts and measuring 3D Euclidean norm

We quantified motion-induced shifts in each direction of the space using two-dimensional (2D) cross-correlation between our scanned images and a 2D or 3D template (Extended Data Fig. 6b,c). In short, the 2D cross-correlation computes a normalized 2D convolution between an image and a template. The resulting values then peak where the image and the template are most similar, allowing us to infer spatial shifts of a neuron at each point in time. For the *x-* and *y*-directions, we used the widely used and openly available NoRMCorre algorithm^73^ to quantify *x* and *y* shifts. For the *z*-direction, we applied the same reasoning but extended it by one dimension: we computed the maximum 2D cross-correlation between the image and each plane of the 3D template. We then determined the depth of the current imaging plane from the index at which the value of the 2D cross-correlation was maximal. Zero-centering of the *z*-shift vector was performed on the median depth, and each shift vector was converted to µm, given known step and pixel sizes. For each recording, at each time bin *t*, we also defined a triplet (*x*_*t*_, *y*_*t*_, *z*_*t*_) whose elements are the shift values in each direction of space. We then computed the distance from the origin of the (*x*_*t*_, *y*_*t*_, *z*_*t*_) points in the 3D space using the Euclidean norm: ||3D motion | |_2_(*t*) = √(*x*_*t*_^2 ^+ *y*_*t*_^2 ^+ *z*_*t*_^2^), defining the entries of a global shift vector. Pearson correlations were calculated between the Euclidean norm and GEVI signals (∆*F*/*F*_0_) for all timings where the displacement values were below 2.5 µm (due to the small image size).

##### Extracting spatiotemporal plasma-membrane information from ULoVE recordings

GEVI-expressing neurons shown in Fig. 2 were recorded using ULoVE illumination patterns that provide spatial and temporal information of the GEVI-targeted membrane (Fig. 2 and Extended Data Fig. 3e). One can, therefore, quantify drifting of the GEVI-labeled plasma membrane over time using cross-correlation between the membrane profiles of a reference time bin and recording time bins. We first processed data obtained from each ULoVE excitation volume by applying spatial (1.5 µm) and temporal (20 ms) smoothing. Then a normalized cross-correlation between individual time bins was calculated. This generated a template that is automatically defined as the most commonly found profile across a set of candidate templates. In the case of unimodal profiles such as the neuronal membrane, we found that weighting the cross-correlogram by the original neuron profile enabled us to obtain an accurate estimation of the drift from the maximum index of the weighted cross-correlogram (Extended Data Fig. 3e).

##### Inversion of fluorescence polarity in Supplementary Video 2

To match the inverted display of fluorescence traces (expressed as −%Δ*F*/*F*_0_), we processed the temporal stack displayed on the left panel of Supplementary Video 1 as follows. Let **S** be a 3D stack of images obtained using high-speed 2P scanning and let **M** be the matrix of average pixel values across time (note that **M** is the size of individual images). We subtracted each image of the stack from this temporal average matrix (**ΔS** = **M** − **S**) to flip the fluorescence modulations of each pixel around their average value, before adding back the temporal average to get an inverted stack **S**_**inv**_ having the same average values as the original stack, but inverted dynamics (**S**_**inv** _= **ΔS** + **M**).

### JEDI3sub tracking of multi-cell subthreshold optical tuning with FACED microscopy

All animal experiments were conducted according to the National Institutes of Health (NIH) guidelines for animal research. Procedures and protocols on mice were approved by the Institutional Animal Care and Use Committee at the University of California, Berkeley.

#### Animal handling, craniotomy and virus injection

The mice were housed individually or in pairs in an animal facility on the University of California, Berkeley campus, with a 12-h light–dark cycle, ambient temperatures between 20 °C and 26 °C, and humidity levels between 40% and 60%. Virus injection and cranial window implantation^35^ was done on two male mice (94 days old). Mice were anesthetized with isoflurane (1–2% by volume in oxygen) and administered the analgesic buprenorphine (subcutaneously, 0.3 mg per kilogram of body weight). Animals were head-fixed in a stereotaxic apparatus (Model 1900, David Kopf Instruments). A 3.5-mm-diameter craniotomy was made over the left visual cortex (−3.5 mm posterior and 2.5 mm lateral from bregma) with dura intact. WT mice (C57BL/6J, Jackson Laboratories) were used, and virus injection was performed to express pAAV-hSyn-JEDI3sub-Kv-WPRE (1.17 × 10^12^ GCs per ml, 10× dilution), using a glass pipette with a 15–20-μm opening and a 45° bevel. The pipette was backfilled with mineral oil, and a fitted plunger controlled by a hydraulic manipulator (Narishige, MO10) was inserted into the pipette for loading. Virus was injected slowly at 6–12 sites (50 nl at each site) at a depth of 250 μm within the left visual cortex. A glass window made of a single coverslip (Fisher Scientific number 1.5) was embedded in the craniotomy and sealed in place using dental acrylic. Then, a titanium head-post was affixed to the skull using cyanoacrylate glue and dental acrylic. In vivo imaging was conducted after a 3-week recovery period. All imaging experiments were conducted on head-fixed, awake mice.

#### Voltage imaging with FACED microscopy

We imaged subthreshold voltage activity from a group of L2/3 neurons using a custom fluorescence microscope^30,31^. Details of light path and data acquisition code were previously described^30,31^. Output from a 1,035-nm fiber laser (Monaco 1035-40-40, 1-MHz repetition rate; Coherent) was focused by a cylindrical lens and then sent into a pair of mirrors with a small tilt angle. Based on FACED^74^, the laser pulse was split into multiple pulses that were spatially separated and temporarily delayed by the mirror pair, forming a one-dimensional array of excitation foci at the objective focal plane with a line-scanning rate of 1 MHz. A galvanometer scanned excitation foci in directions perpendicular to the line scans to form 2D images, and an additional galvanometer tiled the 2D images to cover a larger FOV. With a 25× 1.05-NA objective (XLPLN25XWMP2; Olympus), we imaged L2/3 neurons in the mouse visual cortex at depths ranging from 110 µm to 160 µm over a 320 × 400 µm or 160 × 400 µm FOV at a frame rate of 400 Hz or 769 Hz, respectively, at a post-objective excitation power of 156 mW.

#### Visual stimulation

A custom script written using the Psychophysics Toolbox^75^ in MATLAB (MathWorks, R2018b) was used to display drifting grating visual stimulation on an LCD monitor to the right eye of the mouse. Each visual stimulation trial consisted of grating drifts in eight directions, from 0° to 315° in 45° intervals, with a 0.5-s blank period preceding each 0.5-s drifting grating. For each FOV, 24–26 trials were acquired.

#### Image analysis

Images were analyzed using custom programs in MATLAB (MathWorks, R2024a). Time-lapse images were motion registered using an iterative cross-correlation method^35^, with the time-averaged image from the first trial serving as the registration target for the remaining trials. Cell masks, or regions of interest (ROIs) were first selected using the automatic cell segmentation pipeline Cellpose (2.0)^76^ and were then manually curated to add new ROIs and remove incorrectly identified regions. The average signal per pixel within each ROI was extracted as the raw neuronal trace (*F*_raw_). As photobleaching was minimal across the experiment (~8%; Extended Data Fig. 6a), fluorescence traces were not corrected for photobleaching.

Because the Kv tag reduces but does not eliminate JEDI3sub expression in the neuropil, we performed neuropil subtraction^77,78^. Following a common convention for neuropil subtraction in calcium imaging^79^, the fluorescence trace for each ROI was calculated as *F*_ROI_ = *F*_raw_ − 0.7 × *F*_neuropil_, where *F*_neuropil_ represents the average fluorescence of pixels within a neuropil mask surrounding each ROI. To obtain the neuropil mask, a ring was grown around the ROI, and growth was stopped when the mask size reached 300 pixels. Pixels from other ROIs and pixels with extremely high intensity values (top 4% of the whole FOV, corresponding to extracellular fluorescent puncta) were removed before generating neuropil masks. On average, the neuropil mask size was 1.4 times the size of the ROI. ROIs with *F*_neuropil_ > 1.1 × *F*_raw_ were excluded before further analysis. No other exclusion criteria were used for cell selection.

To calculate Δ*F/F*, the baseline signal (*F*) was obtained by averaging the signal from 0.3 s to 0.5 s during the blank period preceding each grating stimulus, and Δ*F* was defined as *F*_raw_ − *F*, the difference between the raw trace *F*_raw_ and the baseline *F*. To calculate the subthreshold activity, the raw −Δ*F/F* trace was low-pass filtered with a 3rd-order, 12-Hz Butterworth filter. The mean −Δ*F/F* value during the 0.5-s stimulation period and the 0.3-s blank period following the stimulation period was defined as the subthreshold response R to the stimulation. The representative FOV (Fig. 3b) is a motion-registered image across frames and trials, with line illumination correction (accounting for FACED non-uniform illumination in the horizontal direction), for display purposes only.

To determine whether a cell had visually evoked subthreshold activity, a one-tailed *t*-test was performed for each drifting direction to compare whether the subthreshold response to the stimulation R (as defined above) was significantly larger than the average −Δ*F/F* during the final 0.2 s of the preceding blank period. If the *P* value was less than 0.0063 (0.05/8, correction for multiple comparisons), then it was considered a visually evoked cell. A visually evoked cell was considered to have orientation selectivity if the *P* value from a one-way ANOVA test, which assessed how subthreshold responses vary with the direction of drifting grating, was less than 0.05.

To quantify the orientation tuning properties of visually evoked cells, their subthreshold response R to each stimulation orientation and stimulation orientation angle (θ) were fitted using a bimodal Gaussian function^80^ as shown in equation (1):

1 $$\begin{array}{l}R(\theta )={C}_{\mathrm{offset}}+{C}_{\mathrm{pref}}\exp \left(-\frac{{\mathrm{ang}(\theta\,-\,{\theta }_{\mathrm{pref}})}^{2}}{2{\sigma }^{2}}\right)\\ \,\,\,\,\,\,\,\,\,\,\,\,+\,{C}_{\mathrm{oppo}}\exp \left(-\frac{{\mathrm{ang}(\theta\,-\,{\theta }_{\mathrm{pref}}\,+\,180)}^{2}}{2{\sigma }^{2}}\right).\end{array}$$

Here ${\theta }_{\mathrm{pref}}$ is the preferred orientation and ${C}_{\mathrm{pref}}$ and ${C}_{\mathrm{oppo}}$ are the response coefficients at ${\theta }_{\mathrm{pref}}$ and ${\theta }_{\mathrm{pref}}-180$, respectively. The ${\rm{ang}}(\theta )$ wraps θ to the interval of 0° to 180°. The σ is the standard deviation of the Gaussian function. The global orientation-selectivity index of the subthreshold response is defined according to equation (2)^35^:

2 $$\frac{|{\sum }_{k}R({\theta }_{k}){\rm{e}}^{{\rm{i}}2{\theta }_{k}}|}{{\sum }_{k}R({\theta }_{k})},$$

where k is the orientation index.

Correlations between cell pairs were analyzed by calculating the Pearson’s correlation coefficient of the responses between each cell pair^81^.

### JEDI3hyp tracks SPW-Rs in PV^+^ hippocampal interneurons

All procedures were conducted in accordance with the NIH guidelines and with the approval of the Institutional Animal Care and Use Committee of Baylor College of Medicine.

#### Animal handling, viral injections and surgeries

Four adult female mice (8 weeks or older at the start of the experiment) were used. Mice were housed in groups of 2–5 animals on a standard diet and maintained under a 12-h light/12-h dark cycle. PV-Cre mice were obtained from JAX (017320) and were bred as homozygous pairs.

Mice were anesthetized with isoflurane and treated with analgesics (bupivacaine and buprenorphine). The skull was exposed, and a hole was drilled above the right dorsal hippocampus. Mice were injected in the dorsal CA1 (2.3 mm posterior, 1.5 mm lateral, 1.45–1.35 mm ventral to bregma) using a 1-μl Neuros (Hamilton) syringe, with AAV2/1-EF1a-DIO-JEDI3hyp-Kv-WPRE (400 nl each, full titer at 4 × 10^12^ GCs per ml). Injection was initiated ventrally, and the virus was delivered in pulses of 50–100 nl while gradually raising the syringe within the dorsoventral range, with 1-min intervals between pulses. JEDI3hyp virus was generated at the CNP Viral Vector Core at the CERVO Research Center (RRID: SCR_016477).

Surgeries to implant chronic imaging windows and headbars were performed after 5 or more days after viral injections. Mice were surgically implanted with an imaging window made of a round glass coverslip (diameter of 3 mm, Warner), attached to a stainless-steel cylinder (diameter of 3.18 mm, McMaster-Carr) with Narland optical adhesive. Mice were anesthetized with isoflurane and treated with analgesics (bupivacaine and buprenorphine), the skull was exposed, and a 3.0-mm-diameter circular craniotomy centered on the injection site was marked with a robot stereotax (Neurostar) and finished with a hand-held drill (Osada). The dura and cortical layers were removed by vacuum suction while flushing the brain with ice-cold cortex buffer^82^ (125 mM NaCl, 5 mM KCl, 10 mM glucose, 10 mM HEPES, 2 mM CaCl_2_ and 2 mM MgSO_4_ in distilled H_2_O with a pH of 7.4 and filter-sterilized) to minimize bleeding. The cannula was inserted and secured with tissue adhesive (Vetbond). The cannula and the exposed skull were covered with self-etching UV-curable adhesive (Optibond), and a stainless-steel headbar was attached with dental cement (Teets). After postoperative recovery, mice were returned to their home cages in the vivarium.

After recovery from surgery and confirming clean optical access to the hippocampus (10–14 days after surgery), mice were implanted with chronic hippocampal electrodes. A craniotomy was made, and a twisted tungsten wire pair made with 0.5-mm tip separation (0.002 inch, A-M systems) was lowered in the dorsal CA1 mirroring the virus injection site. The probe was stabilized using UV-curable glue, and the connector pins (A-M systems) were attached to the headbar implant with dental cement. Mice were allowed to recover for at least a day before imaging.

#### In vivo 2P voltage imaging and experimental design

In vivo imaging at 940 nm was performed using a setup consisting of the following: a 2P 8-kHz resonant scanner microscope (Bruker Investigator), a pulsed infrared laser (Spectra Physics MaiTai eHP-DS) and a 16× objective (0.8 NA WI, Nikon). Laser power was controlled by a Pockels cell and was set to 100–200 mW (measured at the objective) depending on sample brightness. The light path comprised a 700-nm beam splitter, a 565-nm beam splitter (for the dual-channel detector) and a 525/70-nm emission filter for JEDI3hyp imaging. Image acquisition was controlled by Prairie View 5.8 (Bruker). The image size was configured to 48 lines per frame, with 256 pixels per line, resulting in 6 laser pulses per pixel and a frame rate of 288.8 Hz. Pixel size was configured anisotropically (0.94 µm × 1.87 µm in the *x* and *y* dimensions, respectively) to increase the FOV size relative to the frame rate. Recording sessions from the CA1 oriens or pyramidal layers lasted 100,000 frames (5.8 min), during which the mice were freely running or resting on a 2-m-long, 2-inch-wide cloth belt supported on bearing rollers on a custom treadmill. The treadmill position was recorded by a rotary encoder connected to a PyControl system, which was synchronized with the image acquisition system^83^. LFPs were recorded as a differential between both wire tips using a differential amplifier (Warner DP-304, 0.1 Hz to 1 kHz, 1,000× gain) connected to the digitizer of the microscope system, and digitized at 2 kHz.

#### Image and LFP signal processing

Videos were preprocessed, cells were segmented, and Δ*F*/*F* traces were computed in Python 3.6 and 3.9. After inspection of the traces, dim cells were excluded using an empirical, quantitative threshold. This threshold was set to 0.25 average peak photon flux per frame per pixel in the ROI. Photon fluxes were calculated using the procedure described in https://github.com/multiphoton-tools/compress-multiphoton/blob/main/notebooks/EvaluatePhotonSensitivity.ipynb (ref. ^84^). Rigid motion correction was performed using suite2p^85^. Then, the following steps were performed using a custom Python pipeline: (1) polygon ROIs were drawn manually on the average images; (2) mean pixel intensity was computed in each frame in each ROI; (3) the mean pixel intensity trace from a manually drawn, neuron soma-sized background region (without noticeable labeled profiles) was subtracted from each cell’s pixel intensity trace, resulting in the background-subtracted time series (*F*); (4) a 3rd-degree polynomial was fit on *F*, excluding frames when the mouse was running, and any fluorescence peaks over two standard deviations, to obtain the time-dependent baseline (*F*_0_)^44^; (5) Δ*F*/*F* was computed as (*F* − *F*_0_)/*F*_0_, then inverted (max(Δ*F*/*F*) − Δ*F*/*F*). Smoothed traces (used only for visualization, not for quantification) were computed using an exponentially weighted moving average (the ewm method of pandas). ROI pixels with an SNR (mean over standard deviation) greater than 1 were considered JEDI3hyp-positive pixels and included in further analyses.

The representative image in Fig. 4b is an average of registered images and was computed by Suite2p (‘meanImg’ attribute). The image was converted to RGB format using ImageJ (NIH) and resized (200% height) to account for anisotropic pixel size. Image contrast was enhanced using the Curves function of Photoshop (Adobe), applied uniformly to the entire image.

SPW-Rs were automatically detected from bandpass-filtered LFP traces (130–200 Hz). The algorithm previously published by Varga et al.^39^ was implemented in Python using the bessel, filtfilt and hilbert functions of scipy.signals. Events were curated manually to exclude false positives by an expert investigator who was blinded to the imaging data. Event onsets were defined as the time of the Hilbert envelope crossing a threshold set at three standard deviations above the mean. Ripple-band power in each imaging frame was calculated as the mean of the envelope during each sample measured during the frame, and the resulting time series was divided by the standard deviation.

SPW-R onset times were clustered using DBSCAN (eps = 1 s), and single events and the first events of clusters were included to calculate peri-event averages. Masks around the event onsets were determined such that each imaging frame was included in the average only once, even if consecutive events occurred closer than the investigated time window. Frames were only included when the mouse stayed immobile. Generation of average traces (fluorescence or ripple-band power) was calculated first from the cell-wise means. Then, the mean ± standard error (s.e.m.) of the cell-wise means was computed using the number of animals as the sample size for calculating the s.e.m. Response magnitudes were quantified by using the locations of the maximum and minimum of the post-ripple grand average (21 ms and 114 ms, respectively). Then, the intensity of each cell was averaged during 50-ms time windows centered on these locations. Finally, the baseline of the cell (a −200-ms to −100-ms window before ripple onset) was subtracted from the responses. To determine whether the responses differed from the baseline, statistical tests were performed on the animal-wise averages using scipy (one-sample, one-sided *t*-test, with an alternative hypothesis ‘greater’ for peak and ‘less’ for trough).

#### JEDI3hyp 2P *z*-stacks

In vivo 2P *z*-stacks (Extended Data Fig. 7a,b) were acquired from awake, head-fixed mice before any functional imaging, using the galvo-galvo modality of the 2P microscope, with 1,024× 1,024-pixel resolution, 1.36× 1.36-µm lateral pixel size and 5-µm axial step size. Images were converted to RGB format using ImageJ, and identically sized regions were cropped with Adobe Photoshop for each panel. Image contrast was enhanced using the Curves function of Photoshop 2024, applied uniformly to all images.

#### JEDI3hyp confocal *z*-stack

The confocal image in Extended Data Fig. 7c was captured after in vivo recordings had concluded. Mice were anesthetized by isoflurane, then injected intraperitoneally with ketamine (300 mg per kilogram of body weight) and xylazine (30 mg per kilogram of body weight) in saline. The animals were transcardially perfused with saline (9 ml 0.9% NaCl for 1 min) and then with fixative solution (4% paraformaldehyde and 0.9% NaCl in 0.1 M phosphate buffer). The fixative volume was 40–100 ml for each mouse. Perfused brains were then post-fixed in the same fixative for 24 h at 4 °C. Brains were sliced on a vibratome (VT 1000 S, Leica Biosystems). Coronal sections (50-µm thick) were washed in 0.1 M phosphate buffer and mounted on glass slides in Vectashield (Vector Laboratories). Confocal images were acquired on a Nikon C2 confocal microscope using a 63× 1.4-NA objective (80 × 80 × 100-nm resolution; *x*, *y*, *z*). Images were deconvolved using Huygens software (SVI), with the classic maximum likelihood estimation algorithm. The stacks were then cropped, downsampled to 500 nm *z*-steps, assembled into a montage and converted to RGB using ImageJ.

### JEDI3hyp optical tracking of brain state in the mouse cortex

All procedures were carried out in accordance with the ethical guidelines of the NIH and were approved by the Institutional Animal Care and Use Committee of Baylor College of Medicine. The following experiments were done at 920 nm because the peak 2P excitation wavelength for the JEDI3 sensors had not yet been determined. All mouse strains were sourced from our mouse breeding colony.

#### Animal handling, viral injections and surgeries for in vivo 2P imaging in mice

We use data from a total of 13 mice aged 6 weeks to 6 months. Anesthesia induction was performed with 3% isoflurane and maintained with 1.5% isoflurane throughout the surgical procedure. Mice were injected with 5 mg per kilogram of body weight meloxicam or ketoprofen subcutaneously before the start of surgery. Anesthetized mice were placed in a stereotaxic head holder (Kopf Instruments) with their body temperature maintained at 37 °C using a homeothermic blanket system (Somnosuite with RightTemp Module, Kent Scientific). The scalp is shaved and cleaned with alcohol pads and betadine, after which 0.5 ml of 0.5% bupivacaine is injected subcutaneously under the scalp. After waiting 10 min, approximately 1 cm^2^ of skin is removed above the skull along with the underlying fascia. The edges of the incision are sealed with a thin layer of surgical glue (VetBond, 3 M), followed shortly by the implantation of a 4-mm washer (Seastrom) to head fix the mouse using a custom, removable headbar. The washer was implanted using dental cement (C&B Metabond, Parkell) over the visual cortex (2.5–3 mm lateral and 1 mm anterior of the intersection of the midline and lambda suture). The mouse was subsequently transferred onto a small platform and held stationary via the headbar. Using a surgical drill and a 0.6-mm carbide burr, a ~4-mm craniotomy was made in the center of the washer. The cortical surface was washed and kept moist with ACSF (125 mM NaCl, 5 mM KCl, 10 mM glucose, 10 mM HEPES, 2 mM CaCl_2_, 2 mM MgSO_4_). The viruses were injected using a nano-injection pump (WPI). Following injection of viral construct(s), the dura was removed, avoiding damage to the cortex, and the craniotomy was secured and sealed with a 4-mm coverslip (Warner Instruments) and surgical glue (VetBond). Following the surgery, mice were housed singly in the mouse colony under standard conditions (12-h light–dark cycles) with water and food available ad libitum.

#### 2P voltage imaging of layer 1, 2/3 and 5 pyramidal neuron somata via resonant scanning

For layer 1 imaging experiments, JEDI3hyp expression was transduced by co-injecting diluted 1:100 AAV1-hSyn-Cre (Addgene viral prep number 105553, stock concentration: 2.3 × 10^13^ viral genomes per ml) with undiluted AAV2/1-EF1a-DIO-JEDI3hyp-Kv-WPRE (stock concentration: 1:43 × 10^12^ viral genomes per ml) into non-Cre expressing mice (WT, C57BL/6). WT mice were injected with total volumes ranging from 50 nl to 250 nl of the mixed viral constructs, 25–150 µm in depth. For L2/3 imaging experiments, JEDI3hyp expression was expressed perisomatically by co-injecting undiluted AAV2/1-EF1a-DIO-JEDI3hyp-Kv-WPRE (stock concentration: 1.43 × 10^12^ viral genomes per ml) with diluted 1:100 AAV1-CaMKII-0.4-Cre-SV40 (Addgene viral prep number 105558-AAV1, stock concentration: 1.4 × 10^13^ viral genomes per ml) into non-Cre-expressing mice (WT, C57BL/6). WT mice were injected with total injection volumes ranging from 500 nl to 1,000 nl of the mixed viral constructs, 200–350 µm in depth. For layer 5 IT-PN imaging experiments, JEDI3hyp expression was transduced in layer 5 IT-PN by co-injecting undiluted AAV2/1-EF1a-fDIO-JEDI3hyp-WPRE (stock concentration: 6.32 × 10^12^ viral genomes per ml) with diluted 1:50 to 1:500 AAV1-EF1a-DIO-FLPo-WPRE-hGHpA (Addgene viral prep number 87306-AAV1, stock concentration: 1.3 × 10^13^ viral genomes per ml) or undiluted AAV2/1-EF1a-DIO-JEDI3hyp-Kv-WPRE (stock concentration: 4 × 10^10^ viral genomes per ml) in Tlx-Cre mice (RRID: MMRRC_041158-UCD). Tlx-Cre mice were injected with total volumes ranging from 50 nl to 500 nl of the mixed viral constructs, 350 µm to 550 µm in depth.

In vivo neuronal recordings of five layer 1 neurons were obtained across two male WT mice. The imaging power of layer 1 somata was maintained at 15–18 mW for focal depths ranging from 52 µm to 99 µm. In vivo neuronal recordings of six layer 2/3 pyramidal neurons were obtained from two female WT mice. The imaging power of layer 2/3 pyramidal neurons was maintained at 15–25 mW for focal depths ranging from 100 µm to 350 µm in depth. In vivo neuronal recordings of 32 IT-PNs were obtained using 2P resonant imaging across four layer 5 intratelencephalic mice (two males/females). The imaging power of layer 5 somata was maintained between 77 mW and 115 mW at the objective for focal depths ranging from 443 µm to 567 µm.

2P resonant imaging was performed on a Thorlabs Bergamo resonant-scanning microscope, with excitation provided by a titanium:sapphire femtosecond laser (Chameleon Vision II, Coherent) tuned to 920 nm. Cells were imaged at a frame rate of 20–462 Hz. A 25× 1.1-NA objective lens was used for imaging (CFI75 Apochromat 25XC W, Nikon Instruments). A dichroic mirror split emission light into two channels: the green channel used a 525/50-nm filter, and the red channel used a 625/90-nm filter, before being collected by two PMTs. ScanImage (v.2017, Vidrio) was used to control the microscope and acquire imaging data. Pupil and running activity were obtained concurrently with fluorescence recordings.

JEDI3hyp photobleaching traces were acquired using the same resonant-scanning hardware described above. JEDI3hyp traces were at least 15 min long and were calculated from manually segmented masks of the soma across layers 1, 2/3 and 5. Power was measured out of the objective using a Thorlabs digital power meter (PM100D) with a thermal power sensor (S175C), using the same imaging parameters as those used for data collection. Fluorescence traces were trimmed to 15 min, interpolated to have the same number of samples, and filtered using a low-pass Butterworth filter with a cutoff frequency of 0.1 Hz. Each trace was normalized by dividing the mean fluorescence within the first 5 s of imaging. Fluorescence traces were then grouped by depth, and the average and standard error of the mean were computed for each group.

#### Simultaneous 2P voltage imaging of the layer 5 soma and layer 1 dendrites of pyramidal neurons via resonant scanning on a 2P-RAM mesoscope

For layer 5 somatodendritic imaging, a dual-recombinase approach was used in Tlx-Cre (RRID: MMRRC_041158-UCD) mice to ultra-sparsely label single layer 5 intratelencephalic somata and their apical dendritic arbors. Diluted (1:20,000 to 1:100,000) AAV1-EF1a-DIO-FLPo-WPRE-hGHpA (Addgene viral prep number 87306-AAV1, stock concentration: 1.3 × 10^13^ viral genomes per ml) was co-injected with undiluted AAV1-EF1a-fDIO-JEDI3hyp-WPRE (stock concentration: 6.32 × 10^12^ viral genomes per ml) approximately 500–650 µm in layer 5 intratelencephalic of Tlx-Cre mice.

We collected 16 recordings of layer 5 IT-PNs and their matching dendrites (44 dendritic masks in total across all recordings). Two male and two female Tlx-Cre mice were used for these experiments. Imaging was performed using two-layer 2P resonant scanning with a Thorlabs 2P-RAM mesoscope. Excitation light was delivered via a titanium:sapphire laser (Tiberius, Thorlabs) tuned to 920 nm and a custom 10× 0.6-NA objective. The mesoscope was controlled using ScanImage (Vidrio) software. Imaging ROIs (20–40 µm^2^) were placed over the soma and one or more matching apical dendrites. Imaging of multiple planes was performed at various volume rates, ranging from 20 Hz to 70 Hz. Layer 5 somas were imaged at depths of 443–567 µm (77–115 mW), and dendrites were near-simultaneously imaged at 10–150 µm in depth (20–50 mW), taking advantage of the remote focus on the 2P-RAM microscope. Following functional imaging of individual somas and dendrites, high-resolution (1 pixel per µm) *z*-stacks with 80–120 steps were obtained, with a 5-µm spacing across the entire somatodendritic axis, spanning 400–600 µm in length. The structural *z*-stacks were then imported into Neuromantic (v.1.6.3)^86^ for the digital reconstruction of individual neurons and their apical dendrites. Representative images had brightness and intensity adjustments applied evenly across the image, and then a Gaussian smoothing filter was applied. Pupil and running activity were obtained concurrently with fluorescence recordings.

#### 2P voltage recordings of SOM^+^ and VIP^+^ interneurons using random-access microscopy

For experiments recording from VIP^+^ and SOM^+^ cells, JEDI3hyp expression was restricted perisomatically using diluted 1:10 AAV2/1-EF1a-DIO-JEDI3hyp-Kv-WPRE (stock concentration: 1.43 × 10^12^ viral genomes per ml). Around 500–1,000 nl was injected approximately 200–400 µm deep in VIP-Cre (RRID: IMSR_JAX:010908) or SOM-Cre (RRID: IMSR_JAX:013044) mice.

We acquired 111 in vivo recordings of VIP^+^ interneurons from five VIP-Cre mice (three male and two female) and 83 recordings from two male SOM-Cre mice. Optical recordings were conducted at an acquisition range between 396 Hz and 1,040 Hz using an AOD-based system (Femto3D Atlas Plug and Play AOD 2P microscope, Femtonics) with excitation at 920 nm (Alcor 920-2, Spark lasers). We used 0.8-NA 16× (Nikon; N16XLWD-PF) and 1.1-NA 25× (CFI75 Apochromat 25XC W, Nikon Instruments) objective lenses. Single and multiple VIP^+^ and SOM^+^ somas were simultaneously imaged using chessboard and ribbon 3D scan modes (FEMTO3D Atlas Software, Femtonics). Power was adjusted based on the depth of the soma and varied between 20 mW and 70 mW out of the objective. Pupil and running activity were obtained concurrently with fluorescence recordings.

#### Behavioral data acquisition and preprocessing

Following a 2-week recovery period after surgery, mice were placed on a freely moving treadmill and head-fixed using a custom headbar for 2P imaging. An optical encoder (McMaster-Carr) was used to track the rotation of the treadmill. Running periods were determined with a velocity threshold of 1 cm s^−1^ for at least 1 s. Running periods less than 3 s were consolidated into a single running period.

A camera (Dalsa) with a 1-inch hot mirror (Thorlabs) was used to record pupil activity at 20 fps. Since the experiments were performed in the dark without visual stimulation, the mouse was illuminated with a monitor backlight or UV light throughout in vivo 2P imaging experiments. Subsequently, a DeepLabCut (v.2.0.5) model identified and tracked pupil edges from which the pupil diameter was computed. We transformed these measurements into physical units using a pixel-to-mm conversion based on the typical size of the mouse eye, as previously detailed in ref. ^54^. Pupil periods were defined as pupil periods greater than one second with a mean dilation or constriction speed greater than 0.02 mm s^−1^. Eyelid blinks or frames with poor contrast where the pupil is not tracked by DeepLabCut were considered missing data (not a number, NaNs). Pupil periods composed of more than 25% NaNs were not analyzed. Given our interest in subtle brain-state changes occurring during quiet wakefulness, we excluded pupil dilation and constriction periods that occurred 3 s before, after and during running periods. Custom MATLAB (v.2023b) and LabVIEW (v.2016, National Instruments) code was used to control the acquisition and synchronization of imaging and behavioral data. The representative pupil image had brightness and intensity adjustments applied evenly across the image.

#### Preprocessing of voltage imaging data

Functional voltage imaging data were processed by using a processing pipeline implemented in Python (https://github.com/reimerlab/jedi3-paper/). Imaging data were motion-corrected in the horizontal plane for *x* and *y* shifts using a two-step algorithm. Large-scale motion was first corrected by taking the average template of the entire scan and computing the phase correlation between the average template and individual frames. Following motion correction of large horizontal shifts, smaller-scale motion was corrected by averaging frames of various window sizes (ranging from 3 s to 30 s, depending on which window size performed best) and computing the phase correlation between this local template and individual frames. Single-cell somatic imaging fields with motion correction greater than 3 μm in the *x*- and *y*-directions were excluded.

Somatodendritic imaging scans with greater than 3 µm of shifts in the somatic imaging plane or greater than 1 µm of shifts in the dendritic imaging plane were excluded for subsequent analysis. Scans were excluded from analysis if the imaged dendrites did not correspond to the imaged layer 5 soma. Scans with photodamage or imaging artifacts, such as a sudden increase or decrease in average field fluorescence due to water loss or external light contamination, that occurred in 10% or more of the total scan duration, were excluded. Following motion correction, VIP^+^ neurons, SOM^+^ neurons, layer 2/3 pyramidal neurons and layer 5 IT soma and dendrites were manually segmented from the average summary image. Raw fluorescence traces were then extracted from manually segmented masks before analysis.

#### Pupil phase binning: fluorescence response amplitude

We then examined the relationship between JEDI3hyp fluorescence and pupil phase for pyramidal cells and interneurons. To compute cell-wise fluorescence traces, pixels in manually segmented masks were averaged, the resulting signal was inverted so the fluorescence signal increases with depolarization, and a zero-phase 5-Hz Hamming low-pass filter was applied to denoise the trace. Because a standardized pre-event baseline interval would be confounded by the strong autocorrelation of the pupil trace, filtered fluorescence traces (F) were converted to response amplitude ($R=\Delta F/{F}_{0}=\frac{F-{F}_{0}}{{F}_{0}}$) using a rolling baseline. For layer 5 pyramidal neurons, ${F}_{0}$ was defined as the rolling bottom-5th percentile of a 20-min window. For VIP^+^ and SOM^+^ scans using the AOD-based system (FemtoAtlas 3D), we defined ${F}_{0}$ as the rolling median with a window of 20 s (twice as long as the duration of the longest pupil events), as this produced reduced sensitivity to motion artifacts. However, the finding that most VIP^+^ cells depolarize during pupil constriction was robust to multiple choices of baseline window size.

To assess overall dilation or constriction preference of cells, the mean −Δ*F*/*F*_0_ (denoted $- {\overline {\Delta F/{F}_{0}}}$) was first taken over each dilation and each constriction period (pupil periods intersecting with running periods were excluded). For each cell, we computed the difference between the dilation and constriction medians of these period-wise means. These values are the quantities plotted in figures with the axis title ‘Dilation − constriction’ (median$\,- {\overline {\Delta F/{F}_{0}}}$). Computed values were then binned into a histogram using the Python package numpy (v.1.19.5) histogram function (L5: 15 bins, Fig. 5d; VIP^+^: 20 bins, Fig. 5g).

For analyses of layer 5 pyramidal cells, only recordings that showed a significant difference in median JEDI3hyp $\,- {\overline {\Delta F/{F}_{0}}},$ during pupil dilation relative to constriction (Kruskal–Wallis test, *P* < 0.05; L5 *n* = 20/32) were used for the phase-binning analysis to follow. For VIP^+^ and SOM^+^ interneurons, an analogous test was performed using a more stringent significance threshold (*P* < 0.01; VIP^+^ *n* = 92/111, Fig. 5g and Extended Data Fig. 10; SOM^+^ *n* = 39/83, Extended Data Fig. 10). Because nearly all VIP^+^ cells were dilation selective by this test, we did not exclude any VIP^+^ cells from further analysis. SOM^+^ cells were not analyzed further.

We then iterated through all non-running pupil periods and binned the ${R}$ during each pupil period by the phase of the pupil diameter (65 phase bins from −*π* to *π*) to align all pupil periods to a single canonical cycle of dilation and constriction. Phase-binned Δ*F*/*F*_0_ values were then averaged within each bin and mean centered for each cell. For layer 5 pyramidal neurons, dilation-selective and constriction-selective cells were averaged and plotted separately, and phase-binned fluorescence was minimally smoothed with a rolling average window of 3 bins. For VIP^+^ cells, all 111 cells were averaged together without smoothing.

#### Pupil phase binning: Δ2–10-Hz Hilbert transform amplitude

To compute the pupil phase-related change in low-frequency oscillations, JEDI3hyp traces were computed from all pixels in the manually segmented masks, inverted and bandpass filtered using a zero-phase 2–10-Hz Hamming bandpass filter. The Hilbert transform was applied to these traces, and the amplitude of the transform was filtered between 0.1 Hz and 1 Hz to match the timescales on which pupil periods occur. The Hilbert transformation detects the change in the signal envelope from the 2–10-Hz filtered trace. This transformation does not require averaging over multiple recordings, thereby simplifying the analysis and enhancing the detection of frequency changes compared with wavelets or Fourier transforms. We then iterated through all non-running pupil periods and binned the amplitude measurements by the phase of the pupil diameter (65 phase bins from –*π* to *π*) to align all pupil periods to a single canonical cycle. These phase-binned fluorescence values were averaged, and error bars were computed using the s.e.m. All fluorescence pupil phase plots are the mean R ± s.e.m. for each bin. Finally, this phase-binned signal was minimally smoothed with a rolling average window of three bins and centered around 0% by subtracting the mean.

### Reporting summary

Further information on research design is available in the Nature Portfolio Reporting Summary linked to this article.

## Online content

Any methods, additional references, Nature Portfolio reporting summaries, source data, extended data, supplementary information, acknowledgements, peer review information; details of author contributions and competing interests; and statements of data and code availability are available at https://doi.org/10.1038/s41592-026-03043-8.

## Supplementary information

Supplementary Information Supplementary Note 1 and Supplementary Figs. 1–5

Reporting Summary

Peer Review File

Supplementary Video 1 Time-lapse video of JEDI3sub-Kv fluorescence dynamics. The video shows a neuron expressing JEDI3sub-Kv (left; scale bar: 6 μm) alongside a synchronized plot of fractional fluorescence changes (right), displayed at 4× slower than real time. Imaging was performed using high-speed two-photon scanning with Acousto-Optic Deflectors (AODs), and no motion correction was applied. This dataset represents a typical example from the motion artifact analysis (Extended Data Fig. 3) and corresponds to the same activity snippet shown in the zoomed-in view of Extended Data Fig. 3b.

Supplementary Video 2 Time-lapse video of JEDI3sub-Kv fluorescence dynamics shown with inverted polarity. This video is identical to Supplementary Video 1, except that the fluorescence signals have been inverted, with depolarizations appearing as brightening events, to enhance the visualization of GEVI dynamics. Accordingly, the y-axis has also been inverted to display –ΔF/F₀.

Supplementary Video 3 Motion-corrected video of JEDI3sub-Kv fluorescence dynamics imaged by FACED. Data corresponds to an 8-s-long 400-Hz recording during which eight orientations of a drifting grating were presented to the animal. The video was downsampled to 20 Hz and rendered to play at ten frames per second (fps). The video shows masked cells 17 and 18 from Extended Data Fig. 6d.

Supplementary Table 1 A table of experimental conditions for all main figures.

Supplementary Table 2 A table describing the statistics used throughout the document.

Supplementary Data 1 Source data for Supplementary Fig. 1.

Supplementary Data 2 Source data for Supplementary Fig. 3.

Supplementary Data 3 Source data for Supplementary Fig. 5.

## Source data

Source Data Fig. 1 Data in traces and graphs.

Source Data Fig. 2 Data in bar charts of Fig. 2d.

Source Data Fig. 3 Data in bar charts.

Source Data Fig. 4 Data in charts of Fig. 4f,g.

Source Data Fig. 5 Data for charts and traces.

Source Data Table 1 Values used to generate Table 1.

Source Data Extended Data Fig. 1 Values that are plotted in the graphs or charts.

Source Data Extended Data Fig. 2 Values graphed in subpanel Extended Data Fig. 2g.

Source Data Extended Data Fig. 3 Data used in graphs Extended Data Fig. 3c,d,g.

Source Data Extended Data Fig. 4 Data used in the graph in Extended Data Fig. 4g.

Source Data Extended Data Fig. 5 Data in bar charts of Extended Data Fig. 5d.

Source Data Extended Data Fig. 6 All data in graphs.

Source Data Extended Data Fig. 8 All data in graphs and traces.

Source Data Extended Data Fig. 9 All data in graphs and traces.

Source Data Extended Data Fig. 10 All data in graphs and traces.

## Data Availability

The GenBank accession numbers are https://www.ncbi.nlm.nih.gov/nuccore/PX122673 for JEDI3sub and https://www.ncbi.nlm.nih.gov/nuccore/PX122674 for JEDI3hyp. Plasmids used in this work or that are useful for other applications are available on Addgene (ID numbers 246616–246641). Raw data for main, Extended Data and Supplementary Figures have been uploaded to Zenodo (https://doi.org/10.5281/zenodo.17537897)^87^. Data that are too large to upload will be made available upon request. Source data are provided with this paper.
