# Supplementary material for: Designer indicators for two-photon recording of subthreshold voltage dynamics
Source: Nat Methods. 2026 Apr 15;23(5):986–97. doi: 10.1038/s41592-026-03043-8 (PMC13577304; doi:10.1038/s41592-026-03043-8)
Supplement: Supplementary file 1 [file 41592_2026_3043_MOESM1_ESM.pdf]

nature methods

Supplementary information

https://doi.org/10.1038/s41592-026-03043-8

Designer indicators for two-photon recording of subthreshold voltage dynamics

In the format provided by the authors and unedited

SUPPLEMENTARY NOTES

Distinguishing between genuine voltage signals and motion artefacts in GEVI recordings

To determine whether the subthreshold fluctuations recorded with JEDI3sub were free from artifacts introduced by either the scanning method or brain motion, we performed a series of analyses on data acquired using both fast 2D imaging and ULoVE.

We first acquired a reference z-stack, followed by fast 2D imaging (sampling rate: 2277 ± 271 Hz, mean ± s.d., n = 5 cells, trace corresponds to sum of all pixel intensities without motion correction) (Extended Data Figure 3A–B; Supplementary Movie 1) to track frame shifts in the x and y directions. This enabled us to reconstruct a three-dimensional representation of the fluorescent signal's displacement (Extended Data Figure 3B). Across all three spatial dimensions, shifts rarely exceeded 2.5 µm (cumulative percentile at 2.5 µm: 0.986 ± 0.014, n = 5 cells; Extended Data Figure 3C), indicating that the signal remained well-contained within the ULoVE scanning volume (e.g., Extended Data Figure 3E, *bottom left*) throughout the recording. To quantify the relationship between motion and GEVI signal, we computed the Euclidean norm of the 3D shifts over time and correlated it with the raw JEDI3sub-Kv fluorescence signal. The resulting correlation was minimal (0.033 ± 0.022, n = 5 cells; Extended Data Figure 3D), suggesting that the GEVI signal was minimally contaminated by motion artifacts.

To more directly extend these image-based findings to ULoVE microscopy, we leveraged the spatial information embedded in ULoVE recordings, which repeatedly scan a spatially extended pattern in bouts of 70 µs, to extract membrane shifts directly from the data (Extended Data Figure 3E). In this case, because the scan proceeded at 0.13 µm/µs with a 5 µs resolution, an individual 70-µs ULoVE scan consisted of 14 pixels. The cumulative distribution of membrane shifts reached 0.9964 ± 0.0095 at 2.5 µm (mean ± s.d., n = 12 cells; Extended Data Figure 3F), consistent with the fast AOD-based imaging results (Extended Data Figure 3C). The correlation between membrane displacement and ULoVE signal was negligible (0.000097 ± 0.000337, n = 12), consistent with results obtained with imaging (Extended Data Figure 3D). These results suggest that motion artifacts have a minimal impact on the recorded GEVI signals. The amplitude of subthreshold fluctuations was comparable across optical recording methods (ULoVE: 39.0 ± 9.7%, mean ± s.d., n = 12 cells; imaging: 36.7 ± 12.9%, n = 5; Extended Data Figure 3G), supporting the contention that the observed signals are subthreshold fluctuations rather than ULoVE artifacts.

To further investigate the potential impact of physiological motion —such as vessel dilation or heartbeat— we recorded slow (200 Hz) movies of bright autofluorescent structures near cortical surface vessels beneath the dura (Extended Data Figure 4A) and extracted corresponding x and y shifts (Extended Data Figure 4B). Autocorrelation analysis of these shifts revealed rhythmic components at frequencies corresponding to respiration and heartbeat (Extended Data Figure 4C). Rhythmic patterns were observed in the displacement traces extracted from both fast imaging and ULoVE recordings (Extended Data Figure 4D); these patterns were more subtle, likely due to the greater distance of these cells from blood vessels. Critically, autocorrelation analyses of the JEDI3sub-Kv fluorescence signals from both fast imaging and ULoVE recordings (Extended Data Figure 4E) revealed no such rhythmic components. Correspondingly, the Fourier power spectra of these signals showed no peaks at respiratory or cardiac frequencies (Extended Data Figure 4F). In contrast, the power spectra of the displacement traces exhibited clear peaks at the heartbeat frequency.

Furthermore, decorrelation analysis demonstrated that JEDI3sub-Kv signals were entirely independent of motion artifacts associated with breathing and heartbeat (Extended Data Figure 4G). Specifically, the weighted decorrelation time constants were reduced by 24.4- and 18.5-fold, and the asymptotic levels by 3,316- and 328,500-fold, for ULoVE and fast imaging, respectively, compared to the displacement traces.

Together, these results robustly demonstrate that JEDI3sub-Kv signals are not contaminated by brain motion artifacts across a range of timescales, for both ULoVE and fast imaging modalities.

SUPPLEMENTARY FIGURES

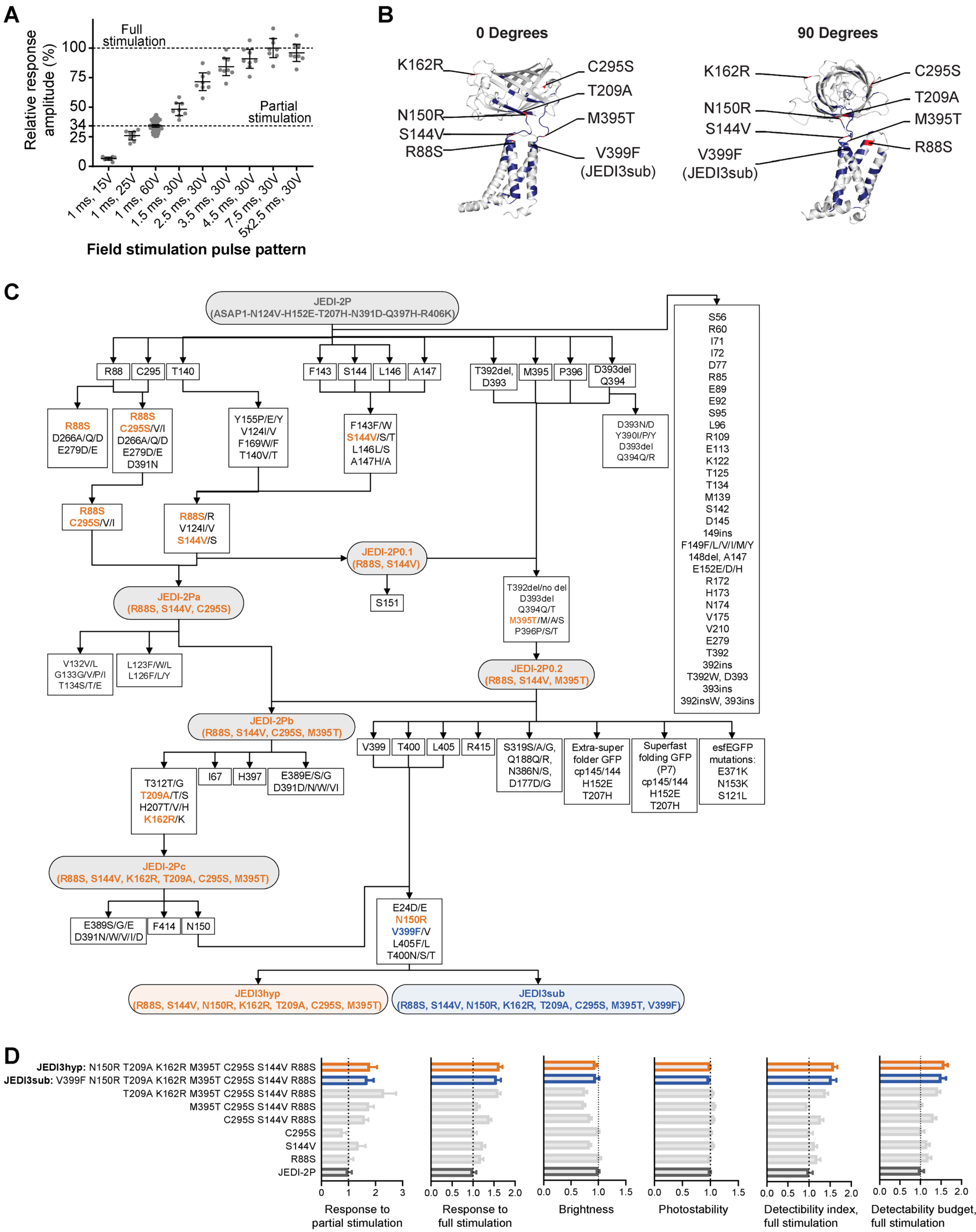

Supplementary Figure 1. 2P directed evolution of GEVIs for enhanced responses to subthreshold voltage dynamics.

(**A**) Changing electrical field stimulation strength and duration depolarizes HEK293-Kir2.1 cells to different extents, as visualized by JEDI-2P fluorescence. The partial stimulation pattern was used to screen for enhanced responses to subthresholds, while the full stimulation pattern was used to determine GEVIs' maximal responses. Data are presented as mean ± 95% CI, with 8 independent transfections. (**B**) Residues targeted during directed evolution, highlighted on a predicted 3D model. Residues mutated in JEDI3 indicators are shown in red, except for V399F, which is only found in JEDI3sub. Sites in blue were screened but did not yield mutations incorporated into the final JEDI3 sensors. (**C**) Directed evolutionary path leading to the identification of JEDI3hyp (orange mutations) and JEDI3sub (orange and blue mutations) from the parental sensor, JEDI-2P (gray). Intermediate candidates are shown in orange with a gray background. Some combination libraries included mutations from the literature or screens of other sensor lines. (**D**) Comparative performance of JEDI3sub, JEDI3hyp, and selected intermediates in our 2P multiparametric screen. Values are relative to those of JEDI-2P. The detectability index was defined as the product of response amplitude and the square root of relative brightness. The detectability budget was defined as the detectability index times the square root of the photostability. Data are presented as mean ± 95% CI, with 7 (JEDI3hyp) or 8 (other variants) independent transfections per variant.

A JEDI3hyp

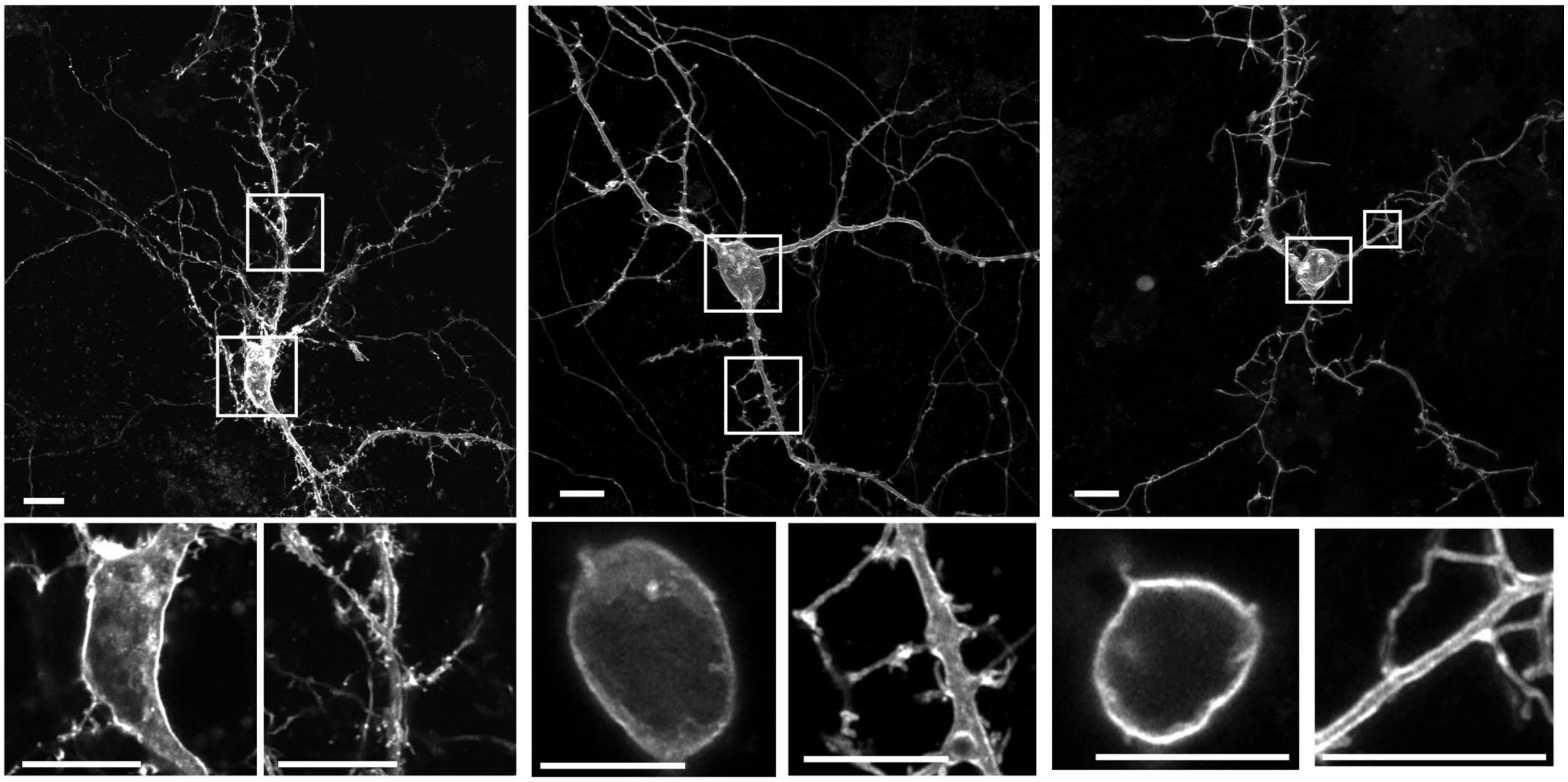

B JEDI3sub

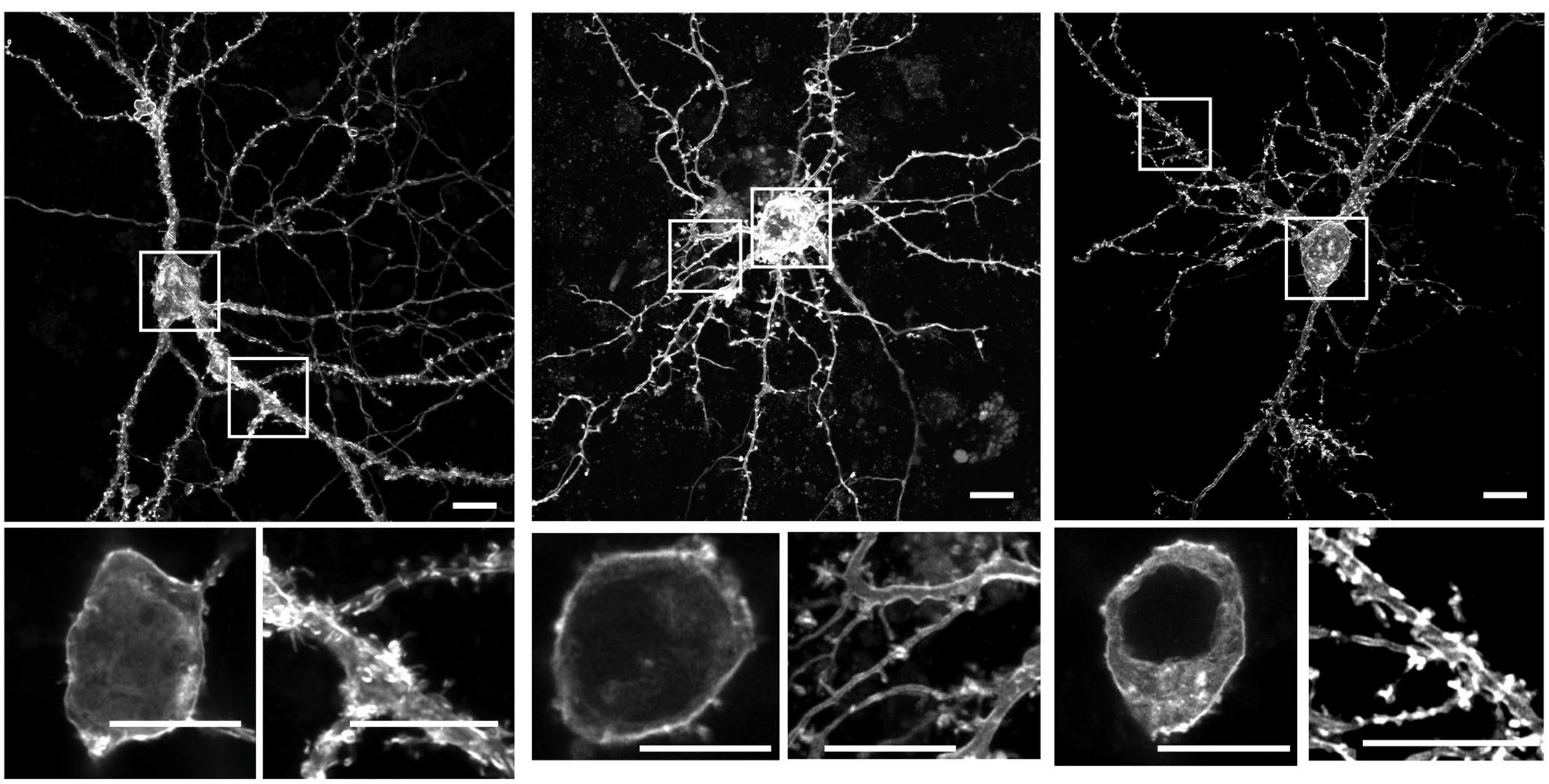

Supplementary Figure 2. JEDI3hyp and JEDI3sub indicators are expressed at the plasma membrane of cultured neurons.

Confocal microscopy images of cultured rat hippocampal neurons expressing (**A**) JEDI3hyp and (**B**) JEDI3sub. The first neuron of each indicator corresponds to the representative cell in the main figure (Figure 1B). Soma zoom-in images were taken from single confocal Z slices, while other images are confocal Z-stack maximal projections. Scale bars, 10 μm. Images were acquired from E18 neurons two days post-transfection, following 12–14 days in vitro (DIV).

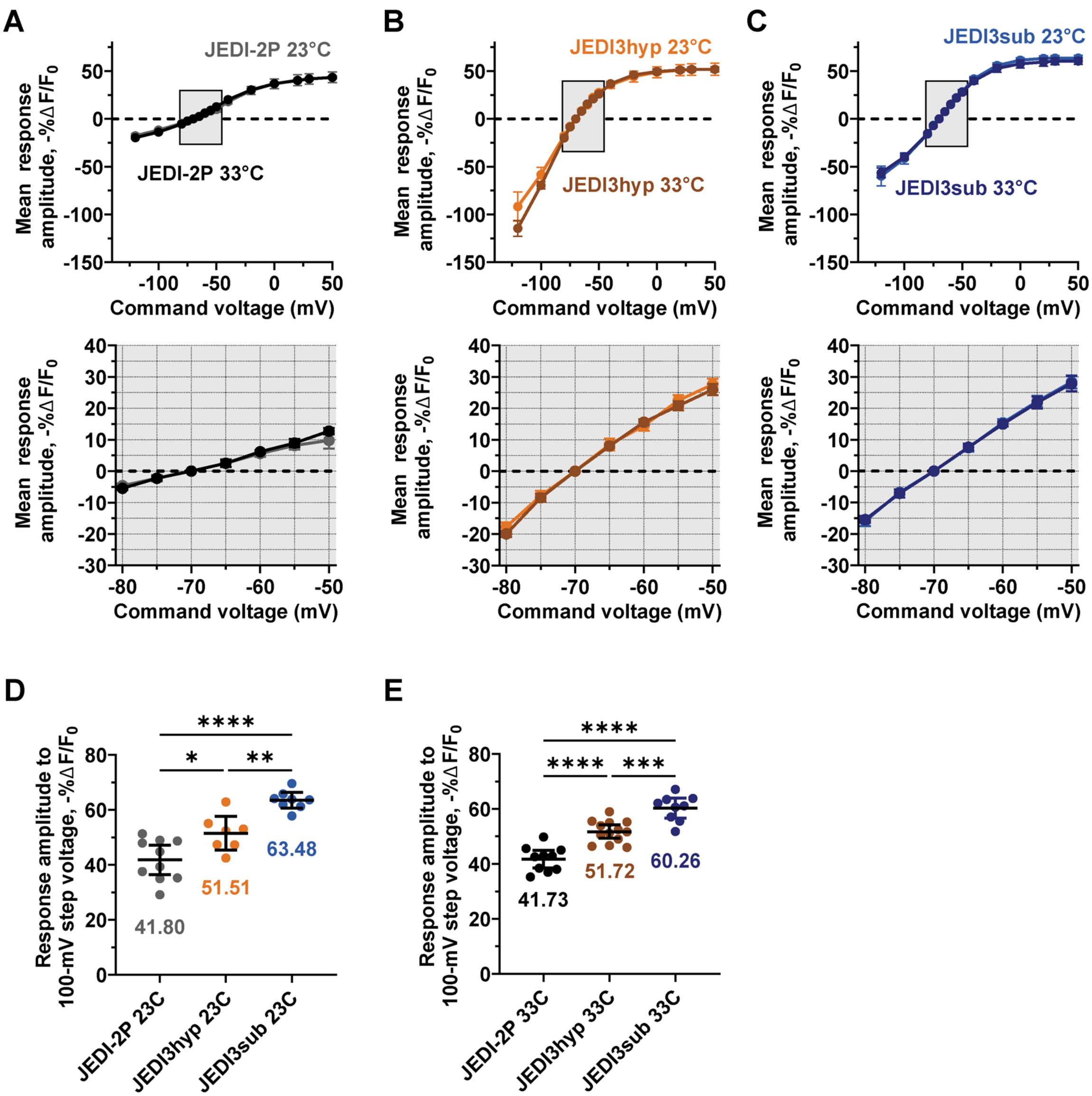

Supplementary Figure 3. Effect of temperature on the voltage sensitivity curve JEDI indicators.

(**A-C**) Mean fluorescence responses to voltage steps at ~23ºC or ~33ºC for (**A**) JEDI-2P, (**B**) JEDI3hyp, and (**C**) JEDI3sub. The bottom row shows additional quantification of smaller voltage steps around subthreshold levels. Room temperature (~23ºC) data: n = 7-10 HEK293 cells/sensor (same cells as in Figure 1H). 33ºC data (32-33ºC): n = 10 (JEDI-2P), 13 (JEDI3hyp) and 9 (JEDI3sub). JEDI3sub produced larger peak steady-state responses to 100-mV voltage steps from a resting potential of −70 mV compared with JEDI-2P and JEDI3hyp sensors at (**D**) 23ºC and (**E**) 33ºC. One-way ANOVA followed by Tukey multiple comparisons test was performed. *$p < 0.05$, **$p < 0.01$, ***$p < 0.001$, ****$p < 0.0001$ (Supplementary Table 2).

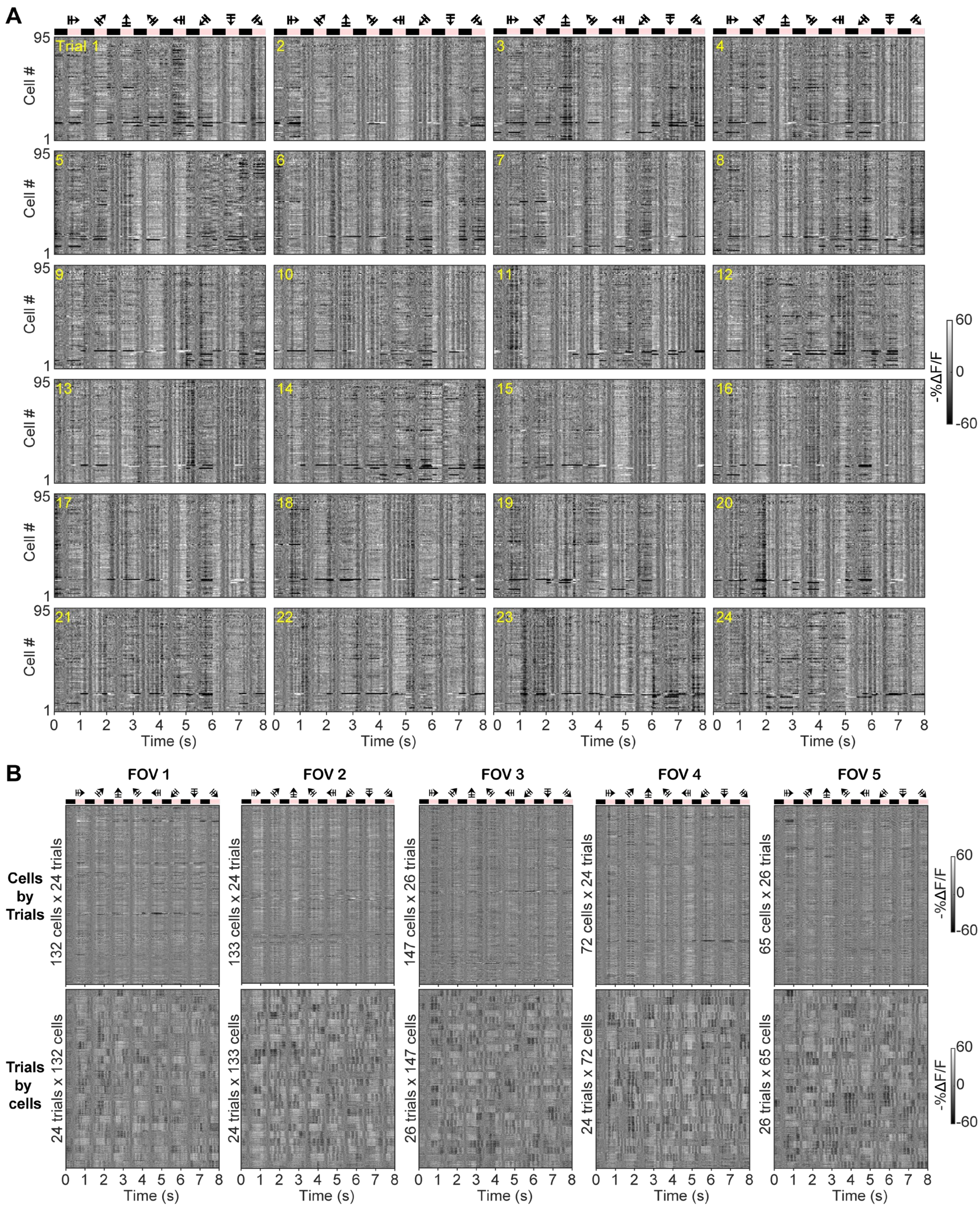

Supplementary Figure 4. JEDI3sub reports subthreshold activity of groups of cells over multiple visual stimulation trials.

(**A**) Subthreshold response traces from 95 cells across all 24 trials (same dataset as the main figure). (**B**) Five additional example fields-of-view from two separate mice showing single-trial subthreshold traces grouped by cells (top) or trial (*bottom*). FOV 1-3 (320 × 400 µm) were acquired at 400 Hz, while FOV 4-5 (160 × 400 µm) were acquired at 769 Hz.

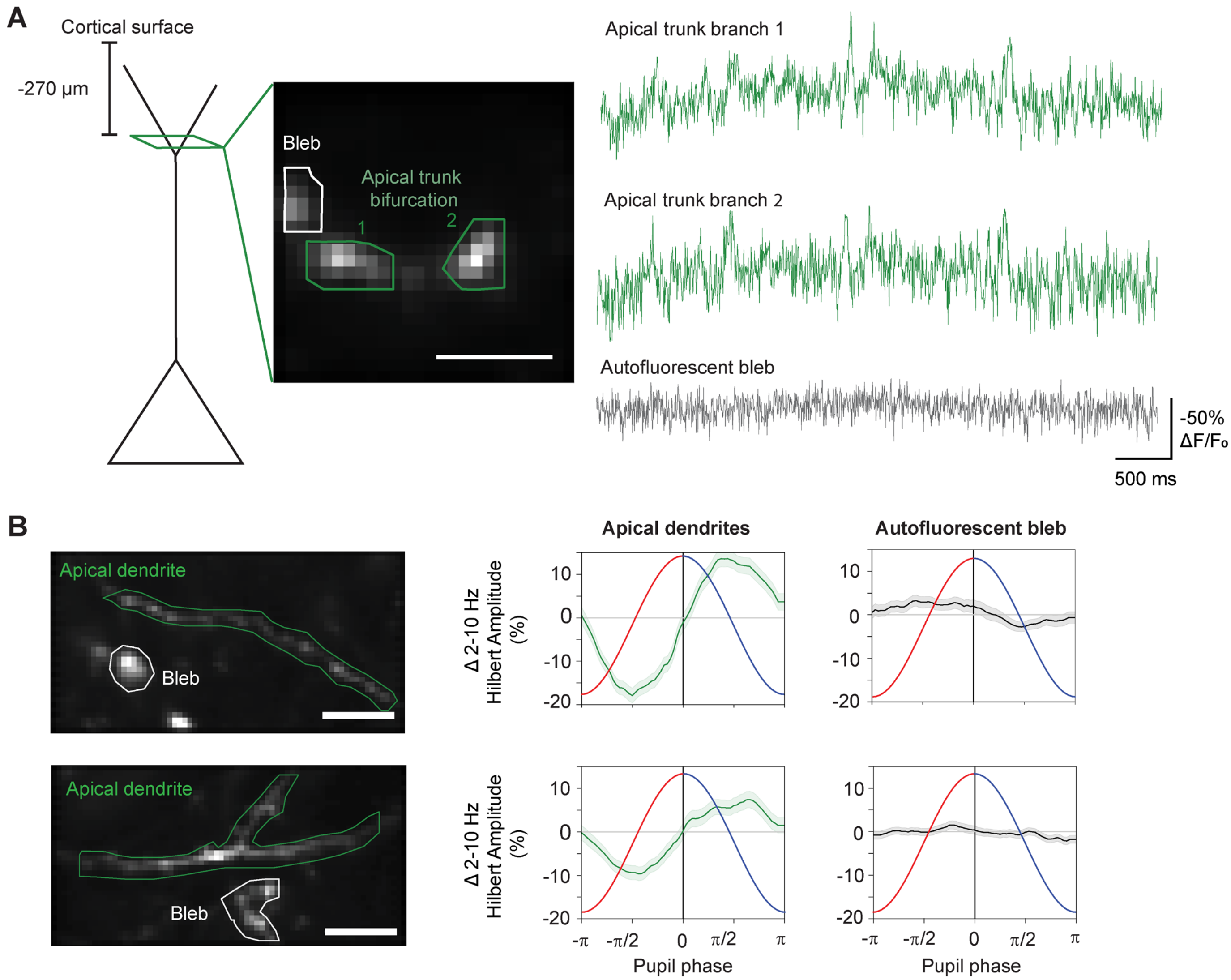

Supplementary Figure 5. JEDI3hyp can report genuine subthreshold changes in dendritic segments.

(**A**) Fluorescence dynamics in a bifurcating apical dendrite. Dendritic segments are green, and the adjacent autofluorescent bleb (gray) serves as a non-voltage-sensitive control. Scale bar, 10 μm. (**B**) *Left*: Images of JEDI3hyp-expressing apical dendrites (outlined in green) from two distinct Layer 5 pyramidal neurons. Autofluorescent blebs (outlined in white), which serve as non-voltage-sensitive controls, are also shown. *Right*: Pupil phase-dependent changes in 2–10 Hz Hilbert amplitude are observed in dendritic segments, but not in the autofluorescent blebs.
