# Supplementary material for: Designer indicators for two-photon recording of subthreshold voltage dynamics
Source: Nat Methods. 2026 Apr 15;23(5):986–97. doi: 10.1038/s41592-026-03043-8 (PMC13577304; doi:10.1038/s41592-026-03043-8)
Supplement: Supplementary file 2 [file 41592_2026_3043_MOESM2_ESM.pdf]

nature portfolio

Corresponding author(s): St-Pierre, Francois

Last updated by author(s): Nov 10, 2025

Reporting Summary

Nature Portfolio wishes to improve the reproducibility of the work that we publish. This form provides structure for consistency and transparency in reporting. For further information on Nature Portfolio policies, see our Editorial Policies and the Editorial Policy Checklist.

Statistics

For all statistical analyses, confirm that the following items are present in the figure legend, table legend, main text, or Methods section.

n/a	Confirmed	
☐	☒	The exact sample size (*n*) for each experimental group/condition, given as a discrete number and unit of measurement
☐	☒	A statement on whether measurements were taken from distinct samples or whether the same sample was measured repeatedly
☐	☒	The statistical test(s) used AND whether they are one- or two-sided<br>*Only common tests should be described solely by name; describe more complex techniques in the Methods section.*
☒	☐	A description of all covariates tested
☐	☒	A description of any assumptions or corrections, such as tests of normality and adjustment for multiple comparisons
☐	☒	A full description of the statistical parameters including central tendency (e.g. means) or other basic estimates (e.g. regression coefficient) AND variation (e.g. standard deviation) or associated estimates of uncertainty (e.g. confidence intervals)
☐	☒	For null hypothesis testing, the test statistic (e.g. *F*, *t*, *r*) with confidence intervals, effect sizes, degrees of freedom and *P* value noted<br>*Give P values as exact values whenever suitable.*
☒	☐	For Bayesian analysis, information on the choice of priors and Markov chain Monte Carlo settings
☒	☐	For hierarchical and complex designs, identification of the appropriate level for tests and full reporting of outcomes
☐	☒	Estimates of effect sizes (e.g. Cohen's *d*, Pearson's *r*), indicating how they were calculated

Our web collection on statistics for biologists contains articles on many of the points above.

Software and code

Policy information about availability of computer code

Data collection	The software used for data collection is mentioned in Methods, and varied between the various assays and Teams. It includes: NIS-Elements AR 4.6, MultiClamp 700B, Clampex 11.1, Zen 2.3 SP1 FP3 (black edition), and MATLAB2021a. For additional software or programs used, please see the methods section of the paper.
Data analysis	The software used for data analysis is mentioned in Methods. It includes Zen 2.3 (blue edition), GraphPad Prism 9, MATLAB2021b, MATLAB2022a, and MATLAB2022b, Python. For additional software or programs used, please see the methods section of the paper.

For manuscripts utilizing custom algorithms or software that are central to the research but not yet described in published literature, software must be made available to editors and reviewers. We strongly encourage code deposition in a community repository (e.g. GitHub). See the Nature Portfolio guidelines for submitting code & software for further information.

Data

Policy information about availability of data

All manuscripts must include a data availability statement. This statement should provide the following information, where applicable:

- Accession codes, unique identifiers, or web links for publicly available datasets
- A description of any restrictions on data availability
- For clinical datasets or third party data, please ensure that the statement adheres to our policy

All raw data generated in this study is available upon request. The GeneBank accession numbers are PX122673 for JEDI3sub and PX122674 for JEDI3hyp. Plasmids

used in this work or that are useful for other applications are available on Addgene (ID numbers 246616 through 246641). Most raw data has been uploaded to Zenodo86 (10.5281/zenodo.17537897). For data too large to upload, the data will be available upon request.

Research involving human participants, their data, or biological material

Policy information about studies with human participants or human data. See also policy information about sex, gender (identity/presentation), and sexual orientation and race, ethnicity and racism.

Reporting on sex and gender	N/A
Reporting on race, ethnicity, or other socially relevant groupings	N/A
Population characteristics	N/A
Recruitment	N/A
Ethics oversight	N/A

Note that full information on the approval of the study protocol must also be provided in the manuscript.

Field-specific reporting

Please select the one below that is the best fit for your research. If you are not sure, read the appropriate sections before making your selection.

☒ Life sciences ☐ Behavioural & social sciences ☐ Ecological, evolutionary & environmental sciences

For a reference copy of the document with all sections, see nature.com/documents/nr-reporting-summary-flat.pdf

Life sciences study design

All studies must disclose on these points even when the disclosure is negative.

Sample size	Sample sizes were set based on experimental variability observed in published work with similar experiments (e.g., https://doi.org/10.1016/j.cell.2022.07.013), as recommended by the NIH guidelines. Statistical analyses to determine optimal sample sizes were not conducted.
Data exclusions	Data exclusion criteria are discussed in the Methods section.
Replication	The value and definition of the sample size is provided in figure captions. To ensure reproducibility, four different groups validated the sensors across different microscopy technique and experimental paradigms.
Randomization	Randomization does not apply to our study.
Blinding	Blinding was not necessary because the collection and analysis steps that could be subject to bias were performed using pre-defined automated methods. For example, while cell selection for ULoVE experiments was not blinded, variables that could be subject to bias (brightness and SNR) were not evaluated. Response amplitude and photostability were quantified using automated methods after cell selection, and are thus not subject to bias.

Reporting for specific materials, systems and methods

We require information from authors about some types of materials, experimental systems and methods used in many studies. Here, indicate whether each material, system or method listed is relevant to your study. If you are not sure if a list item applies to your research, read the appropriate section before selecting a response.

Materials & experimental systems

n/a	Involved in the study	
☒	☐	Antibodies
☐	☒	Eukaryotic cell lines
☒	☐	Palaeontology and archaeology
☐	☒	Animals and other organisms
☒	☐	Clinical data
☒	☐	Dual use research of concern
☒	☐	Plants

Methods

n/a	Involved in the study	
☒	☐	ChIP-seq
☒	☐	Flow cytometry
☒	☐	MRI-based neuroimaging

Eukaryotic cell lines

Policy information about cell lines and Sex and Gender in Research

Cell line source(s)	The HEK293A cell line was obtained from Thermo Fisher. The HEK293-Kir2.1 cell line was a gift from Gui-Rong Li Lab, who originally made this cell line; there is no commercial resource for this cell line.
Authentication	The HEK293A cell line and HEK293-Kir2.1 cell line were authenticated by STR profiling at the Cytogenetics and Cell Authentication Core at MD Anderson Cancer Center.
Mycoplasma contamination	The HEK293A and HEK293-Kir2.1 cell lines were tested negative for micoplasma contamination by Micoplasma Detection kit (Lonza).
Commonly misidentified lines (See ICLAC register)	No commonly misidentified lines were used.

Animals and other research organisms

Policy information about studies involving animals; ARRIVE guidelines recommended for reporting animal research, and Sex and Gender in Research

Laboratory animals	Described in methods, for each of the four labs who conducted in vivo experiments.
Wild animals	This study did not involve wild animals.
Reporting on sex	In vivo figures were either just male or included both males and females, as described in the Methods section.
Field-collected samples	No samples were collected from the field.
Ethics oversight	Our research complies with all relevant ethical regulations. All procedures involving experimental animals were conducted in strict accordance with the National Institute of Health guidelines and were approved by the corresponding Animal Care and Use Committee of each institution where animal experiments were performed.

Note that full information on the approval of the study protocol must also be provided in the manuscript.

Plants

Seed stocks	N/A
Novel plant genotypes	*Describe the methods by which all novel plant genotypes were produced. This includes those generated by transgenic approaches, gene editing, chemical/radiation-based mutagenesis and hybridization. For transgenic lines, describe the transformation method, the number of independent lines analyzed and the generation upon which experiments were performed. For gene-edited lines, describe the editor used, the endogenous sequence targeted for editing, the targeting guide RNA sequence (if applicable) and how the editor was applied.*
Authentication	*Describe any authentication procedures for each seed stock used or novel genotype generated. Describe any experiments used to assess the effect of a mutation and, where applicable, how potential secondary effects (e.g. second site T-DNA insertions, mosiacism, off-target gene editing) were examined.*
