# Supplementary material for: Designer indicators for two-photon recording of subthreshold voltage dynamics
Source: Nat Methods. 2026 Apr 15;23(5):986–97. doi: 10.1038/s41592-026-03043-8 (PMC13577304; doi:10.1038/s41592-026-03043-8)
Supplement: Supplementary file 3 [file 41592_2026_3043_MOESM3_ESM.pdf]

nature portfolio

Peer Review File

Designer indicators for two-photon recording of subthreshold voltage dynamics

Corresponding Author: Dr Francois St-Pierre

This file contains all editorial decision letters in order by version, followed by all author rebuttals in order by version.

Version 0:

Decision Letter:

24th Apr 2025

Dear Francois,

Thank you for your patience. Your Article, "Designer indicators for two-photon recording of subthreshold voltage dynamics", has now been seen by two reviewers. As you will see from their comments below, although the reviewers find your work of considerable potential interest, they have raised a number of concerns. We are interested in the possibility of publishing your paper in Nature Methods, but would like to consider your response to these concerns before we reach a final decision on publication.

We therefore invite you to revise your manuscript to address these concerns. Specifically, we think stronger arguments for subthreshold activity vs. noise are needed (I understand that obtaining in vivo electrophysiology data in combination with voltage imaging as ground truth is prohibitively difficult).

We are committed to providing a fair and constructive peer-review process. Do not hesitate to contact us if there are specific requests from the reviewers that you believe are technically impossible or unlikely to yield a meaningful outcome.

When revising your paper:

* include a point-by-point response to the reviewers and to any editorial suggestions

* please underline/highlight any additions to the text or areas with other significant changes to facilitate review of the revised manuscript

* address the points listed described below to conform to our open science requirements

* ensure it complies with our general format requirements as set out in our guide to authors at www.nature.com/naturemethods

* resubmit all the necessary files electronically by using the link below to access your home page

Link Redacted

Note: This URL links to your confidential home page and associated information about manuscripts you may have submitted, or that you are reviewing for us. If you wish to forward this email to co-authors, please delete the link to your homepage.

We hope to receive your revised paper within 2-3 months. If you cannot send it within this time, please let us know. In this event, we will still be happy to reconsider your paper at a later date so long as nothing similar has been accepted for publication at Nature Methods or published elsewhere.

OPEN SCIENCE REQUIREMENTS

REPORTING SUMMARY AND EDITORIAL POLICY CHECKLISTS
When revising your manuscript, please update your reporting summary and editorial policy checklists.

Reporting summary: https://www.nature.com/documents/nr-reporting-summary.zip
Editorial policy checklist: https://www.nature.com/documents/nr-editorial-policy-checklist.zip

If your paper includes custom software, we also ask you to complete a supplemental reporting summary.

Software supplement: https://www.nature.com/documents/nr-software-policy.pdf

Please submit these with your revised manuscript. They will be available to reviewers to aid in their evaluation if the paper is re-reviewed. If you have any questions about the checklist, please see http://www.nature.com/authors/policies/availability.html or contact me.

Please note that these forms are dynamic 'smart pdfs' and must therefore be downloaded and completed in Adobe Reader. We will then flatten them for ease of use by the reviewers. If you would like to reference the guidance text as you complete the template, please access these flattened versions at http://www.nature.com/authors/policies/availability.html.

Finally, please ensure that you retain unprocessed data and metadata files after publication, ideally archiving data in perpetuity, as these may be requested during the peer review and production process or after publication if any issues arise.

EXTENDED DATA FIGURES
When re-submitting your manuscript, please ensure that any supplementary figures and tables that are crucial to the manuscript's conclusions are converted into Extended Data figures and tables to increase visibility of these data. Extended Data figures and tables are online-only (present in the online PDF and full-text HTML versions of the paper), peer-reviewed display items that provide essential background to the article but are not included in the main article due to space constraints. A maximum of ten Extended Data display items (figures and tables) is permitted.

DATA AVAILABILITY
We strongly encourage you to deposit all new data associated with the paper in a persistent repository where they can be freely and enduringly accessed. We recommend submitting the data to discipline-specific and community-recognized repositories; a list of repositories is provided here: http://www.nature.com/sdata/policies/repositories

All novel DNA and RNA sequencing data, protein sequences, genetic polymorphisms, linked genotype and phenotype data, gene expression data, macromolecular structures, and proteomics data must be deposited in a publicly accessible database, and accession codes and associated hyperlinks must be provided in the "Data Availability" section.

Refer to our data policies here: https://www.nature.com/nature-research/editorial-policies/reporting-standards#availability-of-data

To further increase transparency, we encourage you to provide, in tabular form, the data underlying the graphical representations used in your figures. This is in addition to our data-deposition policy for specific types of experiments and large datasets. For readers, the source data will be made accessible directly from the figure legend. Spreadsheets can be submitted in .xls, .xlsx or .csv formats. Only one (1) file per figure is permitted: thus if there is a multi-paneled figure the source data for each panel should be clearly labeled in the csv/Excel file; alternately the data for a figure can be included in multiple, clearly labeled sheets in an Excel file. File sizes of up to 30 MB are permitted. When submitting source data files with your manuscript please select the Source Data file type and use the Title field in the File Description tab to indicate which figure the source data pertains to.

Please include a "Data availability" subsection in the Online Methods. This section should inform readers about the availability of the data used to support the conclusions of your study, including accession codes to public repositories, references to source data that may be published alongside the paper, unique identifiers such as URLs to data repository entries, or data set DOIs, and any other statement about data availability. At a minimum, you should include the following statement: "The data that support the findings of this study are available from the corresponding author upon request", describing which data is available upon request and mentioning any restrictions on availability. If DOIs are provided, please include these in the Reference list (authors, title, publisher (repository name), identifier, year). For more guidance on how to write this section please see: http://www.nature.com/authors/policies/data/data-availability-statements-data-citations.pdf

MATERIALS AVAILABILITY
As a condition of publication in Nature Methods, authors are required to make unique materials promptly available to others without undue qualifications.

Authors reporting new chemical compounds must provide chemical structure, synthesis and characterization details. Authors

reporting mutant strains and cell lines are strongly encouraged to use established public repositories.

More details about our materials availability policy can be found at https://www.nature.com/nature-portfolio/editorial-policies/reporting-standards#availability-of-materials

SUPPLEMENTARY PROTOCOL
To help facilitate reproducibility and uptake of your method, we ask you to prepare a step-by-step Supplementary Protocol for the method described in this paper. We encourage authors to share their step-by-step experimental protocols on a protocol sharing platform of their choice and report the protocol DOI in the reference list. Nature Portfolio's protocols.io is a free-to-use and open resource for protocols; protocols deposited onto protocols.io are citable and can be linked from the published article. More details can found at protocols.io.

ORCID
Nature Methods is committed to improving transparency in authorship. As part of our efforts in this direction, we are now requesting that all authors identified as ‘corresponding author’ on published papers create and link their Open Researcher and Contributor Identifier (ORCID) with their account on the Manuscript Tracking System (MTS), prior to acceptance. This applies to primary research papers only. ORCID helps the scientific community achieve unambiguous attribution of all scholarly contributions. You can create and link your ORCID from the home page of the MTS by clicking on ‘Modify my Springer Nature account’. For more information please visit please visit www.springernature.com/orcid.

Please do not hesitate to contact me if you have any questions or would like to discuss these revisions further. We look forward to seeing the revised manuscript and thank you for the opportunity to consider your work.

Best regards,
Nina

Nina Vogt, PhD
Senior Editor
Nature Methods

Reviewers' Comments:

Reviewer #2 (Remarks to the Author):

Land et al. focus on advancing genetically encoded voltage indicators (GEVIs). In their manuscript, they argue that existing GEVIs lack sufficient sensitivity for detecting millivolt-scale subthreshold signals. To address this, they have refined a multiparametric two-photon high-throughput screening platform to develop two novel indicators—JEDI3sub and JEDI3hyp—designed for subthreshold voltage detection in both neuronal cell bodies and dendrites. Using fast two-photon (2P) optical recordings in awake, behaving mice, the authors report that the new JEDI3 variants outperform the earlier JEDI-2P.

Point 1
Comparison with Prior Studies.
A recent Neuron paper (2024) by Dr. M. Z. Lin—St-Pierre’s former postdoctoral advisor—introduced ASAP5, a novel GEVI capable of fast 2P optical recording in live mice. Notably, ASAP5 was demonstrated to record subthreshold membrane potential transients in dendrites of cultured cells. There is a significant overlap between the current study and the findings reported by Hao et al. (2024), as outlined below:
[A] Two-Photon Microscopy: Land et al. claim that their new GEVI variant is optimized for deep-tissue recording using 2P microscopy. However, Hao et al. (2024) have already established that ASAP5 performs well under similar imaging conditions.
[B] Subthreshold Voltage Detection: While Land et al. emphasize that their GEVI is tailored specifically for subthreshold voltage detection, Hao et al. (2024) demonstrated that ASAP5 effectively detected miniature excitatory postsynaptic potentials (“minis”).
[C] Dendritic Recordings: Land et al. highlight breakthrough recordings of voltage changes in fine dendritic structures. Yet, recordings of voltage fluctuations in fine dendrites were also presented in Hao et al. (2024).
Given these overlaps, it is concerning that Hao et al. (2024) is not included in the reference list. Its relegation to the Methods section appears to minimize its prominence, and does not adequately acknowledge its contribution.
It is recommended that Hao et al. (2024) be cited and discussed in the Introduction to clarify how the current work advances the field. A similar approach should be taken with respect to Xie et al. (2021), by explaining in both the Introduction and Discussion how Land et al.’s findings build upon and differ from these earlier studies.
In addition to Hao et al. (2024) and Xie et al. (2021), several other key publications have contributed to the field of dendritic voltage imaging but were not referenced by Land et al. These include:
- Adam E. Cohen’s work on Voltron recordings in dendrites (presented at conferences and available on BioRxiv), which explores dendritic excitations.
- Dorgans et al. (2020), who imaged subthreshold voltage oscillations with cellular resolution in the inferior olive in vitro (Front.

Cell. Neurosci.).
- Jin et al. (2012), which reported imaging of subthreshold events in neurons using a fluorescent protein voltage probe (Neuron).
- Berger et al. (2007), combining voltage and calcium epifluorescence imaging to reveal both subthreshold and suprathreshold dynamics in mouse barrel cortex (J. Neurophysiol.).
- Djurisic et al. (2004), which detailed voltage imaging of EPSPs from dendrites of mitral cells (J. Neurosci.).

Point 2
Interpretation of Optical Traces (Lines 202 & Figures 2B and 3).
The manuscript asserts that “these results demonstrate JEDI3sub’s capacity to track subthreshold activity across large neuronal populations, enabling investigation into population-level information processing.” However, the data presented in Fig. 3 appear to be derived from noisy optical traces whose undulations may be artifactual. Even the best recordings (Fig. 2B) do not unequivocally differentiate subthreshold depolarizations from scanning or mechanical artifacts (cardiovascular, breathing, etc.). Without concurrent whole-cell recordings—as exemplified by Reimer et al. (2014)—it is challenging to confirm that the observed signals represent genuine subthreshold events. Note that Reimer is on the author list of the current manuscript.

Point 3
Replication of Electrophysiological Findings (Line 252 and Fig. 5).
The statement that “Using 2P voltage imaging with resonant-scanning microscopy, we confirmed that JEDI3hyp-Kv replicated these electrophysiological findings...” is not convincingly supported. In Fig. 5B, the optical traces are difficult to interpret without additional physiological data, making it unclear whether the observed undulations reflect meaningful electrophysiological events.

Point 4
Dendritic Recordings (Figure 5E):
The authors claim to record dendritic potentials in the terminal branches of cortical pyramidal cells. However, a closer inspection of Fig. 5E reveals an absence of such convincing recordings. This raises concerns about the method’s reliability for capturing dendritic subthreshold depolarizations.

Point 5
Missing Dendritic Plateau Potentials.
The manuscript does not report the robust glutamate-mediated dendritic plateau potentials described by Kerlin et al. (2019). This omission may reflect limitations in the ability of the JEDI3 variants to accurately track dendritic voltage dynamics.

Point 6
Terminology: “Population Voltage Imaging” vs. “Multi-Cell Imaging”.
The term “population voltage imaging” was erroneously used by biochemists and protein designers. The fact that biochemists and molecular cell biologists focused on making new GEVIs (Ed Boyden, Adam Cohen, and others) had erroneously dubbed their multi-cell recordings as “population imaging” (e.g., Piatkevich et al. 2019), does not warrant a mindless propagation of this erroneous terminology.
The current authors erroneously refer to their approach as “population voltage imaging” (line 670). Traditionally, this term is reserved for techniques that record an aggregate response from many neurons (e.g., wide-field imaging). In population imaging experiments, activity of many elements is mixed into one representative signal; an aggregate signal that represents a given population. Furthermore, the term “population voltage imaging” is a well-established terminology in neuroscience, by pioneers of voltage imaging including Brian M. Salzberg, Amiram Grinvald, Carl Petersen, and others. For further readings, do check: Momose-Sato and Sato 2019; Storace et al. 2019; Short and Wachowiak 2019; Baker et al. 2015; Allen et al. 2017; Pinto et al. 2019.
In contrast, methods that record individual neurons are better described as “multi-cell imaging”. The authors should revise their terminology accordingly to prevent confusion ensuring that the terms accurately reflect the spatial resolution and methodological approach of their study.

Point 7
Membrane Localization (Line 107)
The manuscript states,
“Confocal microscopy revealed robust plasma membrane localization in the soma and dendrites of dissociated rat hippocampal neurons for both JEDI3 variants (Fig. 1B).”
Relying on a single example (Fig. 1B) is insufficient to substantiate a claim of “robust” membrane localization. In scientific reporting, strong claims require comprehensive and reproducible experimental evidence—ideally supported by rigorous statistical analysis that demonstrates resistance to noise and outliers. The authors should expand their dataset and clearly describe the statistical methods used to validate membrane localization.

Point 8
Specification of Physical Units and Methodological References.
Throughout the Methods, critical parameters are described without specifying physical units or citing established protocols. For example, in line 398 the “detectability index” is defined as the response amplitude (units not provided) multiplied by the square root of the relative brightness (units not provided). A reference detailing the procedure for these measurements is also missing.
The authors must:
Clearly state the physical units for all quantitative parameters.
Provide a citation or detailed description of the adopted procedures, particularly for parameters like photostability and

detectability.

Point 9
Calcium Indicators and Subthreshold Activity (Line 55).
The manuscript states that “because GECIs detect calcium influx associated with APs, they generally cannot report subthreshold activity.” This statement is inaccurate, as recent evidence indicates that calcium indicators can, in fact, detect subthreshold neuronal activity (Milicevic et al., 2024, Sci. Rep., PMID: 39367010).

Point 10
Clarity in Biological Preparations (Fig. 1)
Figure 1 presents data from different biological preparations (cortical neurons and HEK cells) without clear labeling. For example, the caption for Fig. 1D and the pyramidal neuron image in Fig. 1B suggest that all data are neuron-specific, which is misleading. Similarly, in panel G the description—“JEDI3 indicators produced larger steady-state responses…” with data from 7–10 cells per variant—appears alongside a neuron image, potentially overstating its biological relevance.
Clearly label panels that display HEK cell data (e.g., include “HEK cell” as a label).

Point 11
For Fig. 1I, the caption “Traces are the mean responses” is inadequate because:
1. The meaning of the combined “faint” and “darker” traces is not explained.
2. The number of traces used to generate the mean is not provided.

Point 12
Fig. 2. (C) caption: “Mean optical spike waveforms from the recordings shown in panel (B)”. This caption is below a usual level of standard on two levels: First, traces in panel (B) contain AP-singlets, AP-doublets, and AP-triplets. Apparently, the authors cherry-picked some APs which they wished to include into the mean trace, or they did something else, which must be explained. Second, the sample size (i.e., the number of traces) used to compute the mean is omitted. The authors want to publish this result in Nature Methods without stating explicitly the number of traces used to generate the mean trace. The authors should provide a detailed explanation of how these AP were selected for inclusion into the mean and how averages were computed, and state the corresponding sample sizes (e.g., “n = XX traces”).

Point 13
Quantification of Subthreshold Responses (Line 155)
The sentence,
“Suprathreshold (AP) response amplitudes were derived from spike-triggered averages (Fig. 2C), while subthreshold responses were quantified as the range of the low pass-filtered trace,”
is ambiguous for several reasons:
1. Definition of “Range”: The term “range” is used without a clear definition. The authors should explain how “range” is calculated in this context.
2. Visual Representation: It is unclear where the subthreshold responses are shown in Fig. 2, and no visual markers (e.g., arrows or labels) are used to highlight these features.
3. Baseline Ambiguity: A closer look at Fig. 1B suggests that the baseline voltage—where subthreshold events begin—is not well defined due to high-frequency noise and low-frequency fluctuations. This uncertainty casts doubt on the reliability of the subthreshold amplitude measurements.
A more robust method for baseline determination and clear labeling of subthreshold events is necessary.

Point 14
Discrepancy Between Quantified Data and Representative Traces
The reported quantifications indicate that, for JEDI3sub, the mean subthreshold amplitude (40% dF/F) is approximately twice the mean spike amplitude (20% dF/F) (Figs. 2E and 2F). This would imply that in real traces, APs should appear as small, rapid transients superimposed on large subthreshold “hills.” However, the representative trace in Fig. 1C shows an approximately 1:1 ratio between AP amplitude and subthreshold depolarization. Such a discrepancy between quantification and representative data undermines the reliability of the reported results. The authors must reconcile these differences.

Point 15
Photobleaching (Line 191)
The authors report that “JEDI3sub exhibited minimal photobleaching during ~200-second imaging sessions (Fig. S3B).” However, since the methods indicate the use of “photobleaching-corrected fluorescent traces,” it is unclear whether Fig. S3B represents raw or corrected data. Furthermore, photobleaching is already quantified in Fig. 1F, raising questions about the rationale for its re-presentation. The authors should clarify the data processing and the purpose of presenting photobleaching data in multiple sections.

Point 16
Simultaneous monitoring (Lines 189 and 194)
Population Imaging (Line 189): The claim that “line scanning [enabled] the capture of voltage activity across large populations of neurons” is vague. The authors should specify the average number of neurons monitored simultaneously (including standard deviation) in the experiments that support Fig. 3.

Point 17
Cell Selection (Line 194): Although data were acquired from thousands of cells, only 95 were analyzed. The criteria for cell

inclusion must be explicitly stated. Furthermore, comparing the activity of 95 selected cells with data from thousands of cells (as in reference 29) is not valid without a clear justification for such a limited analysis.

Point 18
Errors
Imaging Solution (Line 377): The statement “The imaging solution was the same, except that the final HEPES concentration was increased to 20 mM” is ambiguous. It should clarify whether this is compared to the formulation in XXXX (201X) and specify the initial concentration.
Stimulation Parameters (Line 386): There is a typographical error in the description of stimulation parameters (e.g., “25 V for 1 ms, 30 V for, 30 V for 2.5 ms, …”). The authors need to carefully review and correct these details.
Typographical Errors (Line 395): The sentence, “If there was more than 1 well per variant on a plate each well was then be averaged together,” contains a grammatical error. It should read, “If more than one well per variant was present on a plate, the data from each well were averaged together.”

Point 19
Physical Units: Parameters such as the “detectability budget” in line 399 are defined without physical units.
Photostability: There is no definition or quantification of photostability, nor a reference to the procedure used.

Point 20
Background Correction and AUC Calculation (Line 514): The method for calculating the area under the curve (AUC) lacks a description of the time window used for the calculation. More comprehensive details are needed to ensure reproducibility.

Point 21
Cell Transfection (Line 423):
The statement that “HEK293-Kir2.1 cells were not transfected and plated the day before patching” requires further explanation. The significance of this methodological choice should be discussed.

Reviewer #3 (Remarks to the Author):

This paper introduces two new genetically expressed voltage indicators (GEVIs), which were optimized from their precursor JEDI-2P using a targeted screen and rationale design. The aim was to overcome limitations in optical detection and characterization of subthreshold membrane potential (Vm) changes. This goal is important because Vm changes are essential for neuronal integration and neural population dynamics. The development and characterization of two selected JEDI3s (‘sub’ and ‘hyp’) is well presented, following closely up on the previous report of JEDI-2P. In addition, a thorough comparison of JEDI3sub and JEDI3hyp to JEDI-2P is provided both in vitro and in vivo. A rudimentary comparison to ASAP5 is mentioned in the text but no example traces are provided. In the remaining paper, proof-of-concept data are given for three example applications methods (in vivo population imaging, SWR-related PV-interneuron activity, and brain-state related oscillation changes) using state-of-the-art fast 2P imaging. All three examples clearly demonstrate the capability of the new JEDI3s to provide useful subthreshold Vm information.

Overall, I find this an excellent manuscript, well-written, quantitative, and with good demonstrations of the new JEDI3 indicator subtypes. It is a significant tool optimization and I expect that it will have an impact on the field as many labs are awaiting exactly such improved versions of GEVIs capable of measuring subthreshold Vm. I do have a few points for possible improvements, though:

1. In Fig. 1E, it remained unclear to me what the AUC metric refers to. Please make more explicit what exactly is shown here.

2. Figure 2. In the text, numbers are given for a comparison with ASAP5 using ULoVE imaging. It would be very useful to show comparative example traces in a Suppl. Figure, together with this quantification.

3. Regarding population imaging (Fig. 3) I am wondering about the cell segmentation problem. Even with soma-targeted expression, cell membranes might be difficult to distinguish when cells touch each other (as some do in Fig. S3). For L2/3 it might be OK but for more tightly packed cells it may become a serious issue. Can the authors please mention that issue and comment on it in the text?

4. In Fig. 4, I am puzzled by the example traces in panel C. I understand that the frame rate of imaging was too low to resolve PV spiking (which is probable) during SWR periods, however would one not expect at least increased variance in these traces (similar to the LFP measurement)? Especially since recording frequency and SWR frequency seem similar here, which will lead to aliasing but should show increased variance. Do the authors think that the specific recorded PV cells were spiking or possibly not? Did they see other examples with clear spiking? Please comment and address this issue in the text.

5. Figure 5 is interesting; however, presenting more raw data would be useful here to give the reader a better impression (and intuition) of the JEDI3 performance. Only one trace is shown in B, for which it is not even clear whether it is from soma or dendrite (or VIP?). Examples of dendritic and somatic JEDI3 traces, as well as of JEDI3 traces for VIP interneurons, should be shown in this figure. For the L5 neuronal pool, please also provide information about soma depths and the laser power levels used to image deep somas.

6. The Discussion can be improved. Specifically, I was missing a more thorough comparison with previous or alternative

attempts to optically record subthreshold Vm, including single-photon imaging approaches.

Minor points:
- l.372: ‘expressing’
- l. 434, step sizes rather -80, -75, -70, …

Version 1:

Decision Letter:

Our ref: NMETH-A59507A

6th Oct 2025

Dear Francois,

Thank you for submitting your revised manuscript "Designer indicators for two-photon recording of subthreshold voltage dynamics" (NMETH-A59507A). As I mentioned before, your manuscript has been seen by the original referees and their comments are below. The reviewers find that the paper has improved in revision, and therefore we'll be happy in principle to publish it in Nature Methods, pending minor revisions to satisfy the referees' final requests and to comply with our editorial and formatting guidelines.

We are now performing detailed checks on your paper and will send you a checklist detailing our editorial and formatting requirements within two weeks or so. Please do not upload the final materials and make any revisions until you receive this additional information from us.

TRANSPARENT PEER REVIEW
Nature Methods offers a transparent peer review option for new original research manuscripts. We encourage increased transparency in peer review by publishing the reviewer comments, author rebuttal letters and editorial decision letters if the authors agree. Such peer review material is made available as a supplementary peer review file. **Please state in the cover letter ‘I wish to participate in transparent peer review’ if you want to opt in, or ‘I do not wish to participate in transparent peer review’ if you don’t.** Failure to state your preference will result in delays in accepting your manuscript for publication.

Please note: we allow redactions to authors’ rebuttal and reviewer comments in the interest of confidentiality. If you are concerned about the release of confidential data, please let us know specifically what information you would like to have removed. Please note that we cannot incorporate redactions for any other reasons. Reviewer names will be published in the peer review files if the reviewer signed the comments to authors, or if reviewers explicitly agree to release their name. For more information, please refer to our FAQ page.

ORCID
IMPORTANT: Non-corresponding authors do not have to link their ORCIDs but are encouraged to do so. Please note that it will not be possible to add/modify ORCIDs at proof. Thus, please let your co-authors know that if they wish to have their ORCID added to the paper they must follow the procedure described in the following link prior to acceptance:
https://www.springernature.com/gp/researchers/orcid/orcid-for-nature-research

Author names using non-Roman characters
Nature Portfolio journals can support presentation of author names using non-Roman characters in the HTML version of the article. If you wish to, please include author names in parentheses after the Roman-character spelling; see example online here. Currently supported scripts are: Arabic, Chinese, Cyrillic, Devanagari, Greek, Hebrew, Hangul, Japanese and Persian. You will be asked to verify the rendering is correct at proof stage.

Thank you again for your interest in Nature Methods. Please do not hesitate to contact me if you have any questions. We will be in touch again soon.

Best regards,
Nina

Nina Vogt, PhD
Senior Editor
Nature Methods

Reviewer #2 (Remarks to the Author):

Overview

With the authors' cooperation (21 reviewer critiques and more than 21 author responses) the quality of the manuscript has improved substantially. My assessment of the revised version is largely positive; however, the final four points (A07, A08, A09, and A10) warrant the Editor's immediate attention.

Points

A1
I decided to believe the author's claims that in vitro and in vivo data demonstrate that, under 2P excitation, JEDI3sub and JEDI3hyp outperform ASAP5 in their responses to subthreshold voltage changes and in vitro brightness, while exhibiting similar spike responses.
Comparisons of the 2 indicators conducted in supplemental figures (Fig. S4) are useful (Fig. S8).

A2
I accept the author's claim that JEDI3sub/hyp and JEDI-2P were optimized for 2P, while ASAP3/5 were optimized for 1P.
The authors have updated the text to clarify the advancements brought by JEDI3 indicators over ASAP5 and other state-of-the-art indicators.

A3
I accept the author's claim that Hao et al. (2024) was not included in the reference list by a glitch in the reference manager.

A4
The authors have incorporated the suggested citations (voltage imaging of subthreshold signals) into the Introduction. These citations are most directly related to the current manuscript.

A5
I decided to believe the authors when they state that the observed signals in dendrites reflect genuine voltage fluctuations rather than artifacts such as motion or noise.

A6
It is true that the reviewer wanted to examine the single-trial, single-dendrite fluorescence traces that were used to generate plots such as Fig. 5F. To address this, the authors have now included recordings that show correlated subthreshold signals from two branches of a bifurcating apical dendrite segment (Fig. S14A). Critically, a nearby autofluorescent bleb does not exhibit these fluctuations, reinforcing the interpretation that the JEDI3hyp signals reflect dendritic voltage changes rather than motion artifacts or noise.

A7
The absence of simultaneous whole-cell patch-clamp recordings, widely regarded as the gold standard for validating optical voltage signals, remains a notable limitation, particularly given the authors' demonstrated expertise with in vivo whole-cell recordings.

The reviewing editor should consider the following 4 facts:
1. The reviewers regard simultaneous whole-cell patch-clamp as the gold standard for validating optical voltage signals.
2. In their rebuttal, the authors likewise state that it is the gold standard.
3. However, the current, high-profile study did not include simultaneous whole-cell patch-clamp validation. None.
4. Members of the author team have established in vivo whole-cell expertise (check the list of authors), but did not apply it here.

These 4 points materially affect the strength of the study's conclusions and warrant clarification or additional validation.

A8
The manuscript does not report the robust glutamate-mediated dendritic plateau potentials described by Kerlin et al. (2019). This omission may reflect limitations in the ability of the JEDI3 variants to accurately track dendritic voltage dynamics.

If JEDI3 possessed adequate sensitivity, these sustained dendritic depolarization events would likely produce prominent voltage changes readily detectable in optical recordings.

Unfortunately, the absence of such signals in their data likely reflects problem with indicator sensitivity rather than the rarity of such biological events in experimental conditions.

A9
In their rebuttal the authors attempted to justify the poor sensitivity of their optical recordings (JEDI3) by a statement suggesting that dendritic plateau potentials DO NOT occur in the visual cortex.
Specifically the authors stated:

“In contrast, our recordings were conducted in the visual cortex during spontaneous activity, under conditions where such behaviorally driven, localized dendritic plateaus are not known to occur”.

In reality, dendritic plateaus have been documented in the visual cortex.
1. Valerio Francioni, Zahid Padamsey, Nathalie L Rochefort (2019).
2. Spencer L Smith, Ikuko T Smith, Tiago Branco, Michael Häusser (2013).

Therefore, Nature Methods must NOT accept the omissions made by Land et al. in respect to dendritic plateau potentials.

Land et al. should address the absence of plateau potentials in proper experiments, or at least in the text of the article.

A10
In their rebuttal letter, the authors stated that they accepted the reviewer’s argument for proper terminology “Population Voltage Imaging” vs. “Multi-Cell Imaging”.
However, in line 54, the authors continue to confuse two terms: “Population Imaging” vs. “Multi-Cell Imaging”.
Furthermore, in line 63, the authors continue to confuse two terms: “Population Imaging” vs. “Multi-Cell Imaging”.
Furthermore, in line 931, the authors continue to confuse two terms: “Population Imaging” vs. “Multi-Cell Imaging”.
Land et al. should correct these errors in terminology.

Reviewer #3 (Remarks to the Author):

The authors present a thorough and comprehensive revision of their manuscript, including additional data, further analyses, and clarifications in the text. They have addressed all my specific points adequately. I have no further comments and congratulate the authors on an excellent paper.

Version 2:

Decision Letter:

24th Feb 2026

Dear Francois,

I am pleased to inform you that your Article, "Designer indicators for two-photon recording of subthreshold voltage dynamics", has now been accepted for publication in Nature Methods. The received and accepted dates will be January 21st, 2025 and February 24th, 2026. This note is intended to let you know what to expect from us over the next month or so, and to let you know where to address any further questions.

Over the next few weeks, your paper will be copyedited to ensure that it conforms to Nature Methods style. Once your paper is typeset, you will receive an email with a link to choose the appropriate publishing options for your paper and our Author Services team will be in touch regarding any additional information that may be required. It is extremely important that you let us know now whether you will be difficult to contact over the next month. If this is the case, we ask that you send us the contact information (email, phone and fax) of someone who will be able to check the proofs and deal with any last-minute problems.

After the grant of rights is completed, you will receive a link to your electronic proof via email with a request to make any corrections within 48 hours. If, when you receive your proof, you cannot meet this deadline, please inform us at rjsproduction@springernature.com immediately.

Authors may need to take specific actions to achieve compliance with funder and institutional open access mandates.
If your research is supported by a funder that requires immediate open access (e.g. according to Plan S principles or the NIH public access policy) then you should select the gold OA route, and we will direct you to the compliant route where possible. Because authors warrant under our subscription licensing terms that they haven’t committed to licensing any version of their article under a licence inconsistent with the terms of our agreement – including the applicable embargo period – publication under the subscription model isn’t suitable for authors whose funders require no embargo.

If you have any questions about our publishing options, costs, Open Access requirements, or our legal forms, please contact ASJournals@springernature.com

If you have posted a preprint on any preprint server, please ensure that the preprint details are updated with a publication reference, including the DOI and a URL to the published version of the article on the journal website.

You may wish to make your media relations office aware of your accepted publication, in case they consider it appropriate to organize some internal or external publicity. Once your paper has been scheduled you will receive an email confirming the

publication details. This is normally 3-4 working days in advance of publication. If you need additional notice of the date and time of publication, please let the production team know when you receive the proof of your article to ensure there is sufficient time to coordinate. Further information on our embargo policies can be found here:
https://www.nature.com/authors/policies/embargo.html

To assist our authors in disseminating their research to the broader community, our SharedIt initiative provides you with a unique shareable link that will allow anyone (with or without a subscription) to read the published article. Recipients of the link with a subscription will also be able to download and print the PDF.

As soon as your article is published, you will receive an automated email with your shareable link.

You can now use a single sign-on for all your accounts, view the status of all your manuscript submissions and reviews, access usage statistics for your published articles and download a record of your refereeing activity for the Nature journals.

Please note that you and any of your coauthors will be able to order reprints and single copies of the issue containing your article through Nature Portfolio's reprint website, which is located at http://www.nature.com/reprints/author-reprints.html. If there are any questions about reprints please send an email to author-reprints@nature.com and someone will assist you.

Please feel free to contact me if you have questions about any of these points.

Best regards,
Nina

Nina Vogt, PhD
Senior Editor
Nature Methods

** Visit the Springer Nature Editorial and Publishing website at www.springernature.com/editorial-and-publishing-jobs for more information about our career opportunities. If you have any questions please click here.**

Open Access This Peer Review File is licensed under a Creative Commons Attribution 4.0 International License, which permits use, sharing, adaptation, distribution and reproduction in any medium or format, as long as you give appropriate credit to the original author(s) and the source, provide a link to the Creative Commons license, and indicate if changes were made.
In cases where reviewers are anonymous, credit should be given to 'Anonymous Referee' and the source.
The images or other third party material in this Peer Review File are included in the article's Creative Commons license, unless indicated otherwise in a credit line to the material. If material is not included in the article's Creative Commons license and your intended use is not permitted by statutory regulation or exceeds the permitted use, you will need to obtain permission directly from the copyright holder.
To view a copy of this license, visit https://creativecommons.org/licenses/by/4.0/

RESPONSE TO REVIEWS

Title: "Designer indicators for two-photon recording of subthreshold voltage dynamics" (NMETH-A59507-T)

INTRODUCTION TO THE RESPONSE

We sincerely thank the reviewers for their thorough evaluation of our manuscript and for providing thoughtful, constructive feedback. We have worked diligently to fully address each of the concerns raised, including new text and many new supplementary figures, as detailed in this 17-page response. We hope that the depth, completeness, and rigor of our revisions will support a recommendation for publication!

Note that substantial changes to the manuscript and captions are indicated with blue (rather than black) text.

REVIEWER'S COMMENTS

REVIEWER #2

Land et al. focus on advancing genetically encoded voltage indicators (GEVIs). In their manuscript, they argue that existing GEVIs lack sufficient sensitivity for detecting millivolt-scale subthreshold signals. To address this, they have refined a multiparametric two-photon high-throughput screening platform to develop two novel indicators—JEDI3sub and JEDI3hyp—designed for subthreshold voltage detection in both neuronal cell bodies and dendrites. Using fast two-photon (2P) optical recordings in awake, behaving mice, the authors report that the new JEDI3 variants outperform the earlier JEDI-2P.

POINT 1: Comparison with Prior Studies.

A recent Neuron paper (2024) by Dr. M. Z. Lin—St-Pierre's former postdoctoral advisor—introduced ASAP5, a novel GEVI capable of fast 2P optical recording in live mice. Notably, ASAP5 was demonstrated to record subthreshold membrane potential transients in dendrites of cultured cells. There is a significant overlap between the current study and the findings reported by Hao et al. (2024), as outlined below:

[A] Two-Photon Microscopy: Land et al. claim that their new GEVI variant is optimized for deep-tissue recording using 2P microscopy. However, Hao et al. (2024) have already established that ASAP5 performs well under similar imaging conditions.

We thank the reviewer for the opportunity to further clarify the distinctions between the ASAP5 and JEDI3sub and JEDI3hyp sensors beyond the performance comparisons presented in the original manuscript. In response, we conducted extensive in vitro and in vivo comparisons to address the concerns raised. We demonstrate that ASAP5 has comparable performance to JEDI-2P, and that JEDI3 indicators outperform ASAP5.

<u>In Vitro Comparisons</u>

JEDI3sub exhibits approximately twice the response amplitude of ASAP5 to subthreshold fluctuations and 100-mV voltage steps under laser-scanning two-photon (2P) excitation (Fig. S4). The voltage response curve of ASAP5 under 2P is comparable to that of JEDI-2P, the parental indicator of JEDI3 indicators. Notably, ASAP5 shows significantly larger responses under widefield one-photon illumination (Hao et al., 2024) than 2P excitation (this study), consistent with prior findings that ASAP3, the parental indicator of ASAP5, performs better under 1P than 2P (Liu et al., 2022).

We also compared the 2P brightness of ASAP5, JEDI-2P, JEDI3sub, and JEDI3hyp at 940 nm. A 940-nm-excitable red-emitting fluorescent protein (mBeRFP) was appended to the C-termini of each GEVI. To control for biological and experimental variation, we normalized the brightness of each GEVI to the intensity value of this mBeRFP control. All three JEDI indicators were brighter than ASAP5 (Fig. S4B). For example, JEDI3sub was 1.4-fold brighter than ASAP5. This brightness differential mirrors that observed between their parental indicators, JEDI-2P and ASAP3 (Liu et al., 2022). These findings align with Hao et al.'s data in flies, where JEDI-2P was ~20% brighter than ASAP5, and with their quantification showing ASAP5 and ASAP3 have similar brightness.

We note that Hao et al. also reported data where JEDI-2P-Kv appeared dimmer than ASAP5-Kv in layer 2 motor cortex mice neurons in vivo. However, this result may reflect variability in AAV transduction efficiency —factors influenced by differences in AAV lot, injection parameters, animal-to-animal variability, or the spatial relationship between the injection

site and the imaged cells— in cranial window quality. Co-expressing a non-voltage-responsive reference protein via a 2A sequence or covalent link to the GEVI, as done above for our in vitro experiments, would help correct for these factors. However, we (and others) have found that such co-expression, while well tolerated in vitro, impairs GEVI plasma membrane localization in mouse models. GEVIs are thus typically expressed without an expression control in mice, as was the case in Hao et al. and in this study. In this context, brightness comparisons in vivo are prone to confounding variables, motivating our decision to exclusively compare GEVI brightness in vitro.

In Vivo Comparisons

Since the same authors acquired ULoVE data for the ASAP5 paper and this manuscript, we could compare indicator performance data under identical optical acquisition conditions. JEDI3sub-Kv and JEDIhyp-Kv exhibited approximately twice the response to subthreshold fluctuations compared with ASAP5-Kv (Fig. S8), consistent with their respective in vitro voltage response curves (Fig. S4). ASAP5-Kv responses to subthreshold fluctuations were similar to, but statistically lower than, those of JEDI-2P-Kv. ASAP5-Kv's spike responses were also similar (but statistically smaller) than those of JEDI-2P-Kv, consistent with Hao et al.'s study, which showed statistically indistinguishable spike response amplitudes between these two indicators (e.g., Fig. 4A in Hao et al.). ASAP5-Kv's spike responses were statistically indistinguishable from those of JEDI3sub-Kv, and slightly larger than JEDI3hyp-Kv. JEDI3 indicators also demonstrated a larger dynamic range than ASAP5-Kv, while all indicators showed similar spike widths.

Conclusion

Taken together, our in vitro and in vivo data demonstrate that, under 2P excitation, JEDI3sub and JEDI3hyp outperform ASAP5 in their responses to subthreshold voltage changes and in vitro brightness, while exhibiting similar spike responses. ASAP5 performed similarly to JEDI-2P, exhibiting comparable voltage response curves in vitro (Fig. S4), as well as statistically indistinguishable dynamic ranges, spike responses, and subthreshold fluctuations (Fig. S8).

JEDI3sub/hyp and JEDI-2P were optimized for 2P, while ASAP3/5 were optimized for 1P. The observation that ASAP5 shows dramatically reduced responses under 2P compared with 1P —similar to what we reported for ASAP3[1]— supports our contention that GEVIs should be optimized for the intended imaging modality.

[B] Subthreshold Voltage Detection: While Land et al. emphasize that their GEVI is tailored specifically for subthreshold voltage detection, Hao et al. (2024) demonstrated that ASAP5 effectively detected miniature excitatory postsynaptic potentials ("minis").

We appreciate the reviewer allowing us to clarify the value of JEDI3 indicators given the recent availability of ASAP5. Hao et al. demonstrated that ASAP5 can detect miniature excitatory postsynaptic potentials ("minis") in **cultured (*in vitro*)** hippocampal neurons and human iNs under **one-photon (1P) illumination** (Hao et al., Figures 5–7). However, our study focuses on ***in vivo*** applications using **2P excitation**, arguably the most common method for non-invasive brain imaging.

As discussed above, we conducted new experiments to address the relative performance of these indicators under 2P in vitro (Fig. S4) and in vivo (Fig. S8). These data, included in the revised manuscript, show that JEDI3 indicators produce approximately **twice the response** to subthreshold voltage changes under 2P excitation.

As demonstrated in the original manuscript, we leveraged these improvements to monitor brain state–dependent voltage dynamics using JEDI3hyp in layer 5 pyramidal neurons and their dendrites in vivo (Figure 5E–F). Furthermore, we were able to detect 2–3 mV pupil-associated subthreshold voltage changes in VIP- and SOM-positive interneurons using JEDI3hyp in vivo (Figure 5G and Figure S5D, F, and Rebuttal Figure 1.1, *below*). These experiments, along with others presented in the manuscript, provide direct evidence that JEDI3 indicators are well-suited for in vivo subthreshold voltage imaging under various experimental conditions and imaging modalities.

We have updated the text to clarify the advancements brought by JEDI3 indicators over ASAP5 and other state-of-the-art indicators.

[redacted]

[C] Dendritic Recordings: Land et al. highlight breakthrough recordings of voltage changes in fine dendritic structures. Yet, recordings of voltage fluctuations in fine dendrites were also presented in Hao et al. (2024).
Given these overlaps, it is concerning that Hao et al. (2024) is not included in the reference list. Its relegation to the Methods section appears to minimize its prominence, and does not adequately acknowledge its contribution.

Hao et al. was discussed and cited in the original submission, including in the paragraph beginning with the following sentence:

> *"A recently reported indicator, ASAP5, demonstrated the ability to detect single-trial mEPSPs in cultured neurons under one-photon illumination (Hao et al., 2024). (...)"*

However, due to an issue with our citation manager, the reference became corrupted; as a result, it didn't appear as a subscript and did not show up in the final list of citations. We have corrected this oversight in the revised manuscript.

It is recommended that Hao et al. (2024) be cited and discussed in the Introduction to clarify how the current work advances the field. A similar approach should be taken with respect to Xie et al. (2021), by explaining in both the Introduction and Discussion how Land et al.'s findings build upon and differ from these earlier studies.
In addition to Hao et al. (2024) and Xie et al. (2021), several other key publications have contributed to the field of dendritic voltage imaging but were not referenced by Land et al. These include:
- Adam E. Cohen's work on Voltron recordings in dendrites (presented at conferences and available on BioRxiv), which explores dendritic excitations.
- Dorgans et al. (2020), who imaged subthreshold voltage oscillations with cellular resolution in the inferior olive in vitro (Front. Cell. Neurosci.).
- Jin et al. (2012), which reported imaging of subthreshold events in neurons using a fluorescent protein voltage probe (Neuron).
- Berger et al. (2007), combining voltage and calcium epifluorescence imaging to reveal both subthreshold and suprathreshold dynamics in mouse barrel cortex (J. Neurophysiol.).
- Djurisic et al. (2004), which detailed voltage imaging of EPSPs from dendrites of mitral cells (J. Neurosci.).

We thank the Reviewer for the excellent suggestion to include additional references to better contextualize the advancements presented in our manuscript. In response, we have incorporated all the suggested citations into the Introduction and have also referenced several of them in the Discussion. We added a few additional studies, although we're already exceeding the number of citations allowed by Nature Methods. The study by Hao et al. (2024) is further discussed in a few Results subsections, given the addition of new comparisons between JEDI3 indicators and ASAP5, as mentioned earlier in this document.
Due to the word limit imposed by the journal —which we have already severely exceeded in addressing other reviewer comments— we were unable to provide detailed descriptions of each cited study. A comprehensive treatment of these

critical contributions would be more appropriate for a dedicated review article. Instead, we focused on summarizing the general classes of experiments enabled by these pioneering works and the broader limitations that motivated our study, including the need for GEVIs over alternative tools, and the relative advantages of 2P over 1P voltage imaging for key neuroscience applications. While our manuscript includes voltage imaging in dendrites, we wish to emphasize that its primary goal is to introduce a biosensor optimized for 2P voltage imaging, rather than to report new biological discoveries (in dendritic computations or otherwise), as is the focus of several of the cited publications.

POINT 2: Interpretation of Optical Traces (Lines 202 & Figures 2B and 3).

The manuscript asserts that "these results demonstrate JEDI3sub's capacity to track subthreshold activity across large neuronal populations, enabling investigation into population-level information processing." However, the data presented in Fig. 3 appear to be derived from noisy optical traces whose undulations may be artifactual. Even the best recordings (Fig. 2B) do not unequivocally differentiate subthreshold depolarizations from scanning or mechanical artifacts (cardiovascular, breathing, etc.). Without concurrent whole-cell recordings—as exemplified by Reimer et al. (2014)—it is challenging to confirm that the observed signals represent genuine subthreshold events. Note that Reimer is on the author list of the current manuscript.

We thank the Reviewer for the opportunity to clarify the basis for our interpretation that the observed subthreshold signals reflect genuine voltage dynamics rather than optical recording artifacts. We agree that differentiating true subthreshold activity from potential artifacts —including those arising from scanning limitations, mechanical sources (e.g., respiration, heartbeat), or shifts in the imaging plane— is essential for the interpretation of optical voltage data.

Scanning artifacts

The Reviewer brought up scanning artifacts as a possible source of variation. The data presented in Fig. 2 was acquired by ULoVE microscopy where multiplexed excitation points rapidly scan an excitation volume (marked with white trapezoids in Fig. 2A). The data shows large subthreshold changes despite being acquired at a high sampling rate of 7 kHz (143 µs per time point), which oversamples relative to the timescales of typical subthreshold events such as EPSPs, IPSPs, and cortical up/down states —supporting our claim that scanning artifacts are not responsible for these fluctuations.

To fully address the concern that the observed subthreshold fluctuations could be ULoVE-specific scanning artifacts, we conducted measurements using an alternative technique, in which we performed rapid (~2 kHz) raster AOD-based scanning of neuronal activity. These experiments show neural activity that is quantitatively (Fig. S6G) and qualitatively (Fig. S6B, zoom-in) similar to that acquired with ULoVE. These results demonstrate that the sub-threshold fluctuations recorded with ULoVE (e.g., Fig. 2B) are not artifacts due to the specific scanning method used.

We agree that aliasing can occur in resonant scanning, particularly when imaging a FOV larger than individual cells (particularly along the y axis), where signals can be lost or distorted if the laser is not sampling a cell's pixels frequently enough. The FACED and resonant scanning data (e.g., Figs. 3–4) was acquired at frame rates ranging from 288 Hz to 769 Hz, fast enough to capture the relatively slow subthreshold dynamics reported here. However, aliasing of spike responses may contribute to the depolarization signals observed. In particular, depolarizations captured from PV cells (Fig. 4) likely reflect a mixture of subthreshold and suprathreshold activity, since the chosen temporal resolution (288 Hz, ~3.5 ms/frame) is slower than the timescale of optical responses to PV spikes (likely < 1 ms width). As a result, spike responses can be aliased, contributing to the depolarization responses. To highlight these considerations, we edited the text as follows:

- *PV cell section*. We added a sentence to clarify that spike aliasing can contribute to signals.
- *Discussion*. We discussed aliasing in a new Discussion section titled "Challenges: mechanical artifacts, temporal aliasing, and signal contamination."

Mechanical artifacts.

With respect to mechanical artifacts such as those caused by breathing or animal motion, we took several experimental and analytical steps, in the original manuscript, to distinguish these from genuine voltage signals:

- The ULoVE excitation volume shape was specifically designed to be robust against motion artifacts, as previously demonstrated (Villette et al., 2019, https://doi.org/10.1016/j.cell.2019.11.004).
- Our new indicators, JEDI3sub and JEDI3hyp, exhibit larger fluorescence fluctuations than JEDI-2P (Fig. 2), as expected based on their greater response amplitudes to subthreshold fluctuations (Fig. 1H). There is no parsimonious mechanism by which this difference would arise from motion artifacts, which would be expected to affect all indicators similarly.

- The spike-triggered averages (Fig. 2C) exhibit the expected waveform shape, which would not be preserved if fluctuations were predominantly due to mechanical artifacts.
- Action potentials occur atop the largest fluctuations (Fig. 2B), consistent with spikes riding on top of UP states. If the signal were dominated by motion artifacts, spikes would instead be randomly distributed, including above apparent troughs.
- Some features of our voltage imaging data align with prior electrophysiological recordings. For instance, our optical recordings in Fig. 5 are consistent with observations from Reimer et al. (2014) —including voltage fluctuations associated with pupil dilation— which further support the interpretation that these are genuine subthreshold fluctuations rather than artifacts.

We conducted several new experiments to fully address the Reviewer's concern:

1. Using fast imaging of JEDI3sub-Kv activity with Regions-of-Interests (ROIs) with similar size as the ULoVE patterns we used, we extracted the displacements of the cell over time in 3D by auto-correlation with a reference image stack (Fig. S6A). The correlation between the displacements and the activity recorded was extremely low (< $5*10^{-2}$) for movements ≤ 2.5 µm (Fig. S7D). Given that 99% of the displacement amplitudes observed in our experiments are ≤ 2.5 µm (Fig. S6C), this result indicates that the ULoVE patterns we used were appropriately sized to compensate for brain motions during our in vivo recordings.

2. We provide two Supplementary Videos (Supplementary Movies 1-2) of JEDI3sub-Kv fluorescence dynamics to illustrate that large fluctuations in brightness are not attributable to motion artifacts.

3. To validate the above findings with fast imaging, we developed a method to extract cell displacement data during ULoVE recordings (Fig. S6E). Our analysis revealed no significant correlation between these displacements and the ULoVE functional signals. This result supports the conclusion that the observed signals reflect genuine GEVI voltage modulations rather than motion artifacts.

4. To investigate potential physiological artifacts, we analyzed fast imaging data from autofluorescent objects located near blood vessels. We found that signal components at frequencies characteristic of respiration and heartbeat were indeed present in these control recordings. However, such frequency components were absent from both the ULoVE recordings and the fast imaging data acquired from JEDI3sub (Fig. S7A–G). These findings support the conclusion that the ULoVE signals reflect genuine voltage dynamics rather than motion or physiological artifacts.

5. We also provide a timelapse video from the dataset shown in Fig. 3 (Supplementary Movie 3). This motion-corrected movie shows clear fluorescence fluctuations, suggesting that they represent genuine voltage shifts rather than motion artifacts. The lateral shifts depicted in Fig. S9B,C, which we quantified, were small and easily accounted for during image registration. Additionally, trial-locked, stimulus-specific subthreshold dynamics and structured patterns of synchronized population activity (Fig. 3D) further support the physiological origin of the signals.

Besides these new supplementary figures and videos, and the corresponding changes to the text, we made the following revisions to the manuscript and expanded the Discussion with a new section in the Discussion titled "*Challenges: mechanical artifacts, temporal aliasing, and signal contamination*", which explicitly addresses issues raised by the Reviewer.

Conclusion.

We believe we have thoroughly addressed the Reviewer's concerns, and to the best of our knowledge, our evaluation of motion and scanning artifacts is the most detailed to date in the GEVI optical recording literature. While we fully agree that simultaneous whole-cell patch-clamp recordings represent the gold standard for validating optical voltage signals, such experiments are technically challenging to perform in vivo —particularly in awake, behaving animals, as used in our study. Nevertheless, our multiple complementary approaches rigorously and quantitatively evaluated the fidelity of the optical signals reported.

POINT 3: Replication of Electrophysiological Findings (Line 252 and Fig. 5).

The statement that "Using 2P voltage imaging with resonant-scanning microscopy, we confirmed that JEDI3hyp-Kv replicated these electrophysiological findings..." is not convincingly supported. In Fig. 5B, the optical traces are difficult to interpret without additional physiological data, making it unclear whether the observed undulations reflect meaningful electrophysiological events.

We thank the Reviewer for this opportunity to clarify our findings. We observed that pupil dilation robustly suppresses 2–10 Hz voltage oscillations in layer 2/3 pyramidal neurons, while pupil constriction enhances them (Fig. S5A,B, see below). These changes are clearly distinguishable from noise, as detailed below, and support our interpretation of the optical signals.

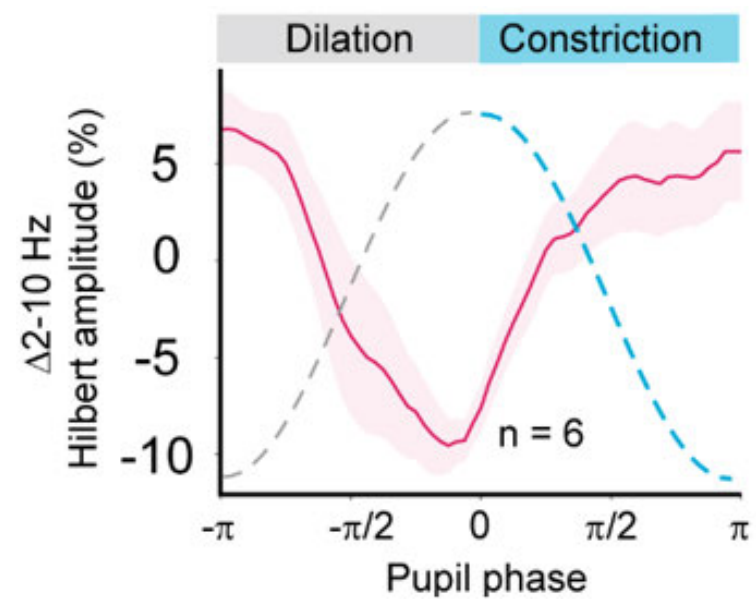

To improve clarity, we have revised the original sentence from: "we confirmed that JEDI3hyp-Kv replicated these electrophysiological findings" to "we observed that JEDI3hyp-Kv could optically report this activity pattern."

We appreciate the Reviewer's concern that the observed undulations might not reflect meaningful electrophysiological events. To address this directly, we performed a control experiment measuring fluorescence from nearby autofluorescent structures ("blebs"), which are not expected to exhibit voltage-dependent signals. These control regions showed no correlation between fluorescence oscillations in the 2–10 Hz range and pupil diameter (Fig. S14). In contrast, adjacent apical dendrites exhibited strong oscillations at 2–10 Hz that were tightly correlated with pupil dynamics.

This differential response strongly supports the conclusion that the observed signals in dendrites reflect genuine voltage fluctuations rather than artifacts such as motion or noise. We have edited the main text to describe the findings from these new control experiments.

POINT 4: Dendritic Recordings (Figure 5E):

The authors claim to record dendritic potentials in the terminal branches of cortical pyramidal cells. However, a closer inspection of Fig. 5E reveals an absence of such convincing recordings. This raises concerns about the method's reliability for capturing dendritic subthreshold depolarizations.

First, we would like to clarify that our recordings were obtained from distal, but not terminal, dendritic segments of layer 2/3 pyramidal neurons.

Figure 5E presents a representative example of an apical dendrite from which JEDI3hyp signals were recorded, while Figure 5F (top) summarizes the processed data across all recorded dendritic fragments (n = 44). These data reveal consistent changes in slow subthreshold oscillations that exhibit a specific phase relationship with pupil diameter, supporting the conclusion that our method captures biologically meaningful subthreshold voltage dynamics.

We acknowledge that the reviewer may have wanted to examine the single-trial, single-dendrite fluorescence traces that we used to generate plots such as Fig. 5F. To address this, we have now included exemplar recordings that show correlated subthreshold signals from two branches of a bifurcating apical dendrite segment (Fig. S14A). Critically, a nearby autofluorescent bleb does not exhibit these fluctuations, reinforcing the interpretation that the JEDI3hyp signals reflect genuine dendritic voltage changes rather than motion artifacts or noise. We have also provided example recordings from VIP+ interneurons (Fig. S15B) and additional examples from Layer-5 IT pyramidal cells (Fig. S12C).

POINT 5: Missing Dendritic Plateau Potentials.

The manuscript does not report the robust glutamate-mediated dendritic plateau potentials described by Kerlin et al. (2019). This omission may reflect limitations in the ability of the JEDI3 variants to accurately track dendritic voltage dynamics.

The study by Kerlin et al. (2019, [DOI: 10.7554/eLife.46966]) reports sustained, branch-specific calcium signals — interpreted as glutamate-mediated dendritic plateau potentials— primarily in the apical dendrites of layer 2/3 and layer 5 neurons in the motor cortex during a tactile discrimination task. In contrast, our recordings were conducted in the visual cortex during spontaneous activity, under conditions where such behaviorally driven, localized dendritic plateaus are not known to occur. Given these substantial differences in brain region, task engagement, and cellular context, there is no compelling reason to assume that similar plateau potentials should be observed in our experimental paradigm.

These sustained events would likely produce prominent voltage changes readily detectable by our high-sensitivity indicators, JEDI3sub and JEDI3hyp. Indeed, the absence of such signals in our data likely reflects their rarity in our experimental conditions, rather than an indicator sensitivity limitation. On the contrary, our findings demonstrate that JEDI3sub/hyp reliably detects subtle subthreshold dynamics, such as low-amplitude voltage oscillations with specific relationships to pupil diameter (Fig. 5, Rebuttal Fig. 1.1), underscoring their ability to resolve subtle physiologically meaningful voltage signals.

While a systematic investigation of GEVI performance in detecting dendritic plateau potentials under behaviorally driven paradigms would be informative, such an analysis is beyond the scope of the current study. Nonetheless, our results support the utility of JEDI3sub/hyp for capturing subthreshold voltage dynamics with high fidelity.

POINT 6: Terminology: "Population Voltage Imaging" vs. "Multi-Cell Imaging".

The term "population voltage imaging" was erroneously used by biochemists and protein designers. The fact that biochemists and molecular cell biologists focused on making new GEVIs (Ed Boyden, Adam Cohen, and others) had erroneously dubbed their multi-cell recordings as "population imaging" (e.g., Piatkevich et al. 2019), does not warrant a mindless propagation of this erroneous terminology. The current authors erroneously refer to their approach as "population voltage imaging" (line 670). Traditionally, this term is reserved for techniques that record an aggregate response from many neurons (e.g., wide-field imaging). In population imaging experiments, activity of many elements is mixed into one representative signal; an aggregate signal that represents a given population. Furthermore, the term "population voltage imaging" is a well-established terminology in neuroscience, by pioneers of voltage imaging including Brian M. Salzberg, Amiram Grinvald, Carl Petersen, and others. For further readings, do check: Momose-Sato and Sato 2019; Storace et al. 2019; Short and Wachowiak 2019; Baker et al. 2015; Allen et al. 2017; Pinto et al. 2019. In contrast, methods that record individual neurons are better described as "multi-cell imaging". The authors should revise their terminology accordingly to prevent confusion ensuring that the terms accurately reflect the spatial resolution and methodological approach of their study.

We thank the reviewer for clarifying this nomenclature standard. We have adjusted our terminology accordingly.

POINT 7: Membrane Localization (Line 107)

The manuscript states, "Confocal microscopy revealed robust plasma membrane localization in the soma and dendrites of dissociated rat hippocampal neurons for both JEDI3 variants (Fig. 1B)." Relying on a single example (Fig. 1B) is insufficient to substantiate a claim of "robust" membrane localization. In scientific reporting, strong claims require comprehensive and reproducible experimental evidence—ideally supported by rigorous statistical analysis that demonstrates resistance to noise and outliers. The authors should expand their dataset and clearly describe the statistical methods used to validate membrane localization.

We thank the reviewer for this comment and fully agree that the use of the term "robust" to describe membrane localization was not adequately supported. In response to the reviewer's suggestion, we have revised multiple sections of the manuscript to strengthen the accuracy and rigor of our reporting of GEVI membrane localization. Specifically:

1. In vitro confocal imaging:

- We have removed the term "robust" from our description of membrane localization in dissociated rat hippocampal neurons. The revised text now states that confocal microscopy qualitatively showed that these mutations did not prevent expression of JEDI3 indicators at the plasma membrane of dissociated rat hippocampal neurons.
- We have included additional representative confocal images of dissociated neurons expressing JEDI3hyp and JEDI3sub (Figure S2, 2 additional images per indicator).

2. In vivo membrane localization:

Membrane localization can vary depending on the expression level and trafficking capacity. For instance, overexpression can exceed the capacity of the cellular trafficking machinery, leading to increased intracellular retention of the indicator. These variables further emphasize the importance of validating performance under in vivo conditions, which are more relevant to end users.

- We performed a detailed quantitative analysis of fluorescence distribution using microscopy images acquired before in vivo ULoVE optical recording (Fig. 2). This analysis showed that JEDI3sub-Kv and JEDI3hyp-Kv exhibit membrane localization levels comparable to JEDI-2P-Kv (Fig. S5), supporting their suitability for in vivo imaging. All indicators displayed approximately 75% of their fluorescence associated with the plasma membrane. However, this value likely underestimates true membrane localization due to the limited optical sectioning capability of two-photon microscopy, which uses longer excitation wavelengths than one-photon confocal imaging. As a result, fluorescence from the top and bottom membranes can blur into adjacent cytoplasmic regions, artificially lowering the measured proportion of membrane-localized signal.
- Additionally, we now provide a confocal Z-stack of a representative JEDI3hyp-Kv-expressing PV interneuron in vivo, as an additional, qualitative illustration of membrane localization in a different cell type and context (Fig. S11C).

POINT 8: Specification of Physical Units and Methodological References.

Throughout the Methods, critical parameters are described without specifying physical units or citing established protocols. For example, in line 398 the "detectability index" is defined as the response amplitude (units not provided) multiplied by the square root of the relative brightness (units not provided). A reference detailing the procedure for these measurements is also missing. The authors must:

- Clearly state the physical units for all quantitative parameters.
- Provide a citation or detailed description of the adopted procedures, particularly for parameters like photostability and detectability.

We thank the reviewer for bringing this point to our attention. In response, we have revised the Methods section "Two-photon GEVI screening system" to explicitly clarify how each parameter, such as the detectability index and photostability, was calculated. Rather than relying on citing prior work, we now provide detailed descriptions of the computational procedures used for all relevant metrics.

We want to clarify that the parameters in question are intentionally unitless or expressed in arbitrary units ("Arb. U.") because they represent relative, normalized values used for comparative ranking of indicators during the screening process. Their primary purpose is to enable consistent within-study comparisons rather than to reflect absolute physical quantities. We have now made this explicit by:

- Adding "Arb. U." where appropriate throughout the manuscript, including the Methods.
- Including an explanatory sentence at the end of the relevant Methods subsection to clarify that these parameters are relative and unitless by design.

POINT 9: Calcium Indicators and Subthreshold Activity (Line 55).

The manuscript states that "because GECIs detect calcium influx associated with APs, they generally cannot report subthreshold activity." This statement is inaccurate, as recent evidence indicates that calcium indicators can, in fact, detect subthreshold neuronal activity (Milicevic et al., 2024, Sci. Rep., PMID: 39367010).

We thank the Reviewer for bringing this important and timely study to our attention. We have revised the manuscript to acknowledge that GECIs can, under certain conditions, detect calcium signals associated with subthreshold activity, and we now cite the work by Milicevic et al. (2024). The revised sentence now reads:

> "Although GECIs can detect calcium influx associated with large subthreshold fluctuations in specific preparations (Milicevic et al., 2024), they remain intrinsically ill-suited to directly report subtle changes in membrane potential."

POINT 10: Clarity in Biological Preparations (Fig. 1)

Figure 1 presents data from different biological preparations (cortical neurons and HEK cells) without clear labeling. For example, the caption for Fig. 1D and the pyramidal neuron image in Fig. 1B suggest that all data are neuron-specific, which is misleading. Similarly, in panel G the description—"JEDI3 indicators produced larger steady-state responses…" with data from 7–10 cells per variant—appears alongside a neuron image, potentially overstating its biological relevance.
Ø Clearly label panels that display HEK cell data (e.g., include "HEK cell" as a label).

We thank the reviewer for pointing out the potential for confusion regarding the biological source of the data in Figure 1. To clarify this, we have added the statement "Data acquired in HEK cells except for panel (B)" immediately below the title of Figure 1. We believe this provides readers with a clear, high-level indication of the experimental context for each panel.

In addition, the figure legend already specifies the cell type used for each measurement, as was included in the original submission. While we considered labeling each subpanel with "HEK cell" or "neuron," we feel that doing so would unnecessarily clutter the figure and deviate from standard practices in the field, where such annotations are typically reserved for the legend or caption.

We hope the reviewer finds that these revisions strike an appropriate balance between clarity and visual clarity, and that they adequately resolve the concern.

POINT 11

For Fig. 1I, the caption "Traces are the mean responses" is inadequate because:
1. The meaning of the combined "faint" and "darker" traces is not explained.
2. The number of traces used to generate the mean is not provided.

We thank the reviewer for bringing this point to our attention. In response, we revised the caption to read: "Dark traces are the mean responses."

To clarify the shaded areas, we note that the figure legend includes a general statement at the end: "For all panels, error bars or shaded areas denote the 95% CI." This summary was intentionally placed at the end of the legend to avoid redundant definitions for each subpanel, in line with the space constraints and style guidelines of Nature journals.

The number of traces used to generate the mean is provided in the subpanel description: "n = 7–10 HEK293A cells/variant."

POINT 12

Fig. 2. (C) caption: "Mean optical spike waveforms from the recordings shown in panel (B)". This caption is below a usual level of standard on two levels: First, traces in panel (B) contain AP-singlets, AP-doublets, and AP-triplets. Apparently, the authors cherry-picked some APs which they wished to include into the mean trace, or they did something else, which must be explained.

Second, the sample size (i.e., the number of traces) used to compute the mean is omitted. The authors want to publish this result in Nature Methods without stating explicitly the number of traces used to generate the mean trace. The authors should provide a detailed explanation of how these AP were selected for inclusion into the mean and how averages were computed, and state the corresponding sample sizes (e.g., "n = XX traces").

We appreciate the reviewer's close reading and agree that transparency in sample size and methodology is essential. We want to emphasize that we take our responsibilities as scientists and engineers seriously and are committed to conducting rigorous and transparent research. We agree that additional clarification is warranted and have revised the text accordingly.

Specifically, we now clarify in the caption of Figure 2 and in the Methods section that the mean optical trace includes all isolated spikes (that were separated by more than 50 ms) from the representative neuron. We excluded spikes occurring in bursts (e.g., doublets and triplets), as their inclusion would distort the average trace due to overlapping signals. The exact number of isolated spikes used to compute the average is now explicitly stated in the figure caption.

Additionally, in the new supplementary Figure S8, we provide a direct comparison of spike-triggered averages computed from (a) isolated spikes only and (b) all spikes. This allows the reviewer to assess how the inclusion of burst events affects the average trace.

We also emphasize that our broader quantitative analysis —including dynamic range (Fig. 2D), subthreshold response range (Fig. 2E), spike amplitude (Fig. 2F), spike width (Fig. 2G), and photostability (Figs. 2H–I)— was conducted across multiple neurons and recordings, as reflected in the sample sizes reported in those panels and throughout the text.

POINT 13: Quantification of Subthreshold Responses (Line 155)

The sentence,“Suprathreshold (AP) response amplitudes were derived from spike-triggered averages (Fig. 2C), while subthreshold responses were quantified as the range of the low pass-filtered trace,”

is ambiguous for several reasons:

1. Definition of “Range”: The term “range” is used without a clear definition. The authors should explain how “range” is calculated in this context.

2. Visual Representation: It is unclear where the subthreshold responses are shown in Fig. 2, and no visual markers (e.g., arrows or labels) are used to highlight these features.

We agree that these terms and figures were not optimally communicated. To enhance clarity and visualization, we now explicitly indicate the “subthreshold fluctuations range” in the figure as the span between high-voltage (dark pink) and low-voltage (light pink) subthreshold states, along with the distribution of the filtered trace. The Methods section describes how these high and low states were defined, and we have further revised that section to improve clarity and transparency.

3. Baseline Ambiguity: A closer look at Fig. 2B suggests that the baseline voltage—where subthreshold events begin—is not well defined due to high-frequency noise and low-frequency fluctuations. This uncertainty casts doubt on the reliability of the subthreshold amplitude measurements.
A more robust method for baseline determination and clear labeling of subthreshold events is necessary.

(Note: We assume the reviewer intended to refer to Fig. 2B, not 1B, and have responded accordingly.)

Identification of low-frequency fluctuations and baseline ambiguity:

We thank the reviewer for raising this important point. Indeed, photon shot noise introduces high-frequency variability that imposes a fundamental limit on the fidelity of optical voltage measurements. To address this, we have added a new panel (Fig. S8) that illustrates the extent of shot noise as a gray band overlaid on the optical traces of the four GEVI variants. This visual aid helps distinguish physiological signals from measurement noise and supports the robustness of our subthreshold amplitude estimates.

Clarification of the baseline definition:

We agree that the concept of a fixed “baseline” is not straightforward in our experimental context. The neurons we study in vivo exhibit continuous fluctuations in membrane potential due to intrinsic dynamics and ongoing synaptic input. As such, there is no static or stimulus-locked baseline from which discrete subthreshold events emerge. Instead, the optical signals reflect the full range of subthreshold voltage dynamics, including transitions between high- and low-voltage states—phenomena analogous to UP and DOWN states observed during sleep or anesthesia (e.g., doi:10.1038/nn.3464).

To clarify this in the manuscript, we have revised the main text to emphasize that our analysis focuses on characterizing the distribution of subthreshold membrane potentials rather than detecting discrete, event-like deviations from a fixed baseline.

POINT 14: Discrepancy Between Quantified Data and Representative Traces

The reported quantifications indicate that, for JEDI3sub, the mean subthreshold amplitude (40% dF/F) is approximately twice the mean spike amplitude (20% dF/F) (Figs. 2E and 2F). This would imply that in real traces, APs should appear as small, rapid transients superimposed on large subthreshold “hills.” However, the representative trace in Fig. 2C shows an approximately 1:1 ratio between AP amplitude and subthreshold depolarization. Such a discrepancy between quantification and representative data undermines the reliability of the reported results. The authors must reconcile these differences.

(NB: we replaced 1C with 2C in the comment above, as it was a typo and the reviewer clearly meant 2C)

We appreciate the reviewer sharing this perceptive observation. The primary reason is that "subthreshold amplitude" in Fig.2E (now called: "subthreshold fluctuations range") corresponds to the full range of the subthreshold fluctuation distribution, from the lowest fluorescence during hyperpolarizations to the median spiking threshold. (The exact mathematical definitions are described in the methods).

In contrast, spikes arise from an already depolarized UP state rather than from the trough of hyperpolarizations. As a result, the fluorescence change observed beneath a spike does not reflect the full dynamic range of membrane potentials that GEVI-expressing neurons can experience or report through changes in brightness. To illustrate this point, the figure below presents a trace segment from Fig. 2, highlighting an isolated spike (Rebuttal Fig. 13.1). Red circles approximately indicate the width of segments we used to generate mean optical spike waveforms, such as those shown in Fig. 2C. Notably, the fluorescence changes within this window capture only a narrow portion of the broader subthreshold voltage fluctuations, which we define as the span between the gray and pink dashed lines —including potential hyperpolarizations, as indicated by the arrows.

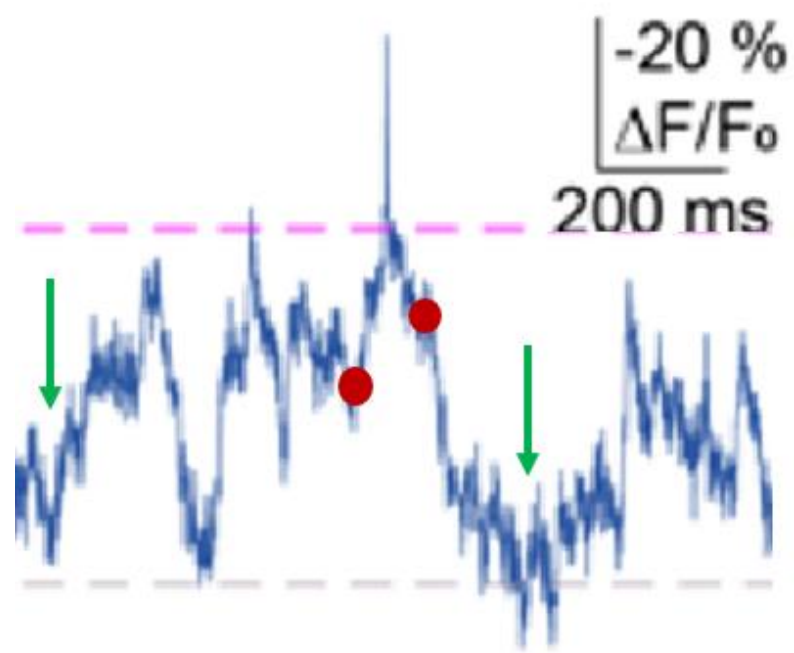

Rebuttal Figure 13.1. Adapted from Fig. 2B

To clarify these concepts, we made the following changes to the manuscript:

1. We decided to use the term "subthreshold fluctuation range" rather than "subthreshold amplitude" (as also mentioned in Point 13 above). The revised methods contain a clearer explanation of how this parameter was quantified.
2. We update the figure caption of Fig. 2E (subthreshold fluctuation range) with the following sentence: "The subthreshold fluctuation range values (E) exceed the amplitudes observed in the mean optical waveforms (C) because it encompasses hyperpolarizations, and spikes typically emerge from a depolarized baseline."

POINT 15: Photobleaching (Line 191)

The authors report that "JEDI3sub exhibited minimal photobleaching during ~200-second imaging sessions (Fig. S3B)." However, since the methods indicate the use of "photobleaching-corrected fluorescent traces," it is unclear whether Fig. S3B represents raw or corrected data. Furthermore, photobleaching is already quantified in Fig. 1F, raising questions about the rationale for its re-presentation. The authors should clarify the data processing and the purpose of presenting photobleaching data in multiple sections.

We thank the reviewer for raising these important points. We have revised the main text to clearly state that <u>no photobleaching correction</u> was applied to the fluorescence traces used in the analyses presented in this section of the manuscript, corresponding to Figure 3 and S9-10. In addition, the revised text includes the quantification of photostability: across 13 FOVs from two animals, cells conserved 92 ± 5% of their original fluorescence over the 200-sec imaging sessions.

The rationale for presenting photobleaching data in both contexts is that photobleaching is highly dependent on experimental parameters such as laser energy per pulse, laser repetition rate, pixel dwell time, and acquisition frequency. As such, the in vitro data in Figure 1F are not sufficient to predict the degree of photobleaching under in vivo two-photon population imaging conditions. To address this, we include an independent quantification of photostability specific to each experimental paradigm used in the manuscript. This approach enables readers and future JEDI3sub/hyp users to more accurately estimate expected performance under conditions most relevant to their applications.

To clarify the rationale for discussing photostability in the context of each experiment, we included the following statement when first addressing in vivo photobleaching:

> "Because photobleaching rates are highly dependent on illumination parameters[8,29], we quantified photostability for each optical recording paradigm presented in this study."

POINT 16: Simultaneous monitoring (Lines 189 and 194)

Population Imaging (Line 189): The claim that "line scanning [enabled] the capture of voltage activity across large populations of neurons" is vague. The authors should specify the average number of neurons monitored simultaneously (including standard deviation) in the experiments that support Fig. 3.

We agree with the reviewer that this information is essential. The requested information is now included in the revised text.

POINT 17: Cell Selection (Line 194):

Although data were acquired from thousands of cells, only 95 were analyzed. The criteria for cell inclusion must be explicitly stated. Furthermore, comparing the activity of 95 selected cells with data from thousands of cells (as in reference 29) is not valid without a clear justification for such a limited analysis.

We thank the reviewer for raising this point, which helped us recognize a lack of clarity in our original presentation. Specifically, we believe the confusion arose from the transition between describing data from a representative FOV and the broader dataset, without clearly delineating their respective contexts. To address this, we have revised the manuscript to first present the representative FOV data (95 cells) before introducing the larger dataset comprising 13 FOVs (1,230 neurons), thus clarifying the narrative structure.

In the representative FOV, we analyzed 95 GEVI-expressing cells, of which 88 exhibited visually evoked subthreshold activity, and 47 demonstrated orientation tuning. From this data, we conducted pairwise correlation analysis (4,465 cell pairs in Fig. 3E–F) and the initial characterization of orientation selectivity (Fig. 3G). 4,465 is derived from the calculation of the total number of unique cell pairs (95 × 94 ÷ 2 = 4,465).

We then extended the orientation tuning analysis across the full dataset of 1,230 neurons recorded from 13 FOVs, of which 1023 neurons showed visually evoked subthreshold responses and 433 were classified as orientation selective (Fig. 3H). These numbers are explicitly stated in the revised text to avoid ambiguity.

We agree that cell inclusion criteria should be clearly articulated. While the criteria were initially described in the Methods section, we expanded this section to clarify that the exclusion criterion listed was the only one applied to this dataset.

POINT 18: Errors

Imaging Solution (Line 377): The statement "The imaging solution was the same, except that the final HEPES concentration was increased to 20 mM" is ambiguous. It should clarify whether this is compared to the formulation in XXXX (201X) and specify the initial concentration.

To address this ambiguity, and to avoid readers needing to consult another paper, we listed the full composition of the imaging solution.

Stimulation Parameters (Line 386): There is a typographical error in the description of stimulation parameters (e.g., "25 V for 1 ms, 30 V for, 30 V for 2.5 ms, …"). The authors need to carefully review and correct these details.

Thank you for highlighting the missing value. We also reviewed the entire Methods section to avoid further typos.

Typographical Errors (Line 395): The sentence, "If there was more than 1 well per variant on a plate each well was then be averaged together," contains a grammatical error. It should read, "If more than one well per variant was present on a plate, the data from each well were averaged together."

Thank you. We rephrased as suggested.

POINT 19:

Physical Units: Parameters such as the “detectability budget” in line 399 are defined without physical units.

Please see our discussion in response to Point 8.

Photostability: There is no definition or quantification of photostability, nor a reference to the procedure used.

Please see our discussion in response to Point 8. (Photostability and the detectability budget have arbitrary units. We revised our Methods to clarify how these metrics were calculated.)

POINT 20:
Background Correction and AUC Calculation (Line 514): The method for calculating the area under the curve (AUC) lacks a description of the time window used for the calculation. More comprehensive details are needed to ensure reproducibility.

Fixed. AUC were calculated over the entire 6-min traces. See also our response to Reviewer 3's first point.

POINT 21: Cell Transfection (Line 423):
The statement that “HEK293-Kir2.1 cells were not transfected and plated the day before patching” requires further explanation. The significance of this methodological choice should be discussed.

We agree with the reviewer that this sentence was confusing. We meant that this experiment did not require transfection. We removed this clause and rephrased more simply as “HEK293-Kir2.1 were plated the day before patching.”

REVIEWER #3

This paper introduces two new genetically expressed voltage indicators (GEVIs), which were optimized from their precursor JEDI-2P using a targeted screen and rationale design. The aim was to overcome limitations in optical detection and characterization of subthreshold membrane potential (Vm) changes. This goal is important because Vm changes are essential for neuronal integration and neural population dynamics. The development and characterization of two selected JEDI3s ('sub' and 'hyp') is well presented, following closely up on the previous report of JEDI-2P. In addition, a thorough comparison of JEDI3sub and JEDI3hyp to JEDI-2P is provided both in vitro and in vivo. A rudimentary comparison to ASAP5 is mentioned in the text but no example traces are provided. In the remaining paper, proof-of-concept data are given for three example applications methods (in vivo population imaging, SWR-related PV-interneuron activity, and brain-state related oscillation changes) using state-of-the-art fast 2P imaging. All three examples clearly demonstrate the capability of the new JEDI3s to provide useful subthreshold Vm information.

Overall, I find this an excellent manuscript, well-written, quantitative, and with good demonstrations of the new JEDI3 indicator subtypes. It is a significant tool optimization and I expect that it will have an impact on the field as many labs are awaiting exactly such improved versions of GEVIs capable of measuring subthreshold Vm.

We sincerely thank the Reviewer for their thoughtful and encouraging feedback. We are pleased that the manuscript was received as well-written and quantitative, and that the advances in the JEDI-3 indicator subtypes were recognized as a meaningful optimization. We greatly appreciate the Reviewer's perspective on the potential impact of these improved GEVIs for measuring subthreshold membrane potential, and we also believe they will indeed prove valuable to the broader neuroscience community.

I do have a few points for possible improvements, though:

POINT 1: In Fig. 1E, it remained unclear to me what the AUC metric refers to. Please make more explicit what exactly is shown here.

As context, fluorescent proteins and biosensors often display photobleaching kinetics that do not scale linearly —or quadratically under two-photon (2P) excitation— with illumination irradiance or power. Additionally, accurately modeling these decay traces typically requires multi-exponential fitting, with the number and relative weights of components varying depending on the illumination conditions. This complexity makes direct comparisons of fitted decay constants across power levels extremely complex. Alternatively, photobleaching could be quantified by measuring fluorescence at the final timepoint. However, this approach comes with trade-offs. For example, a sensor that rapidly decays to 10% of its initial fluorescence and remains at that level for the rest of the time course, would appear equally photostable to one that decays gradually to the same level over the full recording period, despite very different photostability profiles.

To address these limitations, we instead quantify photostability by calculating the area under the curve (AUC) of the normalized fluorescence vs. time traces. This metric provides a robust, model-independent measure that reflects the total fluorescence signal retained over the 6.05-minute duration of imaging, integrating both the rate and extent of bleaching. Higher AUC values correspond to greater sustained fluorescence and, by extension, improved photostability.

- We swapped panels E and F, so the photobleaching trace at 37 mW (previously panel F) now appears first, serving as a visual reference for the quantitative analysis in the next panel.
- We changed the y-axis label from "Area under the curve" to the more descriptive term "% photostability".
- We normalized AUC values (to the maximal achievable AUC) to make the interpretation more intuitive: 50% photostability indicates that an indicator produced 50% of the fluorescence it would have emitted if it were infinitely photostable (non-bleaching).
- We revised the figure caption to include a brief explanation of how photostability was calculated from the raw photobleaching traces.
- We revised the Method with more details on these calculations and their rationale.

POINT 2: Figure 2. In the text, numbers are given for a comparison with ASAP5 using ULoVE imaging. It would be very useful to show comparative example traces in a Suppl. Figure, together with this quantification.

We agree with the Reviewer and have added a new supplementary figure (Fig. S8) that presents representative example traces and detailed quantitative comparisons between ASAP5 and the JEDI indicators. In addition, we now include in vitro

comparisons in Fig. S4. The main text has also been updated with a revised Results section that provides a more detailed discussion of these comparisons. In brief, we demonstrate that ASAP5 has comparable performance to JEDI-2P, and that JEDI3 indicators outperform ASAP5. For further context, we refer the Reviewer to our response to point 1 from Reviewer 2.

POINT 3: Regarding population imaging (Fig. 3). I am wondering about the cell segmentation problem. Even with soma-targeted expression, cell membranes might be difficult to distinguish when cells touch each other (as some do in Fig. S3). For L2/3 it might be OK but for more tightly packed cells it may become a serious issue. Can the authors please mention that issue and comment on it in the text?

We thank the Reviewer for raising this critical point. In response, we have added a paragraph to the Discussion section describing the challenges of cell segmentation at higher labeling densities:

> *Temporal undersampling of action potentials can reduce their recorded amplitudes, potentially causing them to resemble subthreshold events—a form of artifact known as aliasing. Increasing the acquisition rate can mitigate these artifacts by improving temporal resolution. However, this often comes at the expense of spatial coverage, limiting the number of neurons that can be imaged simultaneously. In the visual cortex, as shown in Fig. S9D, cell density is moderate, and membrane overlap between neurons is relatively limited with minimal contact between cell masks. In contrast, brain regions with higher cell body density, such as hippocampal CA1, pose greater challenges for spatial resolution. In such cases, overlapping membranes may lead to signal loss (if overlapping pixels are excluded) or signal contamination (if retained), although demixing algorithms may help alleviate this issue. Alternatively, strategies to sparsify GEVI expression can reduce overlap, but this comes at the cost of recording fewer neurons simultaneously.*

POINT 4: In Fig. 4, I am puzzled by the example traces in panel C. I understand that the frame rate of imaging was too low to resolve PV spiking (which is probable) during SWR periods, however would one not expect at least increased variance in these traces (similar to the LFP measurement)? Especially since recording frequency and SWR frequency seem similar here, which will lead to aliasing but should show increased variance.

We appreciate the reviewer's thoughtful observations and questions. We do see increased variance (Rebuttal Fig. 3.3.4A and B, below)

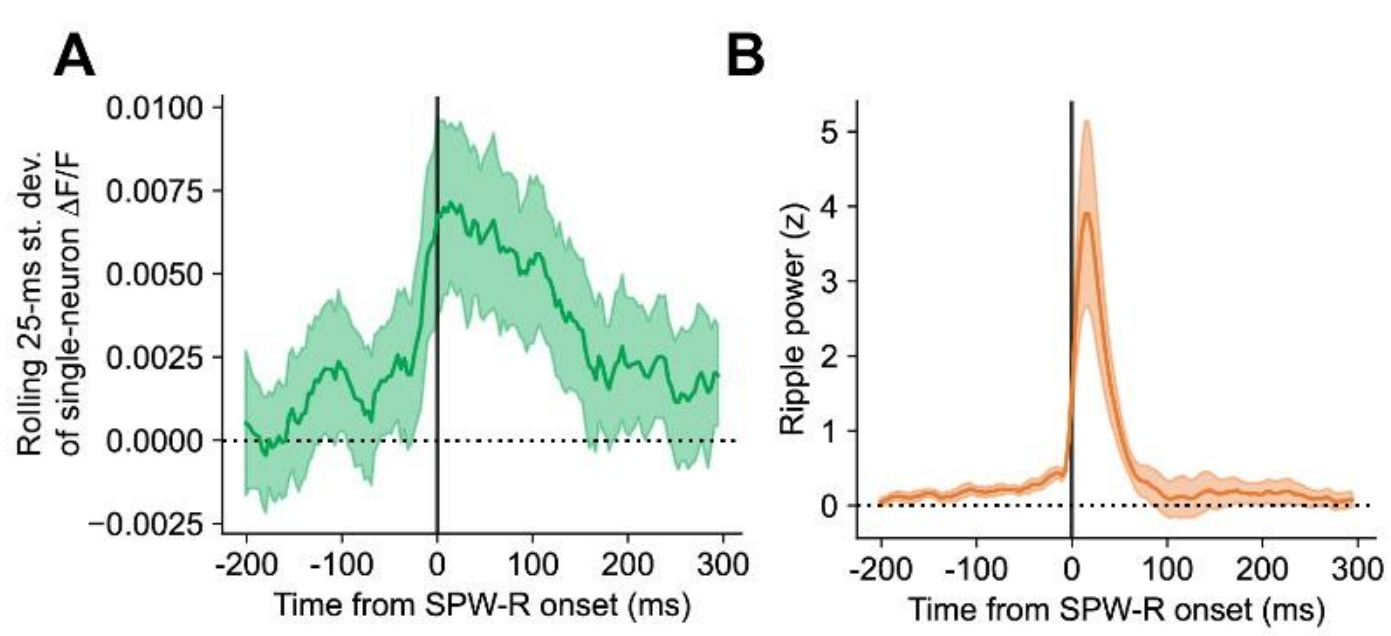

> **Rebuttal Figure 3.3.4.** (**A**) Increased variability of GEVI traces during ripples. Data from neuronal traces from Fig. 4. The standard deviation of traces ($\Delta F/F_o$) was measured using a 25-ms-wide symmetrical sliding window. The ripple-triggered average of these time series was computed in the same way as Fig. 4D. (**B**) The average ripple power is shown for comparison (reproduction of Fig. 4E).

Do the authors think that the specific recorded PV cells were spiking or possibly not? Did they see other examples with clear spiking? Please comment and address this issue in the text.

We thank the reviewer for these interesting questions. We did not observe clear examples of spiking in the recorded PV+ cells, which is consistent with our expectations given both the intrinsic properties of PV interneurons and the limitations of our imaging approach.

PV+ neurons are known to generate exceptionally narrow action potentials, with full widths at half maximum of approximately 0.3 ms —among the briefest in the mammalian brain (Aldohbeyb et al., 2021; https://doi.org/10.1371/journal.pone.0247242). While some of the authors have previously demonstrated that ULoVE-based optical recordings using the GEVI ASAP3 at 5 kHz can detect PV+ spikes, the signal-to-noise ratio (SNR) was significantly lower than for pyramidal neurons (Villette et al., 2019; 10.1016/j.cell.2019.11.004). JEDI3 indicators offer faster kinetics and larger response amplitudes than ASAP3, and therefore likely produce larger signals in response to PV spikes. However, the reliable detection of such brief events still requires ultra-fast, high-SNR imaging techniques, such as ULoVE.

In contrast, while the resonant scanning approach used in this study can track membrane potential changes, this approach presents several constraints for detecting action potentials from PV interneurons. First, fewer than 2% of the laser dwell time is dedicated to pixels within each target cells' region of interest, reducing the effective SNR. Second, the per-frame duration in our recordings was 3.5 ms —an order of magnitude longer than the duration of PV spikes. Optical signals produced by JEDI indicators in response to pyramidal cell spikes are less than 1.5 ms, and PV cells have even shorter spikes than pyramidal cells. Thus, the duration of JEDI signals is also expected to be shorter than the frame duration. This temporal mismatch is expected to result in aliasing: spikes are likely to occur outside the window during which the membrane is scanned or to be only partially sampled. Consequently, some spikes will be missed entirely, and those that are captured appear attenuated. These limitations, combined with the inherently low SNR for fast-spiking interneurons under our imaging conditions, explain why we neither observed nor expected robust spike detection.

We have now clarified this point in the main text with the following sentences:

> *Spikes were not detected, as anticipated given our experimental design. The field of view was intentionally set large enough to capture multiple cells per trial, resulting in a per-frame duration of 3.5 ms. This timeframe is substantially longer than the expected optical spike response from PV interneurons, which is likely under 1 ms since PV cells have narrower spikes than the pyramidal neurons that we recorded in Fig. 2G (~0.3 vs ~0.8 ms). Additionally, JEDIhyp-Kv-positive pixels from each cell represented 1.30 ± 0.31% of the total FOV, further limiting the achievable signal-to-noise ratio for detecting brief events such as action potentials.*

We also added a sentence to clarify that spike aliasing may be contributing to the depolarization signals we captured:

> *These depolarizations likely reflect a mixture of subthreshold and suprathreshold activity, as the temporal resolution of our imaging system will cause spike-related signals to be aliased*

Aliasing is also now discussed in the Discussion. Finally, we added this sentence to the Discussion, in the section on "Limitations, future directions, and broader implications":

> *Faster on-kinetics would improve the detection of narrow action potentials, particularly those generated by fast-spiking PV interneurons.*

Given our deliberate focus on subthreshold membrane potential dynamics and the technical limitations described above, we concluded that an analysis of PV spiking was beyond the scope of this study. Accordingly, we decided not to include the variance graph referenced earlier in our response to this point.

POINT 5: Figure 5 is interesting; however, presenting more raw data would be useful here to give the reader a better impression (and intuition) of the JEDI3 performance. Only one trace is shown in B, for which it is not even clear whether it is from soma or dendrite (or VIP?). Examples of dendritic and somatic JEDI3 traces, as well as of JEDI3 traces for VIP interneurons, should be shown in this figure.

We revised Fig. 5B to clarify that the trace was obtained from the soma. We retained the original caption clarifying that the trace is from a "layer-5 intratelencephalic pyramidal neuron (L5 IT-PN)"

We agree that representative single-trial traces from pyramidal cells' soma and dendrites, and VIP somas, would be valuable. Those are now provided in Fig. S12C, S14A, and S15B

For the L5 neuronal pool, please also provide information about soma depths and the laser power levels used to image deep somas.

We agree this is valuable information, and we have provided it in Supplementary Table 1, which also includes critical acquisition parameters for all the main figures in the manuscript.

POINT 6: The discussion can be improved. Specifically, I was missing a more thorough comparison with previous or alternative attempts to optically record subthreshold Vm, including single-photon imaging approaches.

We thank the Reviewer for this valuable suggestion. We agree that a more thorough comparison with previous or alternative approaches to optically record subthreshold membrane potential—including single-photon imaging methods—adds important context. To address this, we have incorporated a concise overview of these efforts into the Introduction, where it helps frame the motivation and significance of our work. Due to the strict word limits for Nature Methods, we were unable to expand this discussion further in the main text. However, one of the authors (F.S.P.) is currently preparing a comprehensive review article on voltage imaging, which will explore this topic in greater depth.

MINOR POINTS

- l.372: ‘expressing’

Fixed. Thank you.

- l.434, step sizes rather -80, -75, -70, …

Thanks for highlighting these critical typos. The correct list was “-75, -65, -60, etc” (-80 mV was also determined, but in the first set of voltage steps, as described in the methods)

RESPONSE TO REVIEWS

Title: "Designer indicators for two-photon recording of subthreshold voltage dynamics" (NMETH-A59507A)

REVIEWER #2

Overview

With the authors' cooperation (21 reviewer critiques and more than 21 author responses) the quality of the manuscript has improved substantially. My assessment of the revised version is largely positive; however, the final four points (A07, A08, A09, and A10) warrant the Editor's immediate attention.

We thank the reviewer for the positive assessment of our revised manuscript.

Point A1-A6

Point A1. I decided to believe the author's claims that in vitro and in vivo data demonstrate that, under 2P excitation, JEDI3sub and JEDI3hyp outperform ASAP5 in their responses to subthreshold voltage changes and in vitro brightness, while exhibiting similar spike responses.

Comparisons of the 2 indicators conducted in supplemental figures (Fig. S4) are useful (Fig. S8).

Point A2. I accept the author's claim that JEDI3sub/hyp and JEDI-2P were optimized for 2P, while ASAP3/5 were optimized for 1P.

The authors have updated the text to clarify the advancements brought by JEDI3 indicators over ASAP5 and other state-of-the-art indicators.

Point A3. I accept the author's claim that Hao et al. (2024) was not included in the reference list by a glitch in the reference manager.

Point A4. The authors have incorporated the suggested citations (voltage imaging of subthreshold signals) into the Introduction. These citations are most directly related to the current manuscript.

Point A5. I decided to believe the authors when they state that the observed signals in dendrites reflect genuine voltage fluctuations rather than artifacts such as motion or noise.

Point A6. It is true that the reviewer wanted to examine the single-trial, single-dendrite fluorescence traces that were used to generate plots such as Fig. 5F. To address this, the authors have now included recordings that show correlated subthreshold signals from two branches of a bifurcating apical dendrite segment (Fig. S14A). Critically, a nearby autofluorescent bleb does not exhibit these fluctuations, reinforcing the interpretation that the JEDI3hyp signals reflect dendritic voltage changes rather than motion artifacts or noise.

These points indicated that the reviewer agreed with our responses and did not require rebuttal.

Point A7

The absence of simultaneous whole-cell patch-clamp recordings, widely regarded as the gold standard for validating optical voltage signals, remains a notable limitation, particularly given the authors' demonstrated expertise with in vivo whole-cell recordings.

The reviewing editor should consider the following 4 facts:

1. The reviewers regard simultaneous whole-cell patch-clamp as the gold standard for validating optical voltage signals.

2. In their rebuttal, the authors likewise state that it is the gold standard.
3. However, the current, high-profile study did not include simultaneous whole-cell patch-clamp validation. None.
4. Members of the author team have established in vivo whole-cell expertise (check the list of authors), but did not apply it here.

These 4 points materially affect the strength of the study's conclusions and warrant clarification or additional validation.

We appreciate the reviewer's thoughtful evaluation of our manuscript and recognize their perspective. It is challenging to fully address this specific critique, as an explicit rationale for undertaking such extraordinarily difficult in vivo experiments was not provided. While technical expertise enables the execution of complex experiments, it does not, in itself, justify their necessity.

We are grateful that the reviewer expressed satisfaction with our new data, which confirm that the optical traces represent genuine voltage signals (e.g., see Reviewer's points A1, A2, A5, and A6 in this second round). Furthermore, we note the reviewer's acknowledgment that our indicator demonstrates improved performance compared to the current state-of-the-art (Point A2).

In light of these points, we believe we have fully addressed the reviewer's concerns.

Points A8 & A9

The manuscript does not report the robust glutamate-mediated dendritic plateau potentials described by Kerlin et al. (2019). This omission may reflect limitations in the ability of the JEDI3 variants to accurately track dendritic voltage dynamics.

If JEDI3 possessed adequate sensitivity, these sustained dendritic depolarization events would likely produce prominent voltage changes readily detectable in optical recordings.

Unfortunately, the absence of such signals in their data likely reflects problem with indicator sensitivity rather than the rarity of such biological events in experimental conditions.

In their rebuttal the authors attempted to justify the poor sensitivity of their optical recordings (JEDI3) by a statement suggesting that dendritic plateau potentials DO NOT occur in the visual cortex

Specifically the authors stated:

"In contrast, our recordings were conducted in the visual cortex during spontaneous activity, under conditions where such behaviorally driven, localized dendritic plateaus are not known to occur".

In reality, dendritic plateaus have been documented in the visual cortex.
1. Valerio Francioni, Zahid Padamsey, Nathalie L Rochefort (2019).
2. Spencer L Smith, Ikuko T Smith, Tiago Branco, Michael Häusser (2013).

Therefore, Nature Methods must NOT accept the omissions made by Land et al. in respect to dendritic plateau potentials.

Land et al. should address the absence of plateau potentials in proper experiments, or at least in the text of the article.

We thank the reviewer for their thoughtful comments and the opportunity to clarify our rationale and findings. Our benchmarking experiments were not designed to assess the ability of indicators to detect dendritic plateau potentials. Consequently, we did not employ the specific stimulation paradigms described in Kerlin, Francioni, or Smith, which rely on evoked responses. Instead, our dendritic voltage imaging focused exclusively on spontaneous activity, without external stimulation. Variations in dendritic activity are unsurprising given differences in experimental conditions.

The extended calcium transients reported by Kerlin et al. are indeed large and prolonged (>1 s), and we agree that such events should be readily detectable—likely even more so than many of the smaller transients we report. Importantly, we have demonstrated that JEDI3hyp-Kv can resolve extremely subtle voltage changes. For example, using two-photon resonant-scanning microscopy, we showed that it can optically report activity patterns (Fig. 5A, S12A) even when the underlying voltage changes average less than 3 mV.

We fully agree that persistent dendritic depolarizations are biologically important and merit further investigation. While these events were outside the scope of our current methodological study, we believe JEDI3 indicators represent a promising tool for future work aimed at understanding dendritic integration in vivo. Foundational studies from the Antic lab using the GEVI ArcLight in slices have already laid important groundwork for this line of inquiry. To acknowledge this potential, we propose adding the following sentence to the Discussion (in bold):

> *These results demonstrate the value of JEDI3 sensors to uncover novel neuronal dynamics, opening new avenues for understanding the roles of subthreshold activity in information processing.* ***Of particular interest is their application for understanding dendritic activity, including the role of extended ('plateau') depolarizations in dendritic integration (Kerlin et al., 2019; Milicevic et al., 2024).***

Point A10

In their rebuttal letter, the authors stated that they accepted the reviewer's argument for proper terminology "Population Voltage Imaging" vs. "Multi-Cell Imaging".
However, in line 54, the authors continue to confuse two terms: "Population Imaging" vs. "Multi-Cell Imaging".
Furthermore, in line 63, the authors continue to confuse two terms: "Population Imaging" vs. "Multi-Cell Imaging".
Furthermore, in line 931, the authors continue to confuse two terms: "Population Imaging" vs. "Multi-Cell Imaging".
Land et al. should correct these errors in terminology.

Lines 54 and 63: We are citing references that include population voltage imaging experiments as defined by the reviewer.

Line 931. We missed this usage of "population voltage imaging" in the Methods section. We will rephrase as follows:

- *Original*: We imaged the population subthreshold voltage activity from L2/3 neurons using a custom fluorescence microscope.
- *Revised*: We imaged subthreshold voltage activities from a group of L2/3 neurons using a custom fluorescence microscope.

REVIEWER #3

Remarks to the Author:
The authors present a thorough and comprehensive revision of their manuscript, including additional data, further analyses, and clarifications in the text. They have addressed all my specific points adequately. I have no further comments and congratulate the authors on an excellent paper.

Thank you for your positive remarks!
